# The longevity-promoting factor, TCER-1, widely represses stress resistance and innate immunity

**DOI:** 10.1038/s41467-019-10759-z

**Published:** 2019-07-17

**Authors:** Francis R. G. Amrit, Nikki Naim, Ramesh Ratnappan, Julia Loose, Carter Mason, Laura Steenberge, Brooke T. McClendon, Guoqiang Wang, Monica Driscoll, Judith L. Yanowitz, Arjumand Ghazi

**Affiliations:** 1 Departments of Pediatrics, Developmental Biology and Cell Biology and Physiology, University of Pittsburgh School of Medicine; John G. Rangos Sr. Research Center, Room 7129, One Children’s Hospital Drive, 4401 Penn Avenue, Pittsburgh, PA 15224 USA; 2 Magee-Womens Research Institute, Department of Obstetrics, Gynecology, and Reproductive Sciences, University of Pittsburgh School of Medicine, 204 Craft Avenue, Pittsburgh, PA 15213 USA; 3 Department of Molecular Biology and Biochemistry, Rutgers, The State University of New York, Nelson Biological Labs, Room A232, Piscataway, NJ 08854 USA

**Keywords:** Stress signalling, Ageing, Caenorhabditis elegans, Antimicrobial responses

## Abstract

Stress resistance and longevity are positively correlated but emerging evidence indicates that they are physiologically distinct. Identifying factors with distinctive roles in these processes is challenging because pro-longevity genes often enhance stress resistance. We demonstrate that TCER-1, the *Caenorhabditis elegans* homolog of human transcription elongation and splicing factor, TCERG1, has opposite effects on lifespan and stress resistance. We previously showed that *tcer-1* promotes longevity in germline-less *C. elegans* and reproductive fitness in wild-type animals. Surprisingly, *tcer-1* mutants exhibit exceptional resistance against multiple stressors, including infection by human opportunistic pathogens, whereas, TCER-1 overexpression confers immuno-susceptibility. TCER-1 inhibits immunity only during fertile stages of life. Elevating its levels ameliorates the fertility loss caused by infection, suggesting that TCER-1 represses immunity to augment fecundity. TCER-1 acts through repression of PMK-1 as well as PMK-1-independent factors critical for innate immunity. Our data establish key roles for TCER-1 in coordinating immunity, longevity and fertility, and reveal mechanisms that distinguish length of life from functional aspects of aging.

## Abstract (web summary)

Resistance to stress is often associated with increased longevity. Using the model organism *C. elegans* the authors here show that TCER-1 enhances lifespan while at the same time increasing sensitivity to a number of biotic and abiotic stressors.

## Introduction

In many organisms, a positive correlation has been noted between increased longevity and enhanced tolerance against environmental stressors such as high temperature, oxidative damage and pathogen attack (reviewed in refs. ^1–3^). Indeed, stress resistance has been used as a surrogate for lifespan extension in model organisms to identify several longevity genes^4–6^. However, mutants that exhibit increased lifespan without enhanced stress resilience, and vice versa, have been reported intermittently in literature. In fact, in yeast, nematodes, flies and plants, only a fraction of mutants selected for increased stress resistance also exhibit enhanced longevity^4–8^. This incomplete correlation implies that stress resistance alone is not sufficient to extend lifespan; other unknown process(es) may be induced coordinately with stress resistance in many long-lived mutants which may underlie their longevity. Importantly, these observations, and other emerging evidence, suggest that stress resilience is physiologically distinct from lifespan^9–12^. This is an important distinction because stress resilience is also a major determinant of “healthspan”, the multiparametric measure of overall health in aging animals^13–15^. With increasing emphasis on healthspan in the aging field, it is especially exigent to identify genetic and molecular pathways that uncouple stress resistance from lifespan. However, most known longevity-promoting genes also increase stress resistance (reviewed in ref. ^1^). Genes that promote longevity but widely inhibit stress resistance or other aspects of healthspan have not been identified.

There is widespread evidence from many species that increased reproduction is accompanied by reduced stress resistance, especially immune resistance, and reciprocally, pathogen infection impairs fertility (reviewed in ref. ^16^). But, while fertility and immunity appear to be mutually antagonistic, both diminish with age. Advanced maternal age is a major cause of reduced human reproductive fitness^17^. Immunosenescence, the loss of immune resistance with age, underlies increased morbidity and mortality in older organisms^18^. Hence, age is an important consideration in the immunity−fertility dynamic, but the molecular mechanisms governing this tripartite relationship are poorly understood.

The nematode *Caenorhabditis elegans*, similar to other organisms including humans, faces numerous stressors at the cellular (e.g., protein damage) and organismal (e.g., pathogens, high temperatures) levels, and responds via conserved, well-characterized systems such as the heat-shock response (HSR), oxidative stress response (OSR), hypoxia response (HR), unfolded protein response in the mitochondria (UPR^mt^) or endoplasmic reticulum (UPR^er^) and others (reviewed in refs. ^19,20^). The response to pathogen threat in *C. elegans* is spearheaded by an innate immune system that includes conserved signaling pathways such as the mitogen-activated protein kinase (MAPK) cascade. The *C. elegans* p38 MAPK, PMK-1, activated by pathogenic stimuli as well as other stressors such as oxidative damage, governs the activity of multiple transcription factors to facilitate pathogen-specific responses^21^. Significant overlap exists between innate immune responders and other canonical stress-response factors. For instance, ATFS-1, SKN-1, HSF-1 and HIF-1, key mediators of UPR^mt^, OSR, HSR and HR, respectively, also upregulate innate immunity genes and confer pathogen resistance^22–26^. Many of these proteins, and other such stress-response mediators, also enhance lifespan in *C. elegans* and other species^22,23,27–30^. Additionally, their inactivation not only shortens lifespan but accelerates age-related decline in morphology, physiology and behavior i.e., healthspan parameters^31,32^. So, while studying such factors has enriched our knowledge of stress-response mechanisms and longevity paradigms immensely, it has not advanced discovery of the molecular distinctions between the quantitative and qualitative measures of aging. In this study, we describe a role for TCER-1, *C. elegans* homolog of the human transcription elongation and splicing factor, TCERG1^33,34^, in having discrete and opposite impacts on longevity and stress resilience.

We first identified TCER-1 as a factor essential for the lifespan extension caused by germline loss in *C. elegans*^35^. In *C. elegans*, removal of the totipotent population of germline-stem cells increases lifespan, dependent on a network of transcription factors including TCER-1 and the conserved longevity determinant, DAF-16/FOXO3A (reviewed in ref. ^36^). TCER-1 overexpression increases the lifespan of normal, fertile animals underscoring its role as a pro-longevity gene^35^. Recently, we showed that TCER-1 and DAF-16 act coordinately to extend the lifespan of germline-less animals by preserving lipid homeostasis^37^. We also discovered that, in normal fertile *C. elegans*, TCER-1 is critical for optimal reproduction as well as prevention of age-related reproductive decline. *tcer-1* mutants produce fewer, and less viable, eggs than their wild-type counterparts, and exhibit signs of premature reproductive senescence. Thus, under normal physiological conditions TCER-1 promotes reproductive fitness^37^.

In this report, we demonstrate that TCER-1 inhibits resistance against multiple biotic and abiotic stressors, including immunoresistance against the opportunistic Gram-negative human pathogen *Pseudomonas aeruginosa* and the Gram-positive pathogen *Staphylococcus aureus*. *tcer-1* mutants show increased survival upon infection and, reciprocally, TCER-1 overexpression increases susceptibility towards *P. aeruginosa*. TCER-1 acts cell non-autonomously in somatic tissues to mediate both its anti-immunity and prolongevity functions. We also find that TCER-1 inhibits immunity during the fertile stages of life and not in post-reproductive animals. Additionally, elevating TCER-1 levels partially arrests the decline in progeny production that follows infection, suggesting that TCER-1 may repress immunity to promote reproductive fitness. *tcer-1* mutants exhibit increased expression of numerous known, as well as novel, antibacterial genes, at least four of which are essential for protection against infection. Our data suggest that TCER-1 inhibits immunoresistance by repressing PMK-1, as well as PMK-1-independent, innate immunity pathways. They reveal TCER-1 as a key factor governing the relationship between the intimately linked processes of immunity, lifespan and fertility.

## Results

### TCER-1 widely suppresses stress resistance

**Fig. 1:**
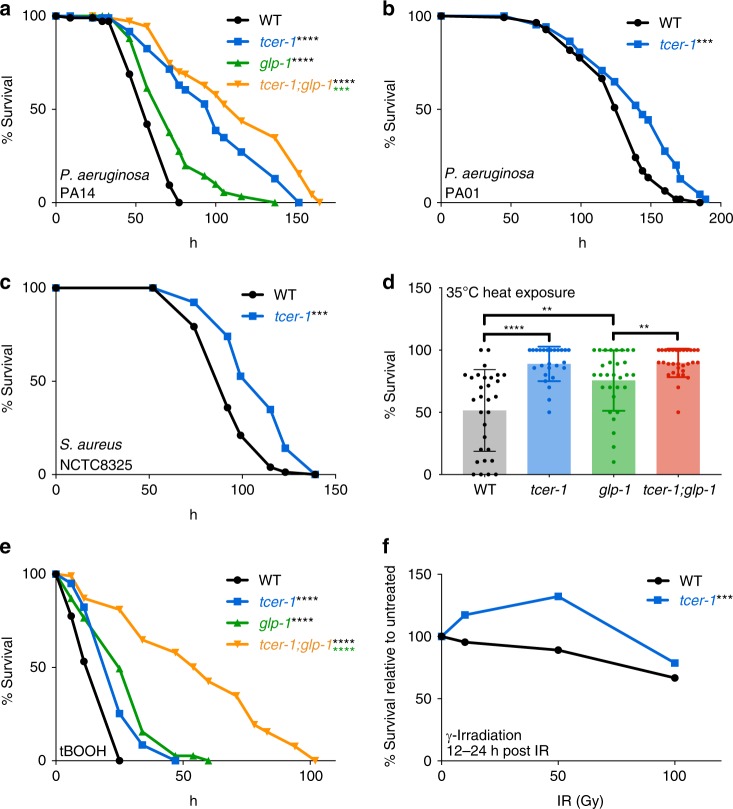
Loss of TCER-1 enhances resistance against multiple biotic and abiotic stressors. Survival of L4 stage, wild-type *C. elegans* (WT, black), *tcer-1* (blue), *glp-1* (green) and *tcer-1;glp-1* (orange) mutants exposed to different stressors. **a**–**c** Pathogen exposure. **a** *P. aeruginosa* PA14: WT (*m* = 59.35 ± 1.3, *n* = 89/106), *tcer-1* (*m* = 98.6 ± 3.5, *n* = 83/109, *P* vs. WT <0.001), *glp-1* (*m* = 72.86 ± 2.1, *n* = 102/109, *P* vs. WT <0.0001) and *tcer-1;glp-1* (*m* = 114.18 ± 3.9, *n* = 81/106, *P* vs. *glp-1* <0.001). **b** *P. aeruginosa* PA01: WT (*m* = 126.43 ± 2.4, *n* = 118/142), *tcer-1* (*m* = 139.98 ± 2.7, *n* = 143/173, *P* vs. WT <0.001). **c** *S. aureus*: WT (*m* = 94.67 ± 1.5, *n* = 97/111), *tcer-1* (*m* = 109.27 ± 1.5, *n* = 145/169, *P* vs. WT <0.001). **d** Heat stress: survival of day 2 adults 12 h after exposure to 35 °C for 6 h. *n* = 50−100 animals per strain per each of six biological replicates analyzed using unpaired two-tailed *t* test. **e** Oxidative stress: survival of L4 animals in 7 mM t-BOOH. WT (*m* = 17.32 ± 1.1, *n* = 57/98), *tcer-1* (*m* = 25.65 ± 1.7, *n* = 33/100, *P* vs. WT <0.0001), *glp-1* (*m* = 27.88 ± 1.4, *n* = 79/100, *P* vs. WT <0.0001) and *tcer-1;glp-1* (*m* = 56.31 ± 5.1, *n* = 29/100, *P* vs. *glp-1* <0.0001). **f** DNA damage: viability of eggs laid by day 1 hermaphrodites 24 h after exposure to 50 Gy γ-irradiation, *n* = 9−20. *P* 0.003 in unpaired *t* test. In (**a**–**c**) and **e**, survival data analyzed using Kaplan−Meier test, shown as mean lifespan in hours (*m*) ± standard error of the mean (SEM). ‘*n*’ refers to number of animals analyzed/total number in experiment (see Methods for details). *P* values adjusted for multiplicity where applicable. Asterisks indicate statistical significance <0.01 (**), <0.001 (***) and <0.0001 (****) and their color denotes the strain of comparison. Data from additional trials presented in Supplementary Tables 1A, B (panel a), 2A, B (b, c), 3A (e) and Supplementary Fig. 1d (f)

TCER-1 is critical for the extended lifespan of *glp-1* mutants, the temperature sensitive, sterile model of germline-less longevity^38^. *tcer-1* single mutants exhibit a lifespan similar to normal, fertile animals^35^. Since most longevity-promoting genes also augment stress resistance, we initially hypothesized that loss of *tcer-1* would make animals more susceptible to environmental stressors. However, upon testing for resilience against the human opportunistic pathogen, *P. aeruginosa* PA14 strain (henceforth PA14), using the Slow Killing (SK) paradigm (wherein PA14 is grown on a low osmolarity, minimal medium and causes *C. elegans* to die over the course of several days^39^), we found that *tcer-1* mutants showed a dramatic increase in survival as compared to wild-type adults (Fig. 1a, Supplementary Table 1A, B). Expectedly^40,41^, *glp-1* mutants survived longer than the fertile controls, but the *tcer-1;glp-1* mutants survived even longer than *glp-1* (Fig. 1a, Supplementary Table 1A, B). In contrast, and as expected^42^, *daf-16* inactivation significantly reduced survival in both wild-type and *glp-1* strains (Supplementary Fig. 1a–c, Supplementary Table 1B). We checked if *tcer-1* mutants exhibited aberrant feeding that may explain their resistance. However, no difference was observed in the pharyngeal pumping rates of the wild-type and mutant strains, either when fed *Escherichia coli* OP50, the regular lab diet (henceforth OP50) or PA14 (Supplementary Fig. 2a). The two strains’ uptake of GFP-labeled OP50 was similar as well, although GFP-labeled PA14 uptake was slightly reduced in the mutants (Supplementary Fig. 2b). Notably, *tcer-1* mutants infected with PA14 showed a dramatic reduction in colony-forming units (CFUs) as compared to wild type (Supplementary Fig. 2c) suggesting that the enhanced survival of *tcer-1* mutants was not simply due to reduced bacterial consumption. Additionally, prior treatment with 5-fluoro-2′-deoxyuridine (FUDR), that is toxic to developing eggs and hence mitigates internal hatching (matricide or bagging), did not abolish *tcer-1* mutants’ PA14 resistance (Supplementary Fig. 2d), indicating that their increased survival could not be attributed to differences from wild type in the rate of bagging. *tcer-1* mutants also showed increased survival upon exposure to another *P. aeruginosa* strain, PA01^43^, in the SK paradigm as well as the Gram-positive pathogen *S. aureus*^44^ (Fig. 1b, c, Supplementary Table 2). Thus, loss of *tcer-1* function appeared to broadly inhibit *C. elegans* immunity.

We next asked if the dichotomy between the effect of *tcer-1* inactivation on longevity and immunity extended to other environmental challenges and found similar results with heat, oxidative stress and DNA damage paradigms. Wild type, day 2 adults exposed to 35 °C for 6 h showed ~50% survival after 12 h while *glp-1* mutants showed ~75% survivorship. *tcer-1* and *tcer-1;glp-1* mutants exhibited enhanced resistance with ~90% survival (Fig. 1d). Similarly, *tcer-1* mutants showed increased resistance to oxidative stress induced by 7 mM tert-Butyl Hydroperoxide (tBOOH) by 30−50% (Fig. 1e, Supplementary Table 3A) and were significantly more resistant to DNA damage produced by exposure to γ-irradiation (Fig. 1f, Supplementary Fig. 1d). Upon experiencing ER stress mediated by Tunicamycin exposure, the mutants did not survive significantly longer (though a trend towards increased survival was evident; Supplementary Fig. 1e, Supplementary Table 3B). Thus, *tcer-1* loss of function did not reduce resistance to stress, rather it dramatically and widely increased resilience against multiple biotic and abiotic stressors, indicating it to be a prolongevity gene that functions as a “anti-stress-resistance” factor.

### TCER-1 acts cell non-autonomously to inhibit immunity

**Fig. 2:**
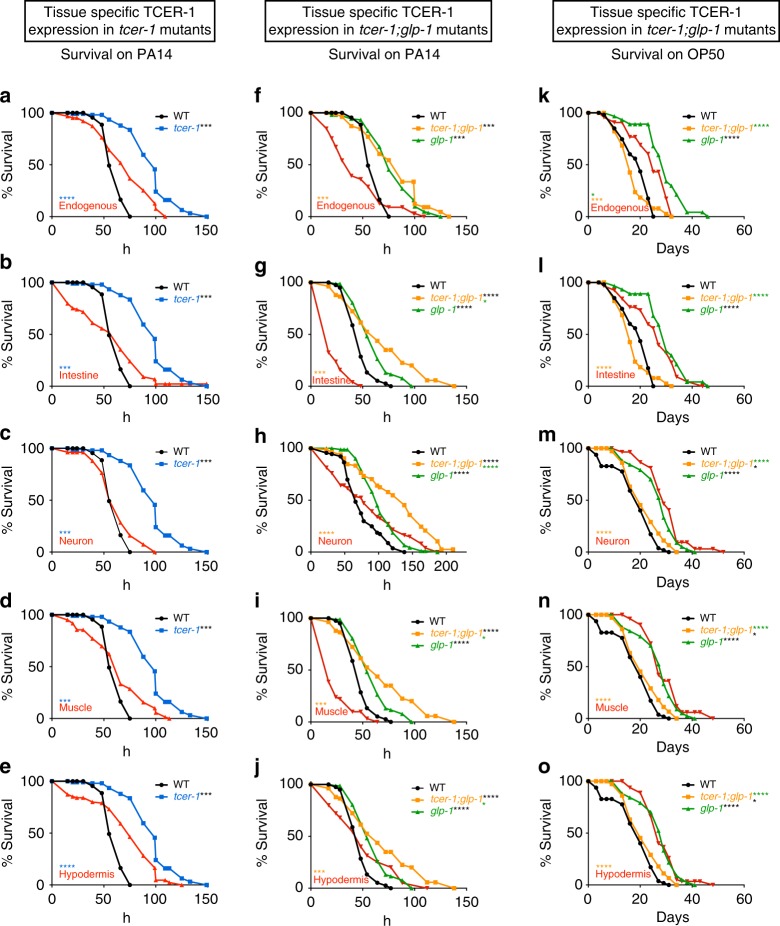
TCER-1 acts cell non-autonomously to suppress immunity and promote longevity. **a**–**j** TCER-1 expression in any somatic tissue suppresses PA14 resistance. Mean survival (hours) of L4 larvae transferred to PA14. **a**–**e** Expression in *tcer-1* mutants. Wild type (WT, black, 60.68), *tcer-1* (blue, 96.31) and *tcer-1* mutants expressing TCER-1 (red) under control of **a** endogenous promoter (70.91) or promoters expressed in **b** intestine (*gly-19*, 55.3), **c** neurons (*rgef-1*, 62.32), **d** muscles (*myo-3*, 64.0) or **e** hypodermis (*col-12*, 71.67). **f**–**j** Expression in *tcer-1;glp-1* mutants. **f** Native expression. Wild type (WT, 60.68), *glp-1* (79.2), *tcer-1;glp-1* (orange, 80.3) and *tcer-1;glp-1* mutants expressing TCER-1 under the control of endogenous promoter (44.0). **g**–**j** Tissue-specific expression. WT (47.61), *glp-1* (59.52), *tcer-1;glp-1* (67.98) and *tcer-1;glp-1* mutants expressing TCER-1 (red) under the control of promoters expressed in **g** intestine (22.45), **i** muscles (25.03) or **j** hypodermis (47.15). **h** Neurons: WT (75.3), *glp-1* (102.34), *tcer-1;glp-1* (126.59) and neuron-expressed TCER-1 (84.31). **k**–**o** TCER-1 expression in any somatic tissue extends *tcer-1;glp-1* mutant’s lifespan on OP50. Mean survival (days) on OP50. **k**, **l** WT (18.7), *glp-1* (29.32), *tcer-1;glp-1* (17.11) and *tcer-1;glp-1* mutants expressing TCER-1 under **k** endogenous promoter (24.14) and **l** tissue-specific expression in intestine (25.66). **m**–**o** WT (18.44), *glp-1* (27.35), *tcer-1;glp-1* (21.46) and *tcer-1;glp-1* mutants expressing TCER-1 in **m** neurons (29.98), **n** muscles (29.26) and **o** hypodermis (28.14). Survival data analyzed using Kaplan−Meier test. *P* values adjusted for multiplicity where applicable. Asterisks indicate statistical significance <0.05 (*), <0.001 (***) and <0.0001 (****) and their color denotes the strain of comparison. Assays in some panels were conducted in the same biological replicate so their controls are shared. Details of number of animals and data from additional trials in Supplementary Tables 4A (panels a–e), 4B (f–j) and Supplementary Data 1 (k–o)

The incongruity of *tcer-1*’s effect on lifespan vs. stress resistance could possibly be explained if TCER-1 exerted prolongevity effects in some tissues and anti-stress resistance effects in others. To test this possibility, we expressed TCER-1 in individual tissues of *tcer-1* mutants and examined the effect on their survival on PA14. Our previous studies showed that TCER-1 is ubiquitously expressed in the nuclei of all somatic tissues^35^. Expressing TCER-1 using its endogenous promoter significantly diminished the PA14 resistance of *tcer-1* mutants (Fig. 2a, Supplementary Table 4A). When it was expressed selectively in the neurons of *tcer-1* mutants (using the *rgef-1* promoter), their enhanced survival on PA14 was completely abolished. TCER-1 expression in the intestine, muscles or hypodermis (using *gly-19, myo-3* or *col-12* promoters, respectively) also drastically shortened *tcer-1* mutants’ survival on PA14 (Fig. 2b–e, Supplementary Table 4A). The enhanced PA14 resistance of *tcer-1;glp-1* mutants was also suppressed by expressing TCER-1 under its endogenous promoter or in any one of these tissues (Fig. 2f–j, Supplementary Table 4B). Indeed, TCER-1 expression in the intestine or muscles rendered the animals hyper-susceptible to PA14 (Fig. 2g, i). Thus, TCER-1 expression in any somatic tissue was sufficient to cell non-autonomously repress the increased PA14 resistance of *tcer-1* mutants.

### TCER-1 acts cell non-autonomously to promote longevity

*tcer-1;glp-1* mutants are significantly shorter-lived than *glp-1* on normal OP50 lawns^35,37^. We next checked how TCER-1 expression in individual tissues of *tcer-1;glp-1* mutants influenced their lifespan on a diet of OP50. Expectedly, endogenous promoter-driven TCER-1 rescued lifespan to *glp-1* levels (Fig. 2k, Supplementary Data 1A). Additionally, expression in the intestine, neurons, muscle or hypodermis (using the same promoters as above) also completely rescued the lifespan of *tcer-1;glp-1* mutants (Fig. 2l–o, Supplementary Data 1B–E). Thus, TCER-1 presence in any somatic tissue is sufficient to cell non-autonomously promote the longevity of germline-less adults under normal food conditions. These data also overrule the possibility that the increased PA14 susceptibility of these strains (described in the paragraph above) is a consequence of transgene toxicity or general sickness. Taken together, our results suggest that TCER-1 functions in a cell non-autonomous manner to influence both stress resistance and longevity, and it can act in any tissue to increase lifespan or reduce innate immunity.

### TCER-1 does not repress immunity in post-reproductive adults

**Fig. 3:**
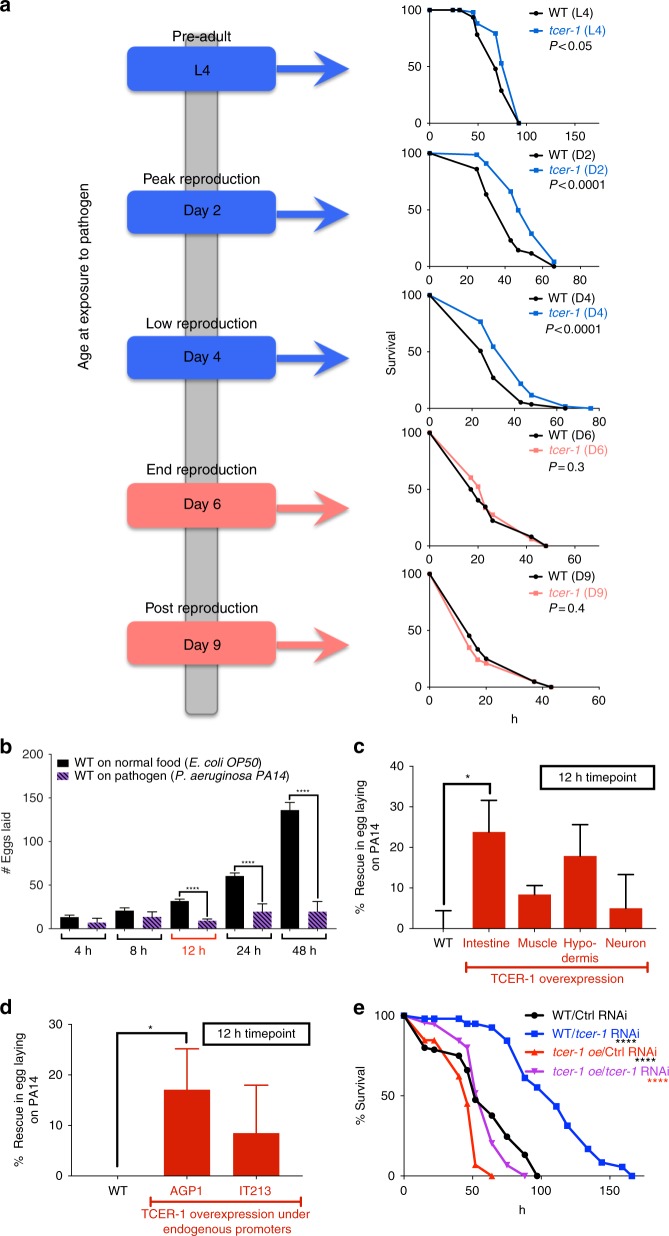
TCER-1 links fertility and immunity. **a** TCER-1 does not inhibit immunity in post-reproductive adults. Schematic on left indicates age at which animals were transferred to PA14 plates. Mean survival (hours) upon PA14 exposure as L4 larvae: wild type (WT, black: 71.69), *tcer-1* (blue: 79.97), reproductively active adults (blue): day 2: WT (40.89), *tcer-1* (51.36), day 4: WT (31.41), *tcer-1* (38.86) and at post-reproductive ages (pink): day 6: WT (24.82), *tcer-1* (26.17) or day 9: WT (20.91), *tcer-1* (19.6). **b** Fertility reduction caused by PA14. Egg-laying dynamics of late L4 larvae transferred to PA14 (purple, hashed) or control *E. coli* OP50 (black, solid). **c**, **d** Decline in egg laying caused by PA14 is limited by overexpressing TCER-1. *Y*-axes show percent rescue in egg laying 12 h after PA14 exposure by strains overexpressing TCER-1 (red bars, *X*-axes) in **c** individual somatic tissues or **d** under control of *tcer-1* endogenous promoters, as compared to the 65% reduction in egg laying shown by WT (baseline). To control for differences in brood sizes, number of eggs laid by each strain on PA14 was normalized to its brood size on OP50. For assays (**b**–**d**), data combined from 2 to 10 biological replicates with 10−20 animals per strain per replicate. Statistical significance calculated using two-tailed, unpaired *t* test. Error bars denote standard error. **e** TCER-1 overexpression increases PA14 susceptibility. Survival (hours) of wild-type on control vector (WT/Ctrl, black; *m* = 57.47, *n* = 68/100) or *tcer-1* RNAi (WT/*tcer-1*, blue, *m* = 108.46, *n* = 100/100) and endogenous promotor-driven TCER-1 transgenic strain (*tcer-1* o/e) on control (*tcer-1 oe*/Ctrl, red; *m* = 43.46, *n* = 68/85) or *tcer-1* RNAi (*tcer-1* oe/*tcer-1*, purple, *m* = 57.96, *n* = 83/116). Survival data analyzed using Kaplan−Meier test. Asterisks indicate statistical significance <0.05 (*), and <0.0001 (****) and color denotes the strain of comparison. *P* values adjusted for multiplicity where applicable. Details of number of animals in panel **a** and data from additional trials in Supplementary Table 5

Like many species, *C. elegans*’ resistance against PA14 decreases with age^45^. To determine if TCER-1’s impact on immunity was age dependent, we compared the PA14 resistance of *tcer-1* mutants as late L4 larvae (pre-adulthood), day 2 adults (peak reproduction), day 4 adults (reproductively active), day 6 adults (reproduction cessation) and day 9 adults (post-reproductive) to that of age-matched, wild-type controls (Fig. 3a). Both wild-type and *tcer-1* mutants showed a decline in survival time with increasing age, but *tcer-1* mutants survived significantly longer than their age-matched, wild-type counterparts when exposed to PA14 as L4 larvae, or day 2 or day 4 adults. However, by day 6, when most animals cease laying eggs, the mutants were not more resistant than wild type (no statistical difference in 5/7 trials). By post-reproductive Day 9, *tcer-1* mutants were either as susceptible to PA14 as wild-type animals or even more sensitive (Fig. 3a, Supplementary Table 5). Thus, *tcer-1* mutants exhibited increased survival on PA14 during the fertile stages of adulthood; its impact coincided with, and paralleled, the reproductive profile of the animal raising the possibility that TCER-1’s functions in fertility and immunity may be related.

### TCER-1 overexpression curbs fertility loss caused By PA14

Upon pathogen attack, fertility decline and reproduction arrest are usually one of the first consequences experienced by the host in most species^16,46^. Based on our previous discovery, that TCER-1 promotes reproductive fitness and germline health in normal, fertile animals^37^, and since TCER-1 appeared to exert the strongest repression on immunity at the peak of the animal’s fertility but not during the post-reproductive phase of life, we speculated if the protein repressed stress resistance to divert cellular resources towards progeny production. To test this, we first documented how PA14 effects *C. elegans* reproduction. We assessed egg laying in wild-type animals grown on normal food for ~65-h post-hatching and then exposed to PA14 as L4 larvae. A decline in the number of eggs laid by infected animals became apparent within 8 h and by 12 h infected *C. elegans* laid ~65% fewer eggs as compared to normal adults (Fig. 3b). We next asked if, and how, the dynamics of this decline were altered if TCER-1 levels were elevated. The 12 h post-infection timepoint was selected for these experiments as this stage was early enough to exhibit a strong reduction in egg production but well before the animal was overwhelmed by infection (48 h). We compared the decline in egg laying upon PA14 exposure between wild-type adults and those overexpressing TCER-1 in the intestine, muscle, hypodermis, neurons or under control of *tcer-1* endogenous promoter. While normal animals exhibited ~65% decline in the number of eggs laid 12 h post-infection, the decline in transgenic strains was only ~35−55% (Fig. 3c, d). The protective effect was strongest, and achieved statistical significance, in a strain overexpressing TCER-1 in intestinal cells and in one of two strains wherein TCER-1 was driven by its own promoter (Fig. 3c, d). Neuronal, hypodermal and muscle overexpression showed a consistent and perceptible rescue in fertility, but did not achieve statistical significance. Thus, upregulating TCER-1 partly counteracted the fertility decline that followed pathogen attack in *C. elegans*. These observations support the premise that TCER-1 may promote the allocation of resources towards reproduction by repressing cellular investment in stress resilience.

### TCER-1 overexpression increases susceptibility to infection

If TCER-1 were indeed promoting reproduction by repressing immunity, overexpression of the protein would conversely be expected to increase susceptibility to the pathogen. Interestingly, when TCER-1 was overexpressed in individual somatic tissues of wild-type animals, no consistent alteration in PA14-sensitivity was observed (Supplementary Table 4c). But a transgenic strain overexpressing TCER-1 widely under control of its endogenous promoter^47^ showed extraordinarily high susceptibility to PA14, as compared to the wild type (Fig. 3e) despite having similar pharyngeal pumping rates as the wild-type and *tcer-1* mutants (Supplementary Fig. 2a). This susceptibility was abrogated upon *tcer-1* RNAi (Fig. 3e).

**Fig. 4:**
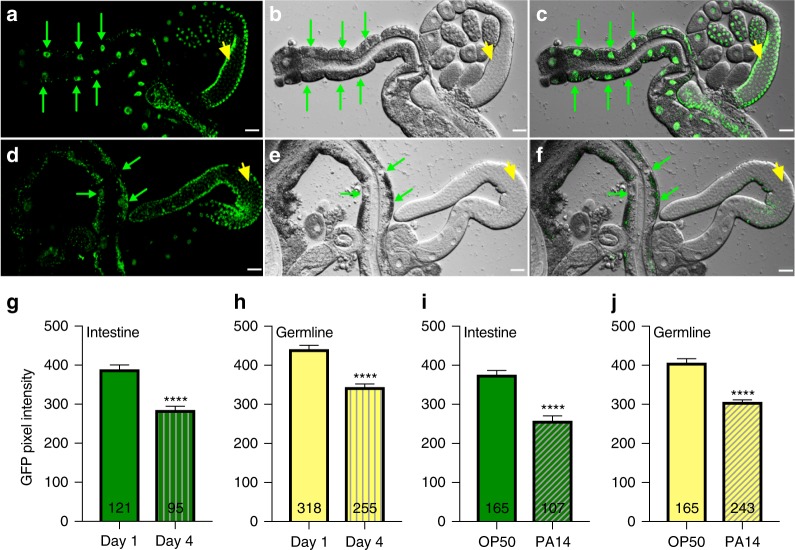
TCER-1 levels in intestine and germ cells are reduced with age and PA14 exposure. TCER-1::GFP expression in the nuclei of intestinal cells (green arrows) and germ cells (yellow arrowhead) visualized in dissected wild type, day 1 (**a**–**c**) or day 4 (**d**–**f**) *C. elegans*; the corresponding images, visualized by differential contrast interference (DIC) microscopy, and GFP-DIC overlaps, are shown in (**b**, **c**) and (**e**, **f**), respectively. By day 4, expression is significantly diminished in most intestinal nuclei (compare green arrowheads, quantified in **g**) while a more modest reduction is visible in germ-cell nuclei (quantified in **h**). Note: By day 4, some intestinal nuclei showed no visible GFP and the quantification does not take these into account. A similar reduction in GFP levels in intestinal nuclei (**i**) as well as germ-cell nuclei (**j**) was exhibited in day 1 adults 12 h after exposure to PA14 (hashed bars in **i** and **j**), as compared to age-matched controls animals on OP50 (solid bars in **i** and **j**). “*n*” signifies the total number of nuclei in which GFP intensity was measured from intestines (from 9 to 19 adults per strain per condition) and germlines (from 7 to 13 adults per strain per condition) (see Supplementary Fig. 8 for additional quantification). Asterisks represent the statistical significance of the differences in expression in an unpaired, two-tailed *t* test with *P* values <0.0001 (****). Error bars represent standard error of the mean

Since altering TCER-1 levels impacted pathogen susceptibility and fertility, it is conceivable that the protein is in turn influenced by the presence of pathogens and/or reproductive age. Indeed, we found that TCER-1::GFP expression in both the somatic tissues and germ cells was significantly diminished with age, and upon PA14 exposure. TCER-1::GFP expression was highest on day 1 of adulthood. In intestinal nuclei, expression was dramatically reduced by day 4 (Fig. 4a–g); by day 6 no GFP was detectable in any intestinal cell. In the germ cells, expression was reduced in day 4 as well, but the decline was not as conspicuous as in the intestine (Fig. 4a–f, h). Within 12 h of exposure of day 1 adults to PA14, we observed a reduction in TCER-1::GFP levels in both the intestinal nuclei (Fig. 4i) and germ cells (Fig. 4j). Interestingly, *tcer-1* mRNA levels did not change significantly upon PA14 exposure (Supplementary Fig. 7), implying the existence of post-transcriptional regulatory mechanisms.

### TCER-1 represses PMK-1 and its targets to impede immunity

How does TCER-1 repress immunity? Previously, we identified genes repressed by TCER-1 (“DOWN” class) upon germline loss^37^. Upon closer examination of this dataset, we found that ~15% of the genes with >1.5 fold expression change (42/295) were associated with immunity and stress resistance functions and 24/42 were predicted to be upregulated upon PA14 exposure (Supplementary Fig. 3a). This led us to explore the potential overlap between TCER-1 DOWN^37^ targets and genes reported to be elevated upon PA14 infection. Of the 317 and 288 genes found to be upregulated 4 and 8 h after PA14 infection, respectively, by Troemel et al.,^48^ 23 were included in the TCER-1 DOWN^37^ group (8 h exposure—*p* < 1.753e-07, Representation factor: 4.2; 4 h exposure—*p* < 1.350e-06, Representation factor: 3.6) (Supplementary Fig. 3b). Similarly, of the 197 genes Shapira et al.,^49^ reported as being upregulated upon PA14 infection, 16 were included in TCER-1 DOWN^37^ group (*p* < 2.066e-07, Representation factor: 4.9) (Supplementary Fig. 3c). Comparisons with previously identified PA14-induced genes^50,51^ yielded similar statistical overrepresentation (Supplementary Fig. 3d, e). Indeed, the TCER-1 DOWN^37^ class included *pmk-1* itself strengthening the possibility that it may inhibit the expression of immunity genes and do so through PMK-1 repression.

**Fig. 5:**
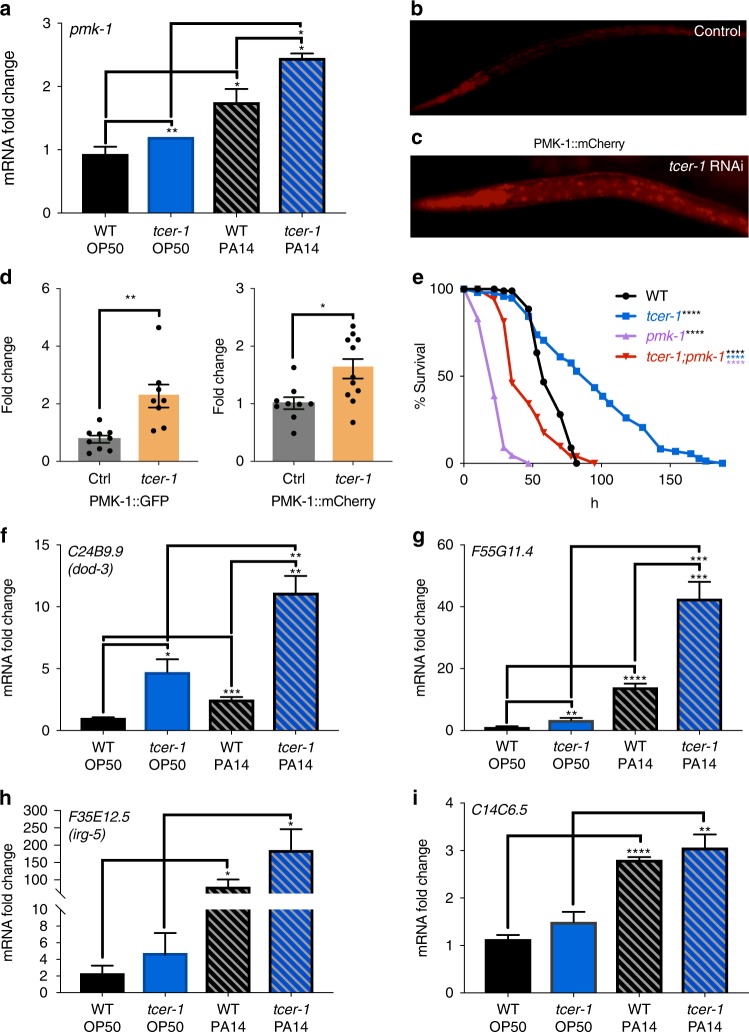
TCER-1 suppresses immunity by inhibiting PMK-1. **a** mRNA levels of *pmk-1* measured by QPCR in day 1 wild-type (black) and *tcer-1* mutant (blue) adults maintained on OP50 (solid bars) or exposed as L4s to PA14 for 8 h (hashed bars). **b**–**d** PMK-1::mCherry reporter strain grown on **b** empty control vector or **c** *tcer-1* RNAi from egg until larval stage L4 then transferred to PA14 for 24 h. **d** PMK-1::mCherry quantification from (**c**) and similar imaging of PMK-1::GFP strain upon *tcer-1* RNAi. Data combined from three biological replicates with 15–20 animals imaged per strain per replicate. **e** *pmk-1* null mutation suppresses *tcer-1* mutant’s PA14 resistance. Survival of wild-type animals (WT, black *m* = 63.49 ± 1.3, *n* = 75/100), *tcer-1* (blue, *m* = 93.82 ± 4.4, *n* = 84/100, *P* vs. WT <0.0001), *pmk-1* (*m* = 23.77 h ± 0.8, *n* = 92/100, *P* vs. WT <0.001) and *tcer-1;pmk-1* mutants (*m* = 45.95 ± 1.8, *n* = 97/102, *P* vs. *tcer-1* <0.001, *P* vs. *pmk-1* <0.0001, *P* vs. WT <0.0001) on PA14 after development on OP50 till L4 stage. **f**–**i** Expression of PMK-1 target genes is upregulated in *tcer-1* mutants. Q-PCR analysis of mRNA levels of known PMK-1-upregulated genes **f** *dod-3*, **g** *F55G11.4*, **h** *irg-5* and **i** *C14C6.5* measured after wild-type (WT, black) and *tcer-1* mutants (blue) grown on OP50 till L4 stage were transferred to PA14 plates for 12 h (hashed bars) or continued on OP50 (solid bars). Data in (**a**) and (**f**–**i**) combined from three independent biological replicates, each including three technical replicates. Error bars represent standard error of the mean (SEM). Survival data in panel (**e**) calculated using the Kaplan−Meier test and shown as mean lifespan in hours (*m*) ± SEM (see Methods for details). In (**a**, **d** and **f**–**i**) statistical significances were calculated using a one-tailed *t* test. *P* values were adjusted for multiplicity where applicable. Asterisks indicate the degree of statistical significance <0.05 (*), <0.01 (**), <0.001 (***) and <0.0001 (****) and their color denotes the strain/condition of comparison

Quantitative PCR (Q-PCR) analysis showed a significant increase in *pmk-1* mRNA levels in *tcer-1* mutants, both on OP50 as well as upon PA14 infection (Fig. 5a). Strains expressing PMK-1::GFP or PMK-1::mCherry translational reporters^52,53^ both displayed increased fluorescence upon *tcer-1* RNAi (Fig. 5b–d). To test the functional relevance of this interaction, we examined the effect of *pmk-1(km25)* null mutation on the PA14 resilience of *tcer-1* mutants. *tcer-1;pmk-1* mutants exhibited significantly reduced survival on PA14 as compared to *tcer-1* mutants alone but their resistance was not completely abolished compared to the *pmk-1* single mutant (Fig. 5e).

We next asked if the repression exerted by TCER-1 on PMK-1 impacted the expression of the latter’s target genes. A comparison of our TCER-1 “DOWN” class with genes predicted by previous studies to be upregulated by PMK-1 following PA14 infection^48,54^ also revealed a significant overlap (Supplementary Fig. 3f–h). Indeed, in Q-PCR assays, 6/7 of the top genes shared between the two groups showed increased expression 12 h after PA14 infection (Fig. 5f–i, Supplementary Fig. 4) and of these, five were upregulated in *tcer-1* mutants (three achieved statistical significance; *dod-3*, *F55G11.4* and *lys-10*). Overall, these data suggest that TCER-1 may repress pathogen resistance, in part, through inhibition of the PMK-1-dependent innate immunity pathways.

### TCER-1 lowers expression of PMK-1-independent immunity genes

**Fig. 6:**
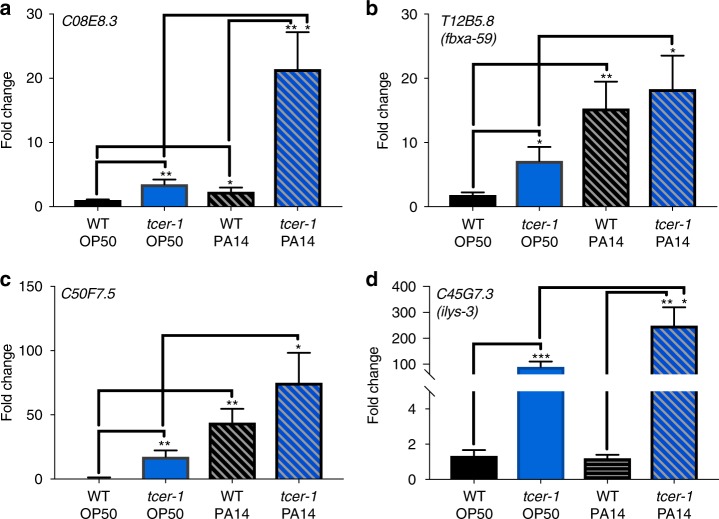
TCER-1 inhibits immunity by repressing PMK-1- independent genes. Gene expression measured by Q-PCRs of wild-type animals (black) and *tcer-1* mutants (blue) grown on OP50 till L4 stage and then exposed to PA14 (hashed bars) or retained on OP50 for 12 h. **a**–**d** Expression of PMK-1-independent immunity genes *C08E8.3* (**a**), *fbxa-59* (**b**), *C50F7.5* (**c**) and *ilys-3* (**d**). Data combined from 2 to 5 independent biological replicates, each including three technical replicates. Statistical significance of the differences in expression was calculated using a one-tailed *t* test. Error bars represent standard error of the mean. *P < 0.05, **P < 0.01, ***P < 0.001

Our *pmk-1;tcer-1* epistasis data suggested that the PA14 resistance of *tcer-1* mutants could only be partially explained by PMK-1 repression. In addition, 17/42 TCER-1-repressed immunity genes were expected to be induced in a PMK-1-independent fashion (Supplementary Fig. 3a). Hence, we explored the regulation of the PMK-1-independent genes predicted to be repressed by TCER-1. In accordance with the RNA Seq data, 7/8 of the top genes from this class that we tested showed elevated expression in *tcer-1* mutants and 6/8 were also upregulated in wild-type adults upon PA14 exposure (five achieved statistical significance; *C08E3.8*, *C50F7.5*, *fbxa-59*, *C49C8.5* and *spp-18*) (Fig. 6, Supplementary Fig. 5). Thus, TCER-1 repressed the expression of both PMK-1-dependent as well as -independent genes induced by PA14 infection.

**Fig. 7:**
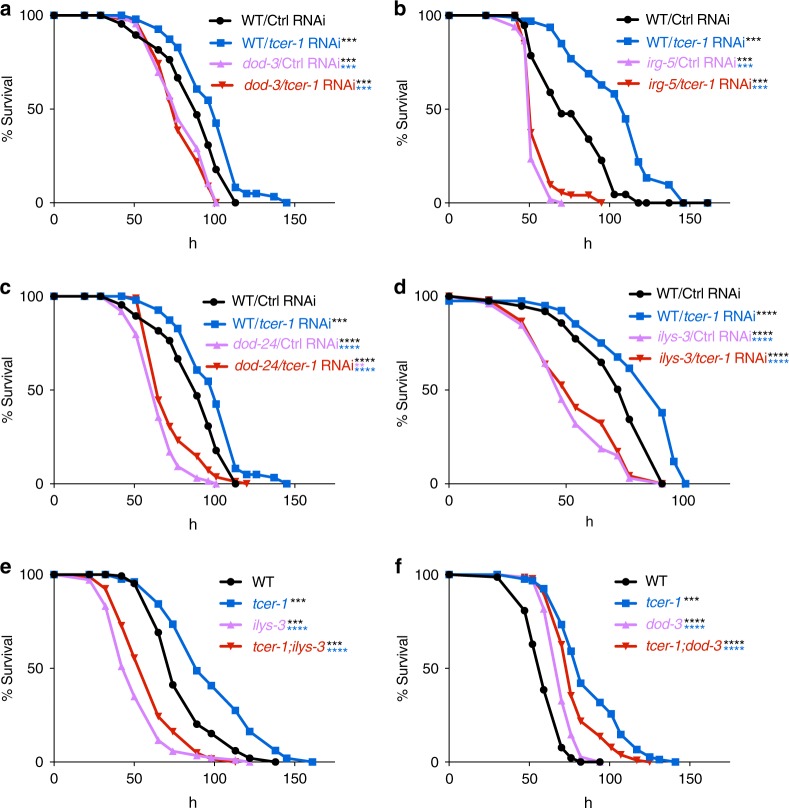
TCER-1-repressed genes are essential for resistance against PA14. Survival of wild-type L4 animals grown from egg stage on empty control vector (black, WT/Ctrl) or *tcer-1* RNAi bacteria (blue, WT/*tcer-1*) and then transferred to PA14 plates compared to the survival of **a** *dod-3*, **b** *irg-5*, **c** *dod-24* or **d** *ilys-3* mutants grown on empty control vector (pink curves) or *tcer-1* RNAi bacteria (red). **a** WT/Ctrl (*m* = 87.63 ± 2.2), WT/*tcer-1* (*m* = 99.27 ± 2.1), *dod-3*/Ctrl (*m* = 79.95 ± 1.5), *dod-3*/*tcer-1* (*m* = 78.8 ± 1.4). **b** WT/Ctrl (*m* = 77.67 ± 3.0), WT/*tcer-1* (*m* = 104.62 ± 2.7), *irg-5*/Ctrl (*m* = 53.07 ± 0.7), *irg-5/tcer-1* (*m* = 56.69 ± 1.2). **c** WT/Ctrl (*m* = 87.63 ± 2.2), WT/*tcer-1* (*m* = 99.27 ± 2.1), *dod-24*/Ctrl (*m* = 66.18 ± 1.4), *dod-24/tcer-1* (*m* = 74.20 ± 1.5). **d** WT/Ctrl (*m* = 72.12 ± 2.3), WT/*tcer-1* (*m* = 81.33 ± 2.5), *ilys-3*/Ctrl (*m* = 52.15 ± 1.9), *ilys-3/tcer-1* (*m* = 55.21 ± 2.0). **e** Survival of wild type and mutants transferred to PA14 plates at L4 stage. WT (black, *m* = 88.05 ± 2.0), *tcer-1* (blue, *m* = 111.81 ± 2.3), *ilys-3* (pink, *m* = 54.29 ± 1.3) and *tcer-1;ilys-3* (*m* = 60.74 ± 1.5). **f** WT (*m* = 60.06 ± 0.8), *tcer-1* (*m* = 87.9 ± 2.0), *dod-3* (*m* = 70.87 ± 0.75) and *tcer-1;dod-3* (*m* = 79.06 ± 1.3). Survival estimated using the Kaplan−Meier analysis and shown as mean lifespan in hours (*m*) ± standard error of the mean (SEM). *P* values were adjusted for multiplicity where applicable. Asterisks indicate statistical significance <0.01 (**), <0.001 (***) and <0.0001 (****) and their color denotes the strain of comparison. Assays in panels (**a**) and (**c**) were performed in the same biological replicate so their controls are shared. Details of number of animals and data from additional trials are presented in Supplementary Table 6

We next asked if either the PMK-1-dependent and/or -independent genes repressed by TCER-1 had any functional roles in immunity against PA14. Mutants available for four of the genes described above, *dod-3*, *dod-24*, *irg-5* and *ilys-3*, were tested for their resistance to PA14 upon *tcer-1* knockdown. Wild-type *C. elegans* subjected to *tcer-1* RNAi throughout their development and then exposed to PA14 at the L4 stage exhibited a significant increase in survival compared to control animals (Fig. 7a–d, Supplementary Table 6A). However, *dod-3, irg-5, dod-24*, and *ilys-3* mutants did not exhibit any lifespan extension under the same conditions (Fig. 7a–d, Supplementary Table 6A). *tcer-1;dod-3* and *tcer-1;ilys-3* double mutants were also significantly more susceptible to PA14 than *tcer-1* mutants alone, confirming the observations made through RNAi-knockdown of these genes (Fig. 7e, f, Supplementary Table 6B and 6C). Overall, these findings indicate that TCER-1 represses the expression of multiple genes critical for conferring resistance against PA14 infection and these include genes operating in the PMK-1 pathway as well as potentially novel immunity effectors.

### *tcer-1* mutants exhibit improved healthspan parameters

**Fig. 8:**
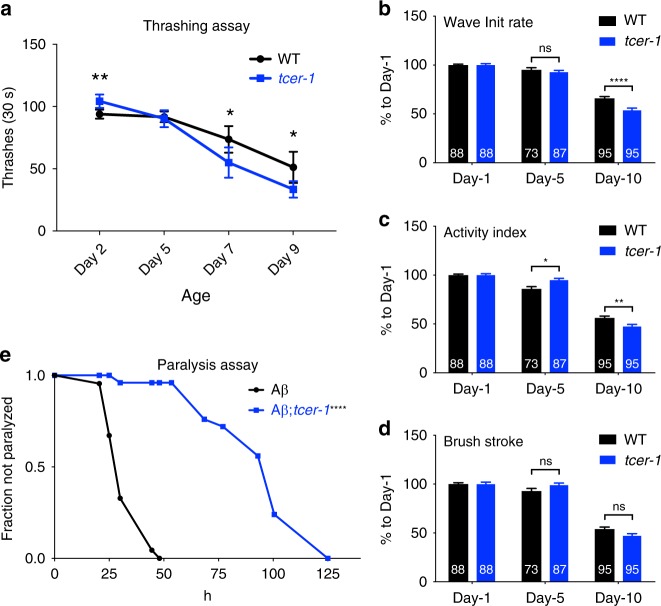
Young *tcer-1* mutants exhibit improvement in some healthspan parameters. **a** Thrashing efficiency compared between wild-type (WT, black) and *tcer-1* mutants (blue) at different ages. Data shown are combined from six independent biological replicates with 20 animals tested per strain, per replicate. Statistical significance was calculated using an unpaired two-tailed *t* test. **b**–**d** Measurement of change in swimming parameters with age in wild-type *C. elegans* (WT, black) and *tcer-1* mutants (blue) quantified using CeleST^55^. In this longitudinal assay, the differences seen between the strains on day 1 of adulthood were not replicated but a clear reduction in day 10 *tcer-1* mutants’ wave initiation rate (**b**, number of body waves initiated per minute) and activity index (**c**, measure of overall swimming activity) was observed. Brush stroke (**d**, depth of movement) was also reduced in the mutants but not in a statistically significant manner. Data combined from three independent biological replicates with number of animals tested for each genotype indicated on the bars. Statistical significance was calculated using two-way Anova, error bars represent standard error of the mean. **e** Loss of *tcer-1* delays paralysis in Aβ proteotoxicity model. Onset of paralysis measured in the amyloid β proteotoxicity model strain GMC101 (Aβ, black) and Aβ strain carrying a *tcer-1* mutation (Aβ*;tcer-1*, blue). Kaplan−Meier analysis for mean paralysis time: Aβ *m* = 33.1 h, *n* = 67; Aβ;*tcer-1* *m* = 95 h, *n* = 25. Asterisks indicate statistical significance <0.05 (*), <0.01 (**), and <0.0001 (****), ns not significant. Data from additional trials in Supplementary Fig. 9

Immunoresistance is a prominent hallmark of improved healthspan^13–15^. Based on the stress-resistance phenotypes of *tcer-1* mutants, we asked if they showed improvement in other aspects of healthspan too. We focused on mobility, a key function that undergoes age-related decline both in *C. elegans* and humans. Using a standard “thrashing” assay, we found that young (day 2) *tcer-1* mutants performed significantly better than their wild-type counterparts (Fig. 8a). Since *tcer-1* mutants exhibited increased immunoresistance during the reproductive phase but at post-reproductive ages became more sensitive (Fig. 3a), we tested if this was true for thrashing efficiency too. Indeed, by day 5, there was no difference in thrashing capacity of *tcer-1* mutants and wild type, whereas post-reproductive days 7 and 9 mutants showed significantly impaired thrashing as compared to wild type (Fig. 8a). Interestingly, when we examined several individual parameters of swimming locomotion, using the software CeleST that tracks age-related decline in mobility and muscle function^55^, young mutants did not show a consistent or significant improvement (Fig. 8b–d). As seen with thrashing capacity though, post-reproductive (day 10) mutants showed marked decrease in some swimming activity. We tested the impact of *tcer-1* mutation on another healthspan feature, the ability to combat proteotoxic degenerative disease, using a *C. elegans* model of amyloid β(Aβ) proteotoxicity. The strain GMC101 expresses human Aβ_1−42_ constitutively in muscle cells and, when transferred from 20 to 25 °C, animals exhibit full body paralysis^56^. We found that *tcer-1* mutants exhibited a striking delay in developing paralysis. In one trial, while 50% of animals of the control strain were fully paralyzed by 33 h, and 100% by 50 h, the *A*β*;tcer-1* strain did not show onset of paralysis till 30 h, and 50% and 100% of the population were only paralyzed by 95 and 125 h, respectively (Fig. 8e, Supplementary Fig. 9). Thus, *tcer-1* mutation dramatically improved the ability of *C. elegans* to combat proteotoxicity and aggregation-related pathology. Together, the mobility and paralysis data suggest that TCER-1 may have a widespread impact on stress response and a nuanced influence on individual healthspan features, raising possibilities of molecularly separating specific aspects of age-related debilitation.

## Discussion

The innate immune system is an ancient and conserved system of defense against pathogens, and MAPK signaling, through PMK-1, has a well-established role in driving transcriptional changes that mediate immunity^21^. Our data suggest that TCER-1 may counter such changes directly or indirectly. PMK-1 was predicted to be a TCER-1-repressed gene and confirmed by both molecular and transgenic approaches. Several known PMK-1 targets were also upregulated in *tcer-1* mutants. Yet, the *pmk-1* null mutation did not completely abrogate *tcer-1* mutants’ resistance suggesting that TCER-1-mediated immunity suppression is only partially through PMK-1-repression. What other processes may be impacted by TCER-1 to impair immunity? Several cellular surveillance pathways are activated by pathogen exposure, either partially or completely independent of PMK-1^22–26^. In light of this, and TCER-1’s role in inhibiting multiple stress modalities, a logical prospect is that cellular stress response factors may be inhibited by TCER-1. Indeed, several genes with roles in UPR^mt^ (e.g., *mrps-5*)^57^ and HR (e.g., *cysl-1*)^58^ were included in the TCER-1 DOWN^37^ group. *tcer-1* mutants showed higher expression of the central UPR^mt^ regulator, *atfs-1*^22,23^, *hsp-6*, the UPR^mt^-specific chaperone^59^ (Supplementary Fig. 6a, b) and *hif-1*^26^, the key HR mediator (Supplementary Fig. 6c). Accordingly, we also noted a significant overlap between the TCER-1 DOWN^37^ genes and ATFS-1-regulated genes (Supplementary Fig. 6d–f)^22,23^.

Sequence-based computational approaches have led to the identification of numerous immune effectors in *C. elegans*, including conserved and invertebrate-specific lysozymes (LYS and ILYS proteins, respectively) that may digest bacterial cell walls^60,61^. TCER-1-repressed genes comprised some of these factors as well as novel proteins with predicted antibacterial functions. For instance, *C50F7.5*, a TCER-1-repressed gene upregulated >50-fold upon infection (Fig. 6c), encodes a protein that shares ~60% sequence similarity with a cell-surface glycoprotein in *Clostridium thermocellum*. Cell-surface glycoproteins function in pathogen recognition, the most well known being the family of Toll-like receptors— receptors that sense pathogen associated molecular patterns expressed by infective agents^62^. Notably, no pathogen-specific receptors have been identified yet in nematodes. Similarly, the TCER-1-repressed gene, *fbxa-59*, which encodes an F-Box protein, is highly upregulated upon PA14 exposure (Fig. 6b). F-Box proteins are E3 ligase components that mediate proteasomal protein degradation and influence longevity^63^. Natural allelic variations of the HECT-domain E3 ligase, HECW-1, have been implicated in PA14 avoidance^64^, and the CUL-6 E3 ligase complex is involved in the response to *Nematocida parisii*^65^. But, E3 ligases activated by *Pseudomonas* infection remain unknown. Characterization of the TCER-1-repressed transcriptome can reveal novel insights into the molecular repertoire of nematode immunity.

Cell non-autonomous mechanisms that govern longevity and stress resistance have been demonstrated recently in many contexts. In *C. elegans*, protein-folding imbalance in muscles induces transcellular chaperone signaling that evokes stress responses in intestine and neurons^66^. XBP-1 and retrograde Wnt signaling act in neurons to coordinate the organismal UPR^mt^ and UPR^ER^ responses (reviewed in refs. ^67,68^), whereas intestinal DAF-16 expression is sufficient to confer longevity in germline-less mutants^69^. Similarly, dFOXO activity in *Drosophila* fat body regulates brain insulin signaling as well as lifespan^70^, whereas Activin disruption in muscles impacts systemic insulin metabolism^71^. In mice, Xbp1s activity in Pomc neurons is sufficient to improve hepatic glucose metabolism and protect against diet-induced obesity^72^. An interesting finding of this study is that TCER-1 can function in any of the four somatic tissues we tested to impact longevity and stress response. Expressing TCER-1 in any somatic tissue of *tcer-1* mutants suppressed their PA14 resistance. While it is possible that this is simply a consequence of toxicity caused by TCER-1 overexpression, the fact that these transgenes (a) do not cause lifespan shortening in normal animals fed either OP50 (Supplementary Data 1) or PA14 (Supplementary Table 4C), (**b**) do not shorten *glp-1* mutants longevity on OP50 (Supplementary Data 1) and (**c**) extend the lifespan of *tcer-1;glp-1* mutants on OP50, supports TCER-1’s non-autonomous mode of action in its anti-immunity and prolongevity functions. It is, indeed, intriguing that TCER-1 overexpression has profoundly different consequences for the animal depending on consumption of benign food or noxious pathogen. It implies that TCER-1’s presence in any somatic tissue can convert it into a coordinating center for orchestrating an animal-wide outcome, and that it may contextually direct the release of vastly different signals from the same tissue. Such molecular versatility can be highly beneficial to an animal in the wild facing rapidly fluctuating conditions to coordinate different aspects of its physiology.

TCER-1 is a longevity-promoting factor because it is essential for the lifespan extension conferred by germline loss, and because its overexpression in normal, fertile adults increases their lifespan^35^. Hence, the widespread, enhanced stress resistance of *tcer-1* mutants was surprising considering the strong correlation between longevity and stress tolerance. It suggested that mechanisms that confer stress resistance do not directly confer longevity, although we cannot rule out this possibility for an as-yet untested stress modality. Though infrequently, mutations that increase thermal or oxidative stress resistance but do not increase lifespan (e.g., *pep-2*)^10^ as well as ones that enhance lifespan without improving stress endurance (e.g., *cep-1*)^11^ have been described. Germline-less *daf-16* mutants are shorter lived than wild type but exhibit greater thermotolerance^69^, and *daf-2* mutants subjected to RNAi inactivation of the prolongevity gene, *smk-1*, continue to exhibit increased thermotolerance^73^. But, it is noteworthy that the instances of uncoupling reported so far have been exceptions and prolongevity genes largely act as pro-stress-resistance factors. The thermotolerance uncoupling notwithstanding, *daf-16* and *smk-1* confer resistance against numerous stressors in long-lived and wild-type animals^1^. Importantly, the knockdown of neither gene improves stress resistance, whereas *tcer-1* inactivation does, widely and consistently. *tcer-1* not only uncouples longevity from multiple stress modalities, it broadly antagonizes stress endurance. Additionally, while our previous study demonstrated that DAF-16 and TCER-1 collaborated to establish lipid homeostasis and promote longevity in germline-less adults^37^, these data reveal that, for stress resistance, TCER-1 and DAF-16 have an antagonistic relationship. They highlight the complexity of the links between lifespan and stress biology and suggest that the relationship is context dependent and plastic.

In most species, infections reduce fertility, whereas increased reproduction is accompanied by immunosuppression^16^. Alterations to a woman’s immune system are critical during pregnancy to tolerate fetal tissue, but are associated with increased susceptibility to infectious agents^46^. In insects, infections reduce fecundity while mating diminishes infection resistance^16^. In *C. elegans* too, sterile mutants have been reported to exhibit increased pathogen resistance^40^. This study, however, indicated that embryonic development, rather than absence of gametes per se, repressed immunity. It is also salient that while fertility and immunity appear to be mutually antagonistic, both diminish with age. In *C. elegans*, PMK-1 activity declines with age and thought to underlie immunosenescence^45^. Since TCER-1 acts, in part, to repress PMK-1, it is possible that the age-related loss of *tcer-1* mutants’ PA14 resistance is linked to this reduction. Interestingly, the immune resistance of some sterile mutants is also reduced at post-reproductive ages^40^, emphasizing that the association of immunity and fertility is multifaceted and governed by factors such as age and resource allocation. The fact that TCER-1 is essential for reproductive health and exerts a repressive influence on immunity only during the reproductive phase, and the observation that raising its levels allows the animal to escape some of the fertility loss inflicted by infection, suggest that TCER-1’s primary molecular function may be to promote fertility. Indeed, we found that both somatic and germline levels of TCER-1 are highest in young animals and decline with age. Interestingly, human oocytes also express high levels of TCERG1 mRNA and its levels decline in older oocytes^74^, so it may have a conserved role in promoting reproductive fitness. Identification of both PMK-1-dependent and -independent TCER-1 immunity functions also implies that multiple pathways may link immunity and fertility. TCER-1 can be a useful handle to decipher the mechanistic differences between these vital processes.

## Methods

### *C. elegans* strains and culture

All strains were grown and maintained on standard nematode growth medium (NGM) at 20 °C using *E. coli* strain OP50 as the food source. For experiments involving RNAi, NGM plates supplemented with 1 ml per liter of 1 M IPTG (Isopropyl *β*-d-1-thiogalactopyranoside) and 1 ml per liter of 100 mg per ml ampicillin. The strains used in this study include N2 (wild type), CF2166 [*tcer-1*(*tm1452*) II], CF1038 [*daf-16*(*mu86*) I], CF1903 [*glp-1*(*e2144*) III], CF2154 [*tcer-1*(*tm1452*) II; *glp-1*(*e2144*) III], CF1880 [*daf-16*(*mu86*) I; *glp-1*(*e2144*) III], CF2858 [*tcer-1*(*tm1452*)*; Ptcer-1::TCER-1::GFP, Podr-1::RFP*] AGP214 [*daf-16*(*mu86*) I; *tcer-1*(*tm1452*) II], AGP215 [*daf-16*(*mu86*) I; *tcer-1*(*tm1452*) II; *glp-1*(*e2144*) III], AGP1 {*glmIs* [*Ptcer-1::tcer-1::GFP+Podr-1::RFP*]} (obtained by integrating the transgene in CF2032 strain^35^), IT213 [*tcer-1 prom: tcer-1ORF:gfp:tcer-1 3*′*utr*]^47^, ZD1195 {*qdEx101*[*Poperon::islo-1::pmk-3::pmk-2::GFP::pmk-1::mCherry*]}^53^, PRJ112 {*mutEx70* [*pmk-1::GFP+rol-6(su1006*)]}^52^, AGP97 [*pmk-1*(*km25*) IV]^75^ (obtained by outcrossing to Ghazi lab N2), AGP213 [*tcer-1*(*tm1452*) II; *pmk-1*(*km25*) IV], RB2356 [*dod-3*(*ok3202*) V], RB2478 [*irg-5*(*ok3418*) V], RB1994 [*dod-24*(*ok2629*) IV], VC2496 [*ilys-3*(*ok3222*) *V*], AGP256 [*tcer-1*(*tm1452*) II; *ilys-3*(*ok3222*) *V*], AGP258 [RB2356 {*dod-3*(*ok3202*) *V*} outcrossed to Ghazi lab N2], AGP257 [*tcer-1*(*t*′*m1452*) *II; dod-3*(*ok3202*)*V*], GMC101{*dvIs100* (*unc-54p::A*β*-1-42::unc-54 3*′*-UTR+mtl-2p::GFP*)} and AGP275 [*tcer-1(tm1452) II; dvIs100 (unc-54p::A*β*-1-42::unc-54 3*′*-UTR+mtl-2p::GFP*)]. Transgenic strains expressing TCER-1 under control of tissue-specific promoters in different genetic backgrounds generated for this study are listed in Supplementary Table 7.

### Transgenic strain generation

To generate the *Ptcer-1::tcer-1::gfp* construct, 5.6 kb region of *tcer-1* gene (4.0 kb comprising the coding region covering all *tcer-1* transcripts and 1.5 kb sequence upstream of the first *tcer-1* exon) was amplified with primers modified to introduce *Sph*I and *Xma*I restriction sites (forward 5′ gctagGCATGCgcaagtatttgagcactac3′; reverse 5′ taagcaCCCGGGTCttgctttctgcgatcccgc 3′). The amplified product was cloned into the GFP expression vector pPD95.77 (Addgene plasmid 1495). The full length *tcer-1* fragment was inserted upstream of, and in frame with, GFP at the *Sph*I site (pAG10). To generate tissue-specific TCER-1 expressing constructs the 4 kb *tcer-1* coding region was inserted into plasmids created previously in the lab for another gene, *nhr-49* (Ratnappan and Ghazi, unpublished). The *tcer-1* coding region was amplified with primers modified to introduce *Sal*I and *Acc*65I restriction sites (forward 5′gctagGGTCGACatgagccacgaaaatc3′; reverse 5′taagcaGGTACCTCttgctttctgcgatcccgc3′). *nhr-49* coding region was removed from the tissue-specific promoter plasmids using restriction enzymes, *Sal*I and *Acc*65I, and replaced with PCR amplified and digested *tcer-1* coding region by ligation into the respective plasmids in frame with the GFP. The plasmids thus obtained were pAG11 (a 2.4 kb muscle promoter^76^, *Pmyo-3::TCER-1::GFP*), pAG12 (1.9 kb intestinal promoter^77^, *Pgly-19:: TCER-1::GFP*), pAG13 (400 bp hypodermal promoter^78^, *Pcol-12:: TCER-1::GFP*), and pAG14 (4.2 kb region neuronal promoter^79^, *Prgef-1:: TCER-1::GFP*). Transgenic strains were generated by injecting the plasmids at a concentration of 25 ng per μl or 100 ng per μl along with 3.75 ng per μl or 15 ng per μl of *Pmyo-2::mCherry* co-injection marker, respectively. Three to six independent stable transgenic lines were generated for each of the genetic backgrounds in which the transgene was injected. Transgenic strains were maintained by picking fluorescent animals in each generation.

### Lifespan assays

All lifespan experiments were conducted at 20 °C on *E. coli* OP50 plates unless otherwise noted. Between 20 and 30 L4 hermaphrodites were transferred to each of ~5−6 plates per experiment and observed at 24−48 h intervals to document live, dead or censored (animals that exploded, bagged or could not be located) animals. Animals were scored as dead when they failed to respond to gentle prodding with a platinum wire pick. Fertile strains were transferred every other day to fresh plates until progeny production ceased. For lifespan assays of strains with the temperature sensitive *glp-1* mutation, eggs were picked and maintained at 20 °C for 2−4 h, transferred to 25 °C to induce sterility and then returned to 20 °C on day 1 of adulthood (72 h later) for lifespan analysis. For performing lifespan assays of transgenic strains with extrachromosomal arrays, eggs were picked onto fresh OP50 plates, incubated at the appropriate temperature and 48 h later L4 animals were screened under a Leica M165FC microscope with a fluorescence attachment (Leica Microsystems, Wetzlar, Germany) for animals carrying the red coinjection marker labeling pharyngeal muscles. At the same time, a similar number of age-matched, nontransgenic siblings were collected for each strain and assayed for lifespan as internal controls in the experiment. Each lifespan was tested at least twice and often in 3−5 biological replicates. All survival data were plotted via the Kaplan–Meier method. Statistics were calculated using the nonparametric log-rank Mantel−Cox method using OASIS2 (https://sbi.postech.ac.kr/oasis2/)^80^ and subjected to multiplicity correction in experiments that involved more than two strains/conditions.

### Pathogenic stress assays

Pathogenic bacterial strains used in this study include *Pseudomonas aeruginosa* (strains PA14 and PA01) and *Staphylococcus aureus* (NCTC8325). These strains were streaked from frozen stocks onto Luria Bertani (LB) agar (PA14 and PA01) or Brain Heart Infusion (BHI) agar (NCTC8325) plates, incubated at 37 °C overnight and stored at 4 °C for a week or less. For studies with PA14 and PA01, single colonies from the streaked plates were inoculated and grown in King’s broth overnight at 37 °C with shaking. ~20 µl of this broth culture was seeded onto slow killing (SK) plates (modified NGM plates containing 0.35% peptone instead of 0.25%) and incubated for 24 h at 37 °C. The plates were then left to sit at room temperature (RT) for 24 h prior to use. Between 20 and 30 L4 hermaphrodites per strain were transferred to each of ~5−6 OP50 plates per experiment, incubated at 25 °C and monitored at 6−12 h intervals to account for live, dead or censored animals as described above. Using a variation of this paradigm, PA01 seeded onto standard NGM plates was also used for pathogen sensitivity assays. For studies with *S. aureus* (NCTC8352), single colonies from the streaked plate were inoculated and grown in BHI broth overnight at 37 °C with shaking and then ~10 µl of this was spread onto BHI-agar plates. Plates were incubated overnight at 37 °C for 24 h followed by storage at RT for 24 h. L4-stage animals were transferred to pathogenic plates, maintained at 25 °C and monitored for survival every 24 h.

To analyze PA14 sensitivity of *C. elegans* strains at various stages of life, temporal assays were conducted by picking eggs of wild-type and *tcer-1* strains on OP50 plates, growing at 20 °C and then transferring them onto PA14 SK plates at L4, day 2, day 4, day 6 or day 9 of adulthood and monitoring for survival at 25 °C as mentioned above. Reproductively active animals were transferred to fresh OP50 plates every day till the relevant day of PA14 exposure. To rule out the impact of internal hatching on experimental outcomes, wild-type and *tcer-1* L4 larval stage animals were treated with 100 μg per ml of FUDR on NGM plates with OP50. Exposing *C. elegans* to this treatment for 24 h at 15 °C before transferring to PA14 SK plates prevented the eggs from hatching. For RNAi experiments, animals were grown to the L4 stage on standard RNAi plates seeded with *E. coli* HT115 carrying an empty vector control (pAD12) or the relevant RNAi clone before transferring to PA14-seeded SK plates and assaying for survival at 25 °C. Kaplan−Meier analysis and statistics were performed as described above for lifespan assays.

### Thermal stress assay

Age-matched day 2 adults of different strains were transferred onto OP50 plates. The plates were sealed using parafilm, put into Ziploc bags and submerged in water bath maintained at 35 °C for 6 h. The plates were then recovered, unsealed and kept at 20 °C for 12 h before scoring for live, dead or censored animals. The percentage of live animals between strains were compared for statistical significance using an unpaired *t* test.

### Oxidative stress assay

Age-matched L4-stage animals of different strains were transferred to NGM plates containing 7.0 mM *t*-butyl hydrogen peroxide (tBOOH) (Sigma) seeded with OP50. These plates were incubated at 20 °C and the animals were monitored every 6−12 h intervals and scored for survival^13^.

### ER stress assay

NGM plates with a final concentration of 2 µM Tunicamycin (MP Biomedicals) dissolved in Dimethyl Sulfoxide (DMSO) were prepared ensuring the final concentration of DMSO does not exceed 0.2%^81^. The plates were seeded with OP50 for 24 h and age-matched L4 animals of different strains transferred to them and incubated at 20 °C. Survival was monitored daily as described above for lifespan assays.

### Ionizing radiation (IR) stress assay

Age-matched, L4 stage hermaphrodites were plated on each of four 6-cm plates with 30−100 animals per plate depending on genotype and IR dose. The following day, adults were exposed to 0, 10, 50, or 100 Gy of IR from a ^137^Cs source (Gammacell^®^1000 Elite, Nordion International Inc.). Twelve hours post-irradiation, the animals were plated (two animals per 3-cm dish) and allowed to lay eggs for 12 h. The number of eggs laid was counted and quantified.

### Fertility assessment on OP50 and PA14

Gravid day 2 animals were allowed to lay eggs for a 2 h period on OP50 plates. The eggs were allowed to hatch and develop at 20 °C for 65 h till they are about to start laying eggs of their own. At this point the animals were transferred to single plates (ten plates per strain per experiment) and incubated at 20 °C. At the 4 h timepoint the animals were transferred to fresh plates, moved back to 20 °C and eggs laid on the older plate counted. This was repeated at the 8, 12, 24 and 48 h timepoints. To calculate percent reduction in egg laying upon pathogen stress, the number of eggs laid by each strain on PA14 was normalized to its OP50 control at the same timepoint. The total brood size of each strain was calculated as the average of the total number of eggs laid per animal per strain during its lifetime. Unpaired *t* test was used to calculate statistical differences in egg laying between strain/conditions.

### Q-PCRs

RNA was isolated from L4-stage strains exposed to PA14 or maintained on OP50 for 8 h. Animals were washed off plates followed by 3× washes with M9 to remove residual bacteria. Trizol was added to the animal pellet and the tubes were vortexed for 30 s and flash frozen in liquid nitrogen. Following this the samples were allowed to thaw at 37 °C and then subjected to ~7 cycles of freeze-thaw between liquid nitrogen and a 37 °C water bath. RNA was then isolated using the standard phenol-chloroform extraction followed by ethanol precipitation and measured for quality and quantity using a nanodrop. RNA was treated with DNase I (Sigma-Aldrich St. Louis, MO) and cDNA was prepared from 1 mg of total RNA in a 20 µl reaction using the High Capacity RNA-to-cDNA kit (Applied Biosystems, USA). Q-PCRs were performed using an ABI 7000 machine (Applied Biosystems, USA). PCR reactions were undertaken in 96-well optical reaction plates (ABI PRISM N8010560). A 20 µl PCR reaction was set up in each well with 10 µl SYBR Select Master Mix Kit (Applied Biosystems, USA), 50−100 ng of the converted cDNA and 0.25 M primers. For every gene at least three independent biological samples were tested, each with three technical replicates. Primers used in this study are listed in Supplementary Table 8.

### Microscopy and fluorescence imaging

For imaging age-related change in TCER-1::GFP levels, the IT213 strain was grown at 20 °C on OP50 till day 1 or 4 of adulthood before dissection. For documenting PA14-induced changes, L4 larvae grown on OP50 at 20 °C were transferred to PA14 or OP50 plates and kept at 25 °C for 12−14 h before dissections. Dissections were performed in 3.5 µl 1× sperm salts (50 mM PIPES, pH 7.0, 25 mM KCl, 1 mM MgSO_4_, 45 mM NaCl, 2 mM CaCl_2_) with ~0.2 µl 10 mM Levamisole, one or two animals at a time. Both gonads and intestines were imaged within 3 min of dissection. Images were acquired on a Nikon A1r confocal microscope as 0.5 µm Z-stacks with identical imaging parameters for all samples. Image analysis was performed using Velocity 3-D imaging software by hand-selecting the region of interest (nuclei) (using both GFP and transmitted light where necessary) to identify the nuclei of interest and average pixel density per nucleus was measured. To account for potential differences in nuclear sizes skewing the results, we also calculated total pixel intensity of each nucleus/volume for the nucleus and compared these values between the different ages and treatments. Similar results were obtained (Supplementary Fig. 8). Only nuclei where GFP signal was visible were used for the quantification. By day 4, many intestinal nuclei lost all GFP expression and these were not included in the quantification as it was often difficult to distinguish the nuclear border in these animals. Hence, the intestinal downregulation of TCER-1::GFP by day 4 is more dramatic than indicated by the bar graphs in Fig. 4g and Supplementary Fig. 8.

For imaging PMK-1::GFP and PMK-1::mCherry strains’ fluorescence, microscopy was performed with a Leica M165/DCF fluorescence microscopes equipped with a Retiga 2000 digital camera or a Leica DM5500B compound scope with Leica imaging software. Before imaging, PMK-1::GFP and PMK-1::mCherry strains were grown from egg to L4 stage on RNAi plates as described above. Ten to fifteen animals exposed to PA14 for 24 h were immobilized in ~3 µl of 10 mM sodium azide and imaged at ×20 and ×40 magnification (focusing on head and mid-body). Images were acquired using the LAS X software (Leica) and quantified for fluorescence intensities using Fuji (ImageJ) software. Averaged intensities per genotype or condition were plotted with their respective standard errors. Unpaired two-tailed *t* tests were used to determine statistical significance in Prism software.

### Bacterial colony-forming unit (CFU) analysis

Age-matched L4 stage animals of each strain (six replicates of ten nematodes per strain) were infected with PA14 for 24 h at 25 °C. Post-infection animals were transferred to M9 solution containing 25 mM levamisole in order to paralyze nematodes and inhibit pharyngeal pumping. To eliminate *P. aeruginosa* stuck to the body wall, the nematodes were transferred to a NGM plate containing ampicillin (1 mg per ml) and gentamicin (1 mg per ml) for 15 min. To further ensure elimination of any residual *P. aeruginosa*, the animals were then transferred to a new NGM plate containing ampicillin (1 mg per ml) and gentamicin (1 mg per ml) for 30 min. Finally, the nematodes were lysed and the lysates were serially diluted in M9. Following serial dilution, the lysates were plated onto Luria-Bertani plates containing Rifampicin (100 µg per ml). The plates were then incubated overnight at 37 °C and colonies were counted to determine CFUs^82^.

### Bacterial colonization assay

To assess bacterial load in the intestinal lumen, animals were transferred from OP50 plates to ones containing GFP-labeled PA14 bacteria for 24 h and maintained at 25 °C. GFP intensity in the pharynx was visualized as described above and quantified using the Fuji (ImageJ) software. Briefly, the corrected total cell fluorescence (CTCF) values for all the strains/conditions were measured. The average CTCF value obtained for WT animals fed GFP-OP50 was then used as the reference to which all the other conditions were compared.

### CeleST swimming assay

Both wild-type and the *tcer-1* strains were maintained at 15 °C on OP50 bacteria seeded on NGM plates. L4 animals were picked for age synchronization and transferred to 20 °C for the assay. Swimming was recorded under dark field by a Lumenera camera (Lt225M) installed on a trinocular dissecting microscope, and the StreamPix 7 software was used for camera control and video recording (18 frames per second for a total of 30 s in each video). In each measurement, four crawling animals were randomly picked and transferred into 50 µl M9 solution contained in a 1 cm circle imprinted on a microscope slide. Swimming parameters, including wave initiation rate, brush stroke and activity index, were measured using CeleST software^55^.

### Thrashing assay

To measure thrashing rate with age, L4-stage larvae were picked and maintained at 20 °C. On day 2, adults were transferred to an unseeded NGM plate (to remove excess OP50 from the body of the animal), then transferred one at a time into 1 ml of M9 media in an unseeded 3 cm plate. The animals were allowed to acclimate for 5 min in the liquid, then the number of body bends counted for a 30-s interval. A body bend constituted the movement of the head and/or tail beyond the midline of the body. Thrashing rate was measured similarly on animals aged to 5, 7, and 9 days of adulthood at 20 °C.

### Paralysis assay

L4-staged animals of strains maintained at 20 °C were picked and shifted to 25 °C. Plates were scored after 24 h and then at 24 h intervals till the entire population was paralyzed. An animal was scored as paralyzed if it did not move upon prodding with a pick.

### Statistical analyses

All data in this article are expressed as mean (*m*) ± standard error of mean (SEM) unless otherwise noted. Graphs were plotted and statistical analyses performed using Prism, OASIS2 or Microsoft Excel. Probability levels of 0.05 or below were considered statistically significant. Statistical significance of overlap between two groups of genes was calculated on Nemates.org (http://www.nemates.org/MA/progs/overlap_stats.html). The probability of overlapping genes was calculated using the hypergeometric probability formula and the representation factor (RF) was calculated as the number of overlapping genes divided by the expected number of overlapping genes drawn from two independent groups.

### Reporting summary

Further information on research design is available in the Nature Research Reporting Summary linked to this article.

## Supplementary information

Supplementary Information

Peer Review File

Description of Additional Supplementary Files

Supplementary Data 1

Reporting Summary

Source Data

## Data Availability

The authors declare that all data generated or analyzed supporting the findings of this study are available within the paper and its supplementary information files. All data are available from the corresponding author upon request. The raw data for Figs. 3c–e, 4g–j, 5, 6 and 8b–d are included in the Source Data file.
