# Supplementary material for: The longevity-promoting factor, TCER-1, widely represses stress resistance and innate immunity
Source: Nat Commun. 2019 Jul 17;10:3042. doi: 10.1038/s41467-019-10759-z (PMC6637209; doi:10.1038/s41467-019-10759-z)
Supplement: Supplementary file 1 — Supplementary Information [file 41467_2019_10759_MOESM1_ESM.pdf]

Supplementary Information

The Longevity-Promoting Factor, TCER-1, Widely Represses Stress Resistance and Innate Immunity

Amrit et al.

Supplementary Figure 1: Resistance of *tcer-1* mutants to different stress paradigms and impact of *daf-16* mutation.

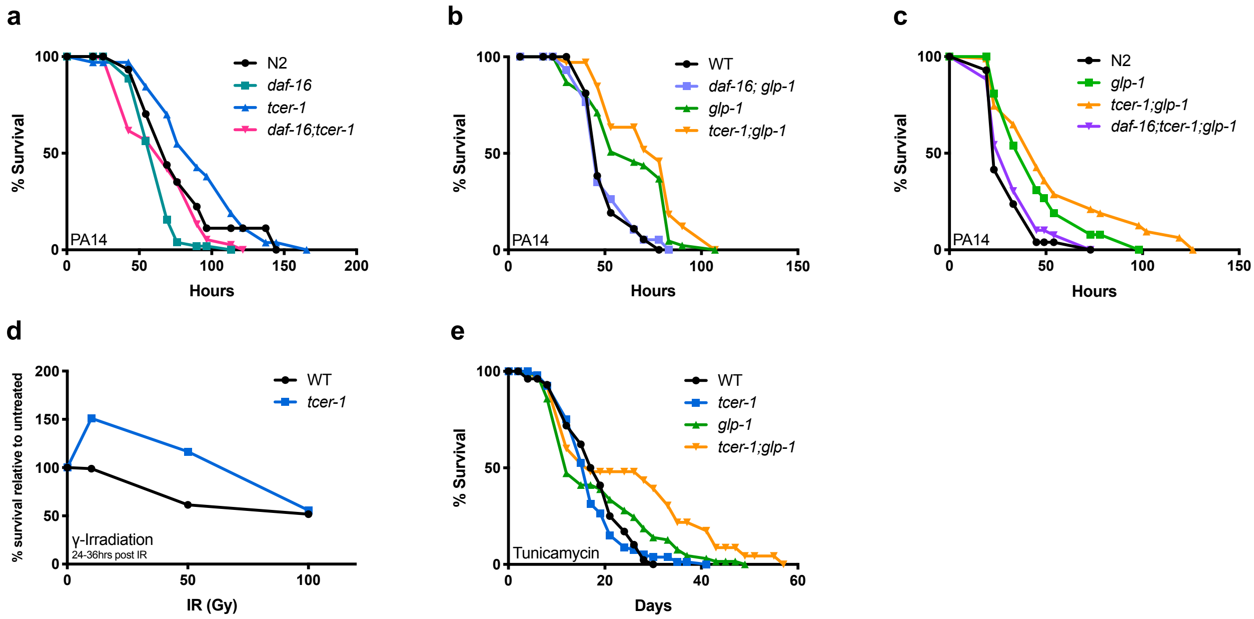

Survival of L4 stage wild-type animals (WT, black), *tcer-1* (blue), *glp-1* (green), *tcer-1;glp-1* (orange), *daf-16* (turquoise), *daf-16;glp-1* (indigo), *daf-16;tcer-1;glp-1* (purple) mutants exposed to different stressors. **(a-c)** Exposure to *P. aeruginosa* PA14. **(a)** WT (m = 78.67 ± 4.22, n = 45/100), *tcer-1* (m = 91.77 ± 4.26, n = 45/100, P vs WT <0.02), *daf-16* (m = 63.37 ± 1.63, n = 58/100, P vs WT <0.0003, P vs *tcer-1* <0.0001) and *daf-16;tcer-1* (m = 67.59 ± 3.11, n = 45/100, P vs *tcer-1* <0.001). **(b)** WT (m = 50.84 ± 1.35, n = 59/75), *glp-1* (m = 62.90 ± 2.55, n = 56/76, P vs WT <0.0001), *tcer-1;glp-1* (m = 72.08 ± 3.10, n = 38/76, P vs *glp-1* <0.05), *daf-16;glp-1* (m = 50.73 ± 2.22, n = 32/75, P vs WT <0.87, P vs *glp-1* <0.01, P vs *tcer-1;glp-1* <0.0001). **(c)** WT (m 30.83 = ± 1.87, n = 45/57), *glp-1* (m = 45.67 ± 2.3, n = 79/99, P vs WT <0.0001), *tcer-1;glp-1* (m = 54.54 ± 3.93, n = 62/100, P vs WT <0.0001, P vs *glp-1* <0.09), *daf-16;glp-1* (m = 40.39 ± 2.36, n = 85/104, P vs WT <0.001, P vs *glp-1* <0.06, P vs *tcer-1;glp-1* <0.001), *daf-16;tcer-1;glp-1* (m = 33.98 ± 1.97, n = 56/77, P vs N2 <0.3, P vs *glp-1* <0.0001, P vs *tcer-1;glp-1* <0.0001) **(d)** DNA damage. Viability of eggs laid by Day 1 hermaphrodites 24h after exposure to -irradiation. **(e)** ER Stress. Survival of L4-stage animals in the presence of 2μM Tunicamycin. WT (m = 18.13 ± 0.64, n = 91/104), *tcer-1* (m = 17.27 ± 0.67, n = 87/104, P vs WT <0.2), *glp-1* (m = 18.81 ± 1.03, n = 96/108, P vs WT <0.1) and *tcer-1;glp-1* (m = 24.91 days ± 2.96, n = 100/101, P vs WT <0.001, P vs *glp-1* <0.01). In (a-c and e), survival data shown as mean lifespan in hours (m) ± standard error of the mean (SEM). 'n' refers to number of animals analyzed divided by the total number tested in the experiment (some animals were censored from the analysis as described in the methods section). Data from additional trials for panels a-c are in Supplementary Table 1B, and for panel e in Supplementary Table 3B.

Supplementary Figure 2: Increased PA14-resistance of *tcer-1* mutants is not due to aberrant feeding or matricide.

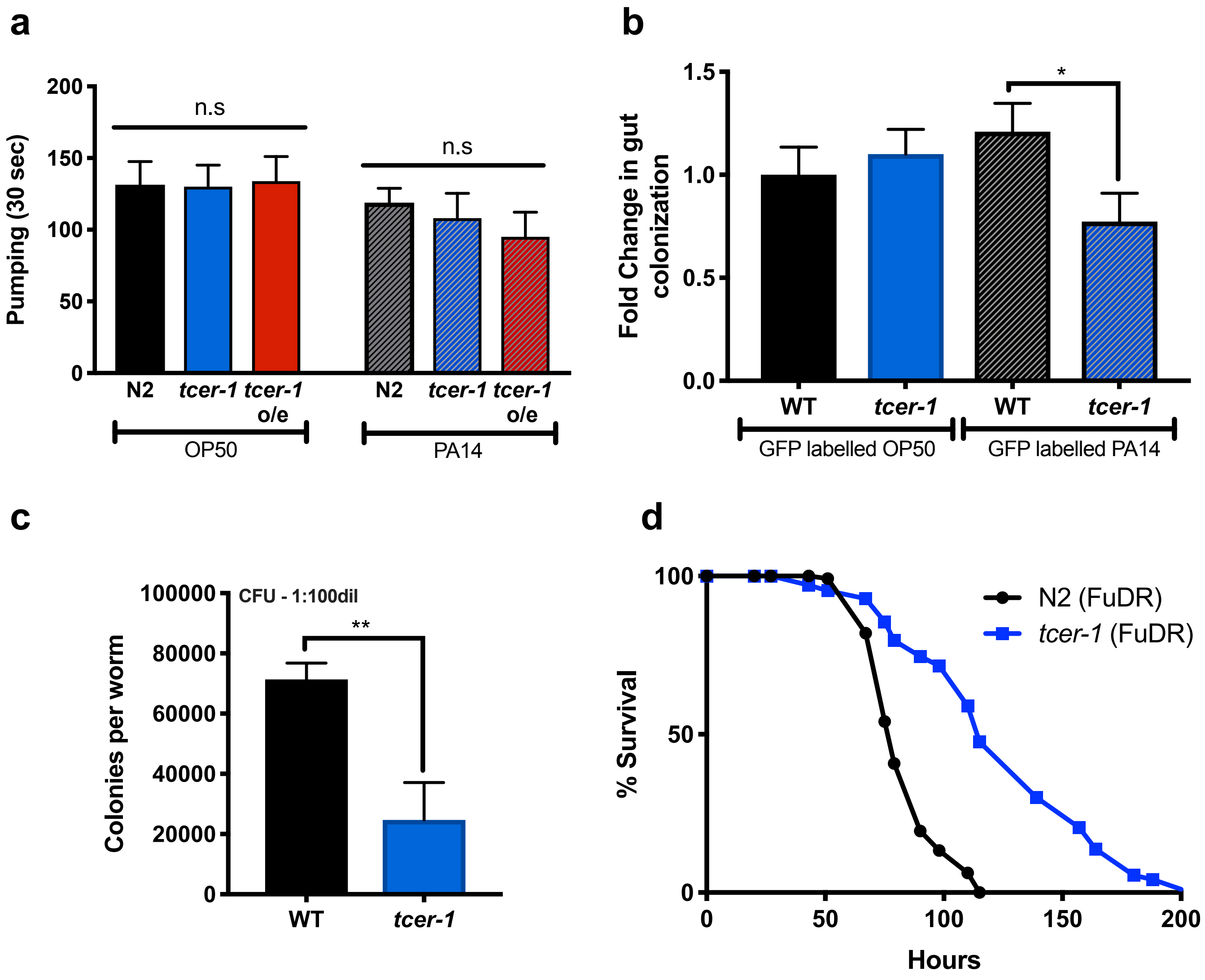

(a) Pharyngeal pumping. The pumping rate between wild type (WT, black), *tcer-1* mutants (blue) and a TCER-1-overexpressing strain (red) showed no significant difference using the ordinary one-way anova statistical test on normal OP50 and pathogenic PA14 food source. Data shown is combined from three independent biological replicates with 15-20 animals tested per strain, per replicate. **(b)** Measurement of bacterial ingestion. GFP in intestinal lumens of wild-type (WT, black) and *tcer-1* mutants (blue) was measured 24h after transfers of age-matched L4 larvae to GFP labelled OP50 or PA14. Significance was calculated using the unpaired one-tailed t test * P<0.05 **(c)** CFU Analysis. Number of colonies (CFU) generated by bacteria isolated from wild-type (black) or *tcer-1* mutants (blue) exposed to PA14. Significance was calculated using the unpaired two-tailed t test ** P<0.01. **(d)** Effect of bagging/matricide on PA14 resistance. L4-stage animals exposed to 100 µg/ml FuDR-containing plates for 24h followed by transfer to PA14 plates**.** WT (m = 59.35 ± 1.3, n = 89/106) and *tcer-1* (m = 98.6 ± 3.5, n = 83/109, P vs WT <0.0001) (m = 114.18 ± 3.9, n = 81/106, P vs *glp-1* <0.001). Surival was calculated using the Kaplan Meier statistical analysis and data shown as mean (m) ± standard error of the mean (SEM).

Supplementary Figure 3: TCER-1-repressed genes are enriched for roles involved in immunity and stress resistance.

a. Subset of TCER-1 DOWN genes predicted to be invovled in immunity and stress related functiions as per the associated GO terms

WB Gene ID	Gene Description	GO Term	PMK-1 dependent (Troemel et al. 2006)	Identified as Induced by PA14
WBGene00003099	LYSozyme (*lys-10*)	Increased pathogene susceptability	Troemel et al	X
WBGene00016669	Invertebrate LYSozyme (*ilys-2*)	lysozyme activity, Defense to Gram-ve Bacterium		X
WBGene00005003	SaPosin-like Protein family (*spp-18*)	Innate Immune Response		X X
WBGene00016670	Invertebrate LYSozyme (*ilys-3*)	lysozyme activity		X
WBGene00015759	hypothetical protein (*C14C6.5*)	Innate Immune Response	Troemel et al	X
WBGene00003093	LYSozyme (*lys-4*)	lysozyme activity		
WBGene00007875	Downstream Of DAF-16 (regulated by DAF-16) (*dod-24*)	Innate Immune Response, Defense to Gram-ve Bacterium	Troemel et al	X X X
WBGene00009813	Histidine ammonia-lyase (*haly-1*)	Stress Response to Zinc Ion		x
WBGene00019779	hypothetical protein (*M60.2*)	Innate Immune Response, Defense to Gram-ve Bacterium		X
WBGene00003096	LYSozyme (*lys-7*)	Innate Immune Response, Defense to Gram-ve Bacterium		
WBGene00000556	CaeNaCin (Caenorhabditis bacteriocin) (*cnc-2*)	Innate Immune Response, Defense to Gram-ve Bacterium		
WBGene00001772	Glutathione S-Transferase (*gst-24*)	Innate Immune Response		x
WBGene00010124	hypothetical protein (*F55G11.4*)	Defense to Gram-ve Bacterium	Troemel et al	X
WBGene00020455	F-box A protein (*fbxa-59*)	Innate Immune Response		X X
WBGene00000599	Cuticle collagen 10 (*col-10*)	Response to Oxidative stress		
WBGene00020658	hypothetical protein (*T21F4.1*)	Innate Immune Response		x
WBGene00016542	hypothetical protein (*C39H7.4*)	Innate Immune Response		X X
WBGene00003877	Peptide transporter family 1 (*pept-1*)	Response to oxidative stress		
WBGene00002024	Heat Shock Protein (*hsp-43*)	Stress Response		
WBGene00015052	C-type LECtin (*clec-52*)	Defense to Gram-ve Bacterium		X
WBGene00009433	hypothetical protein (*F35E12.9*)	Innate Immune Response, Defense to Gram-ve Bacterium		X X
WBGene00009429	hypothetical protein (*F35E12.5*)	Innate Immune Response, Defense to Gram-ve Bacterium	Troemel et al	X
WBGene00018910	hypothetical protein (*F56A4.2*)	Innate Immune Response		X
WBGene00021224	C-type LECtin (*clec-209*)	Innate Immune Response		X
WBGene00000717	COLlagen (*col-144*)	Response to Oxidative stress		
WBGene00009213	THaumatiN family (*thn-1*)	Innate Immune Response		
WBGene00018384	hypothetical protein (*F43C11.7*)	Innate Immune Response, Defense to Gram-ve Bacterium		
WBGene00016769	hypothetical protein (*C49C8.5*)	Innate Immune Response		X X X
WBGene00022645	hypothetical protein (*ZK6.11*)	Innate Immune Response	Troemel et al	X
WBGene00011647	Non-centrosomal microtubule array protein 1 (*noca-1*)	Apoptotic Process		
WBGene00022644	Downstream Of DAF-16 (regulated by DAF-16) (*dod-19*)	Innate Immune Response	Troemel et al	X X
WBGene00008199	hypothetical protein (*C49C3.9*)	Innate Immune Response, Defense to Gram-ve Bacterium		X X X
WBGene00004055	Mitogen-activated protein kinase pmk-1 (*pmk-1*)	Innate Immune Response, Defense to Gram-ve Bacterium		
WBGene00019427	Autophagic-related protein 16.2 (*atg-16.2*)	Defense to Gram-ve Bacterium		
WBGene00000900	Cell surface receptor *daf-4* (*daf-4*)	Innate Immune Response		
WBGene00008634	hypothetical protein (*F10A3.4*)	Innate Immune Response		
WBGene00001333	Ezrin/Radixin/Moesin (*erm-1*)	Apoptotic Process		
WBGene00002008	Heat Shock Protein (*hsp-4*)	Apoptotic Process, Stress Response		
WBGene00018760	Infection Response Gene (*irg-3*)	Innate Immune Response		X X X
WBGene00017262	hypothetical protein (*F08F3.4*)	Innate Immune Response		X
WBGene00001029	DNaJ domain (prokaryotic heat shock protein) (*dnj-11*)	Stress Response		
WBGene00020549	Glycylpeptide N-tetradecanoyltransferase (*nmt-1*)	Apoptotic Process		

X = Induced by PA14 (Curated from Wormbase); X = Troemel et al 2006; X = Shapira et al 2006; X = Twumasi-Boeteng and Shapira 2012

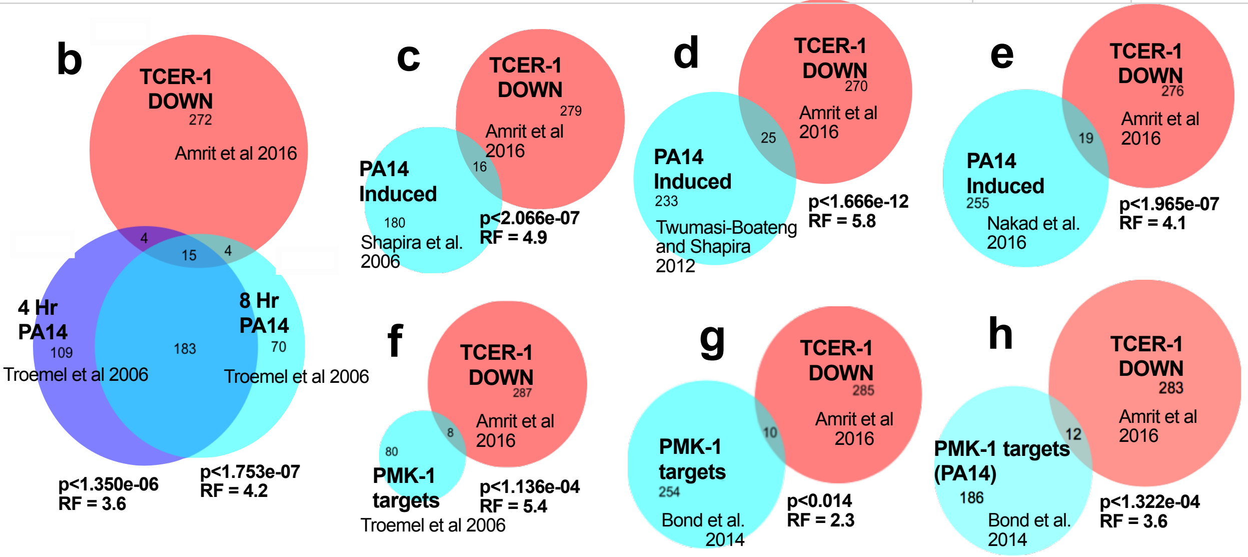

(a) Of the 295 genes predicted to be down regulated >1.5 fold by TCER-1 (TCER-1 'DOWN' class), 42 listed here have reported immunity and/or stress-related functions based on the associated GO terms. Of these, many have been shown to be upregulated upon infection by PA14 and/or under PMK-1 control (shown in column three, key under table). **(b-e)** Graphical representation of the comparison of overlap between TCER-1 'DOWN' genes with lists from previous studies that identified genes induced upon PA14 infection. **(f-h)** Graphical representation of the comparison of overlap between TCER-1 'DOWN' genes with studies that identified PMK-1 targets. The statistical significance of the overlap observed between the DOWN group and each gene lists are shown under the respective panels as is with the degree of over-enrichment, calculated as representation factor (RF) on nemates.org (http://nemates.org/MA/progs/overlap_stats.html).

Supplementary Figure 4: Expression of PMK-1 target genes in *tcer-1* mutants on OP50 and PA14 exposure.

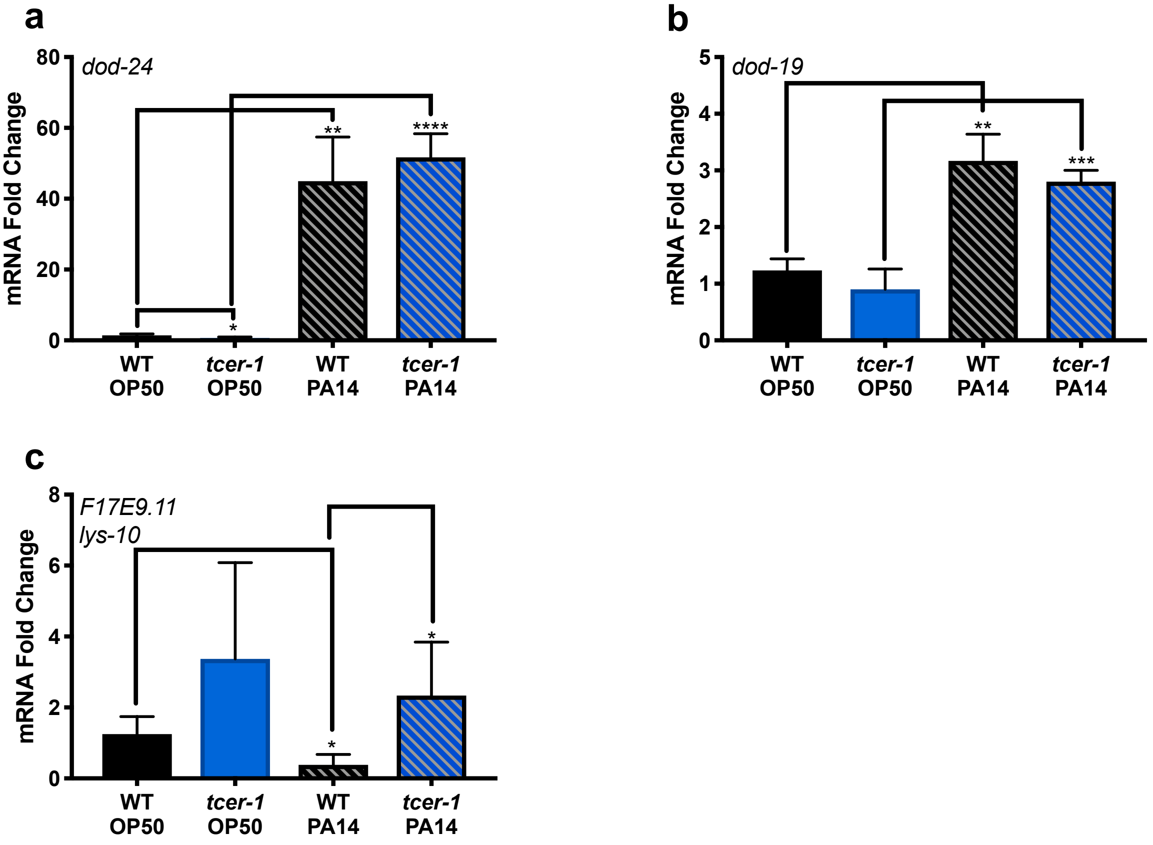

(a-c) mRNA levels of *dod-24, dod-19* and *lys-10* compared between day 1 wild-type animals (WT, black) and *tcer-1* mutants (blue) maintained on OP50 (solid bars) or exposed as L4s to PA14 for 8 hours (hashed bars). Data combined from 2-5 independent biological replicates, each including three technical replicates. Error bars denote the standard error of the mean. Asterisks represent the statistical significance of differences observed in an unpaired, one-tailed t-test with P values 0.05 (*), 0.01 (**), 0.01(***) or < 0.00001 (****).

Supplementary Figure 5: Expression of genes PMK-1-independent genes upregulated by PA14 exposure in *tcer-1* mutants on OP50 and PA14 exposure.

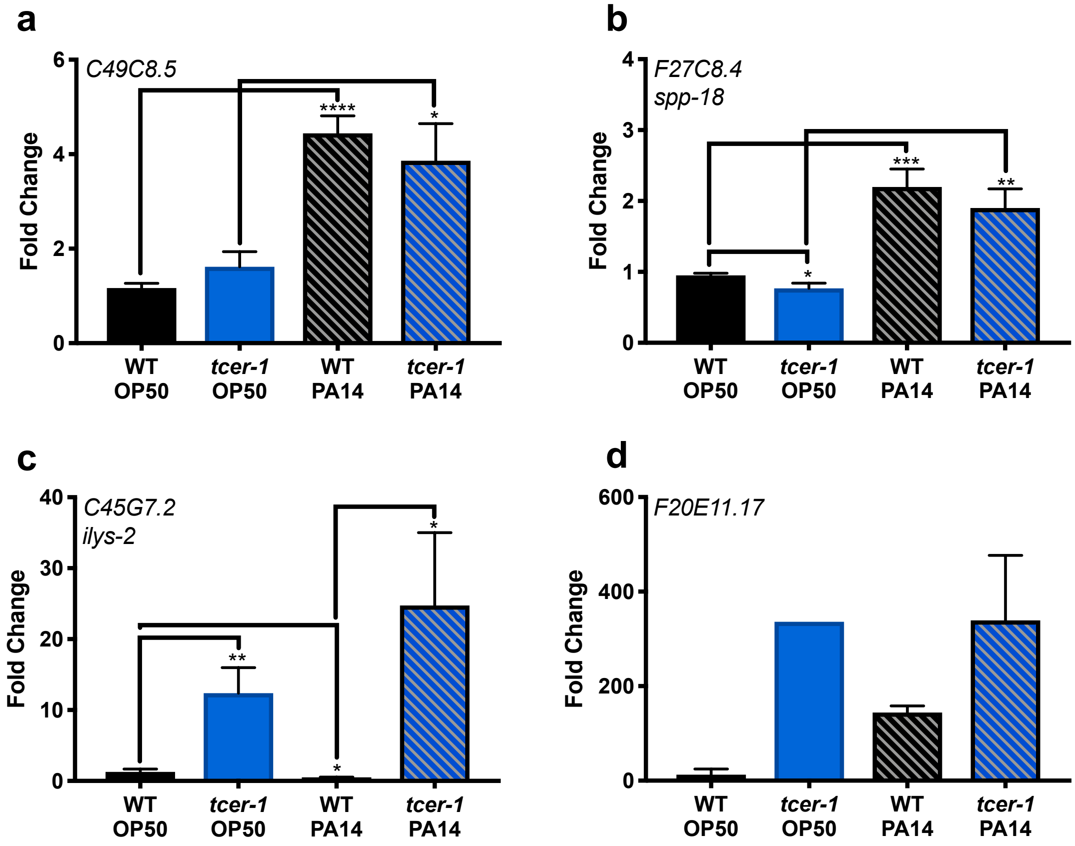

(a-d) mRNA levels of *C49C8.5, spp-18, ilys-2* and *F20E11.17* compared between Day 1 wild-type *C. elegans* (WT, black), *tcer-1* mutants (blue) maintained on OP50 (solid bars) or exposed as L4s to PA14 for 8 hours (hashed bars). Data combined from 2-5 independent biological replicates, each including three technical replicates. Error bars denote the standard error of the mean. Asterisks represent the statistical significance of differences observed in an unpaired, one-tailed t-test with P values 0.05 (*), 0.01 (**), 0.01(***) or < 0.00001 (****).

Supplementary Figure 6: TCER-1 represses expression of key transcription factors mediating UPR^{mt} and hypoxia response.

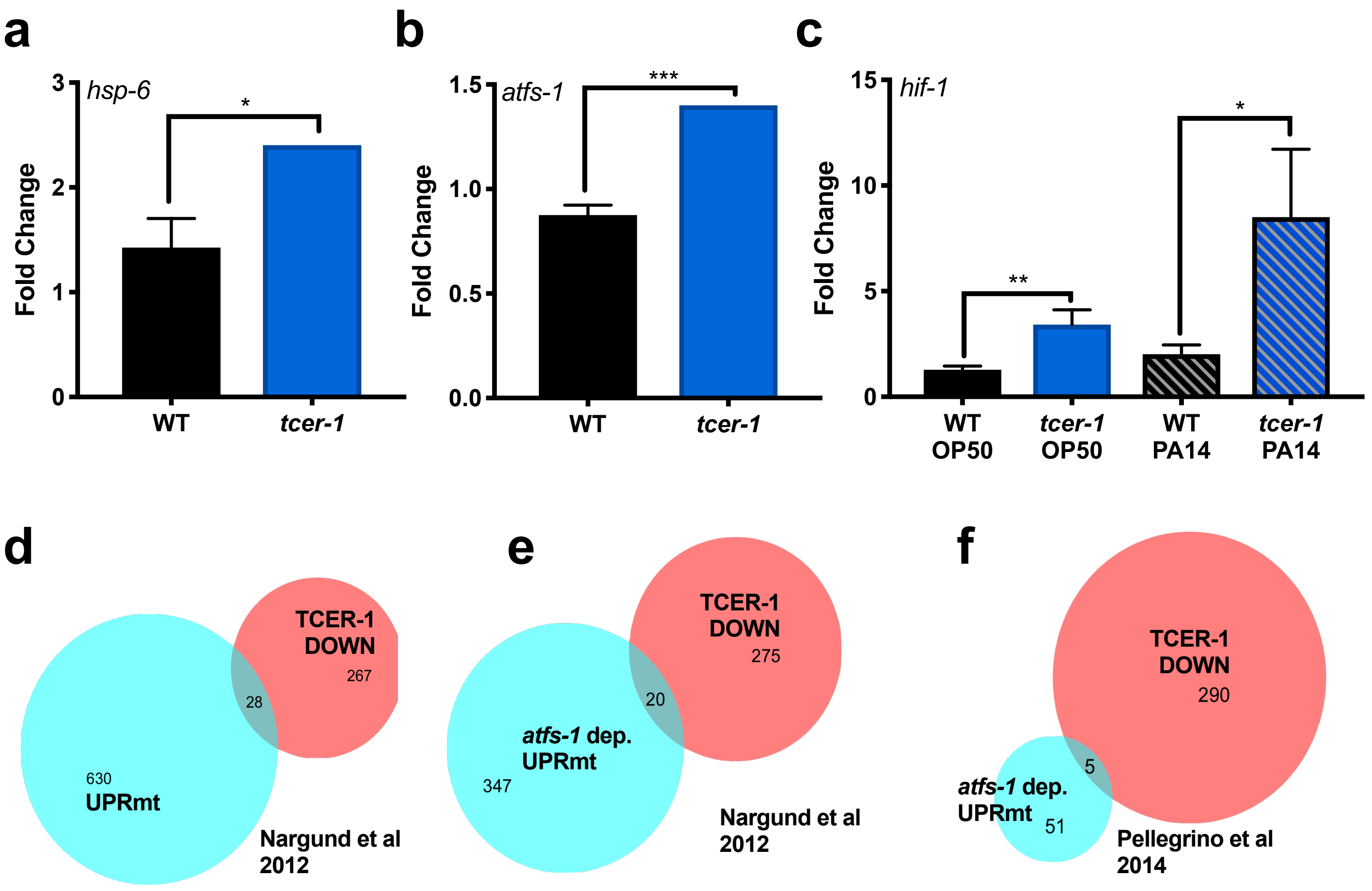

Fig	RNA-Seq Gene Class	Comparison Study	Details	Statistical Significance	Representation Factor
d	TCER-1 Downregulated	Nargund et al.	Mito UPR Targets	p < 6.282e-06	2.5
e	TCER-1 Downregulated	Nargund et al.	atfs dependent Mito UPR Targets	p < 4.091e-06	3.3
f	TCER-1 Downregulated	Pellegrino et al.	Mito UPR Targets and PA14 shared	p < 0.002	5.3

(a-c) mRNA levels of *hsp-6, atfs-1* and *hif-1* compared between day 1 wild-type (WT, black), *tcer-1* (blue) maintained on OP50 (solid bars) or exposed as L4s to PA14 for 8 hours (hashed bars in c). Data combined from 2-5 independent biological replicates, each including three technical replicates. Error bars denote standard error of the mean. Asterisks represent the statistical significance of differences observed in an unpaired, one-tailed t-test with P values 0.05 (*) or 0.01(***). **(d-f)** Graphical representation of the comparison of overlap between TCER-1 'DOWN' class with lists from previous genomic studies identifying UPR^{mt} genes. P values for associated probability/overlap and representation factor (RF) were calculated on nemates.org (http://nemates.org/MA/progs/overlap_stats.html).

Supplementary Figure 7: *tcer-1* mRNA expression in animals exposed to PA14.

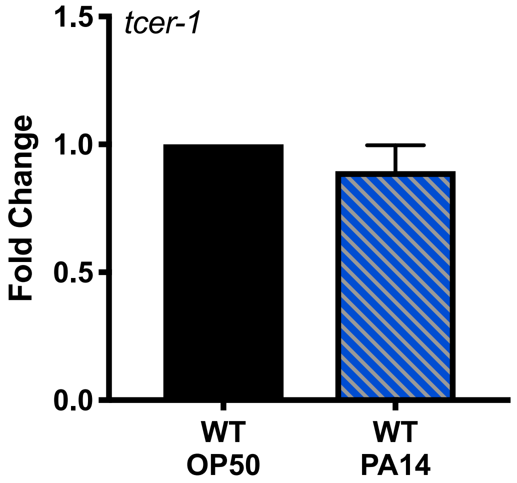

mRNA levels of *tcer-1* compared between Day 1 wild-type adults grown on OP50 (black) vs. those exposed to PA14 for 12h (blue). Data from 10 independent replicates showed highly variable effects with decreased levels in 6 cases, no change in one and elevation in three. The combined data from all these replicates is shown here and indicates no statistically significant difference in expression by a unpaired, 2-tailed t-test.

Supplementary Figure 8: TCER-1 expression in intestinal- and germline-nuclei is reduced with age.

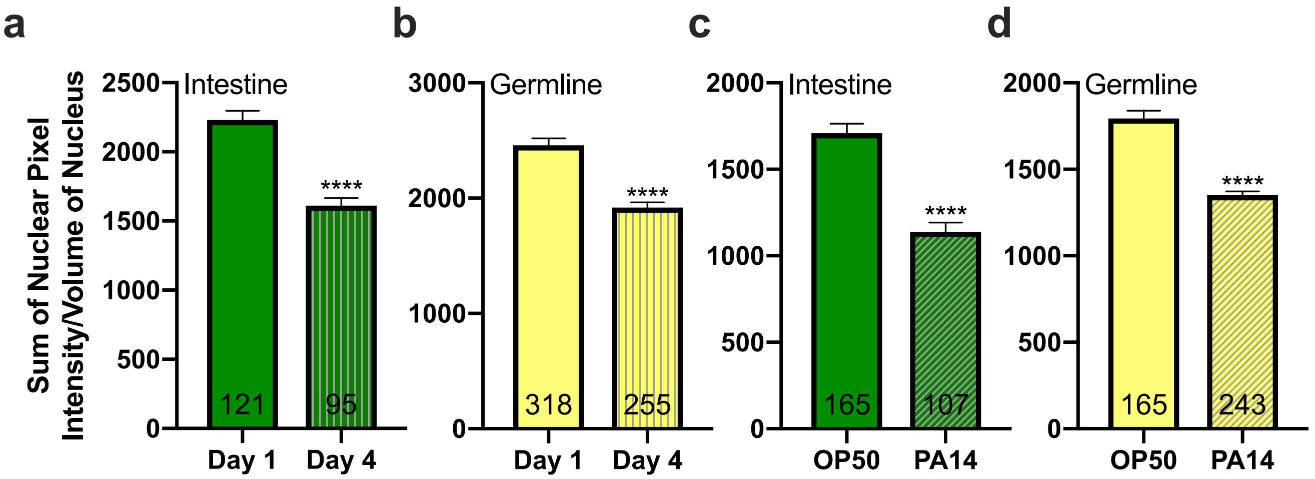

(a, b) and upon pathogen exposure **(c, d).** TCER-1::GFP levels compared between nuclei of intestinal cells (a, c, green bars) and germ cells (b, d, yellow bars) in **(a,b)** Day 1 (solid bars) vs. and Day 4 (vertical hashed bars) adults, and **(c, d)** Day 1 adults exposed to PA14 for 12h (angular hashed bars) vs. their age-matched counterparts maintained on OP50 (solid bars). Shown here is data obtained by measuring the ratio of sum of pixel intensities of each nucleus to its total volume, and comparing these values between the different ages/conditions. This obviated potential skewing of data due to different nuclear sizes. The differences observed by comparing average pixel intensity per nucleus (Fig. 4g-j) are still maintained. 'n' signifies the total number of nuclei in which GFP intensity was measured from intestines (9-19 adults per strain/condition) and germlines (7-13 adults per strain/condition) Asterisks represent the statistical significance of the differences in expression in an unpaired, two-tailed t-test with P values <0.0001 (****). Error bars represent standard error of the mean.

Supplementary Figure 9: Loss of *tcer-1* delays paralysis in amyloid β proteotoxicity model.

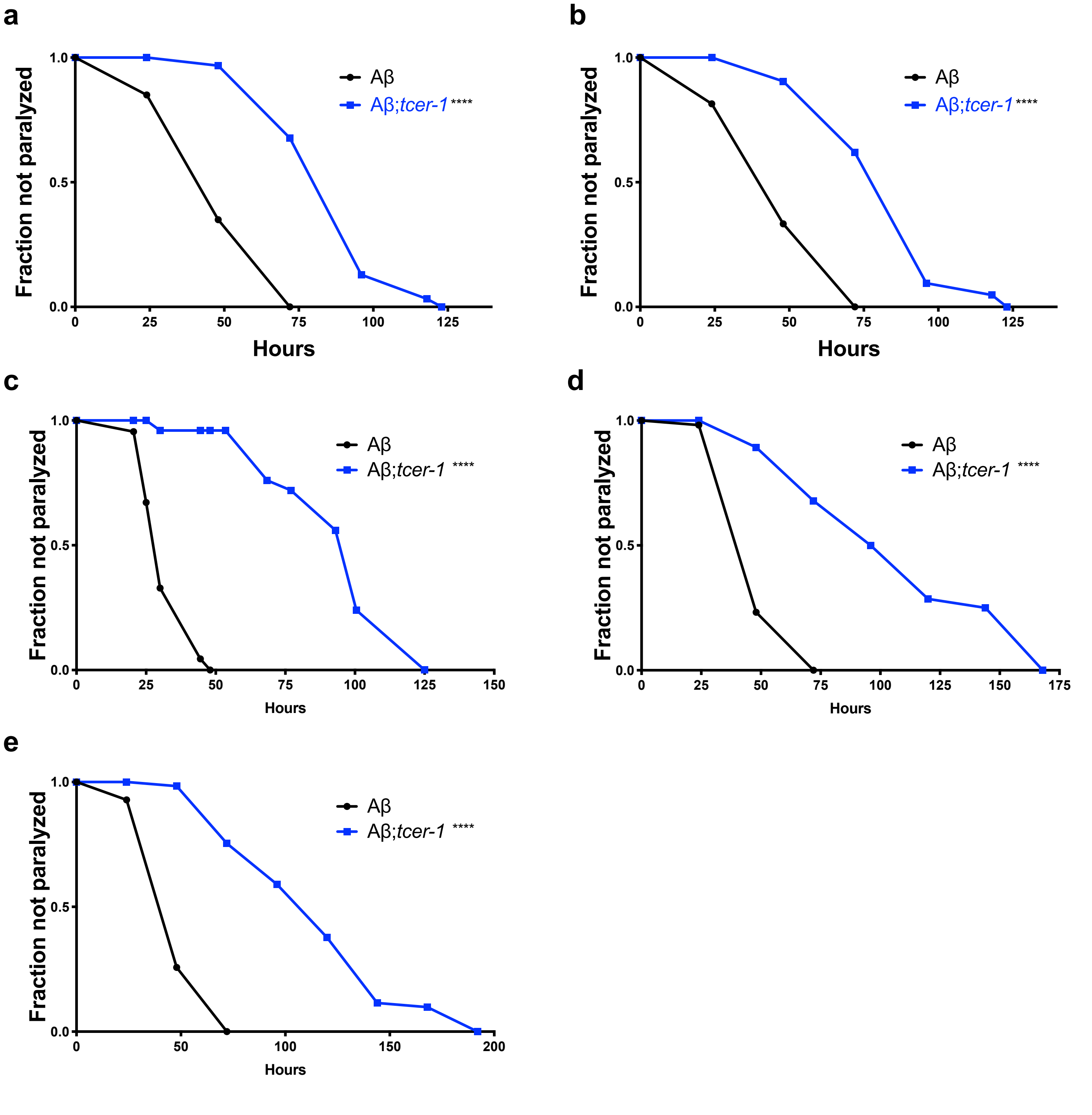

Onset of paralysis measured in the amyloid β proteotoxicity model strain GMC101 expressing the human $A\beta_{1\text{-}42}$ peptide in *C. elegans* body wall muscles (Aβ, black) and Aβ strain carrying a *tcer-1* mutation (Aβ;*tcer-1*, blue). Data from five independent replicates, shown in a to e, show significant delay in onset of paralysis in the Aβ; *tcer-1* strain compared to control. Kaplan Meier analysis of mean paralysis **(a)** Aβ: m = 52.8h, n = 20; Aβ;*tcer-1:* m = 90.48h, n = 31, P <0.0001. **(b)** Aβ: m = 51.56h, n = 27; Aβ;*tcer-1:* m = 86.9h, n = 21, P <0.0001. **(c)** Aβ: m = 33.07h, n = 67; Aβ;*tcer-1:* m = 95.02h, n = 25, P <0.0001. **(d)** Aβ: m = 53.14h, n = 56; Aβ;*tcer-1:* m = 110.6h, n = 28, P <0.0001. **(e)** Aβ: m = 52.46h, n = 70; Aβ;*tcer-1:* m = 118h, n = 61, P <0.0001.

Amrit et al., Supplementary Table 1. Survival of wild type and mutant strains upon exposure to *P. aeruginosa* PA14

1A: Effect of *tcer-1, glp-1* and *pmk-1* mutations on survival upon PA14 exposure

Strain	Genotype	Trial 1[#]			Bonferroni P values			
		n = obs/ total	Mean	SE ^	P (*vs* N2)	P (*vs glp-1*)	P (*vs tcer-1;glp-1*)	P (*vs tcer-1*)
N2	Wildtype	86/106	59.35	+/- 1.29				
CF1903	*glp-1*	102/109	72.86	+/- 2.06	0.0001			
CF2154	*tcer-1;glp-1*	81/106	114.18	+/- 3.86	0.001	0.001		
CF2166	*tcer-1*	83/109	98.6	+/- 3.49	0.001	0.001	0.007	
AGP97	*pmk-1*	88/101	27.81	+/- 0.89	0.001	0.001	0.001	0.001
AGP202	*glp-1;pmk-1*	92/100	68.75	+/- 3.34	0.7878	1	0.001	0.0001
AGP203	*tcer-1;glp-1;pmk-1*	103/103	42.65	+/- 2.21	0.0001	0.001	0.001	0.001
AGP213	*tcer-1;pmk-1*	59/101	52.35	+/- 2.61	0.2608	0.0001	0.001	0.001
Trial 2[#]					Bonferroni P values			
N2	Wildtype	75/100	63.49	+/- 1.34				
CF1903	*glp-1*	98/101	72.71	+/- 2.31	0.0167			
CF2154	*tcer-1;glp-1*	57/75	102.1	+/- 5.16	0.001	0.0001		
CF2166	*tcer-1*	84/100	93.82	+/- 4.35	0.0001	0.0001	1	
AGP97	*pmk-1*	92/100	23.77	+/- 0.87	0.001	0.001	0.001	0.001
AGP202	*glp-1;pmk-1*	94/102	54.72	+/- 2.11	0.0771	0.0001	0.001	0.001
AGP203	*tcer-1;glp-1;pmk-1*	91/105	43.78	+/- 2.34	0.001	0.001	0.001	0.001
AGP213	*tcer-1;pmk-1*	97/102	45.95	+/- 1.82	0.0001	0.001	0.001	0.001
Trial 3					Bonferroni P values			
N2	Wildtype	84/100	57.71	+/- 1.46				
CF1903	*glp-1*	96/102	64.22	+/- 2.25	0.4715			
CF2154	*tcer-1;glp-1*	56/100	101.4	+/- 5.14	0.001	0.001		
CF2166	*tcer-1*	54/100	76.99	+/- 3.32	0.001	0.0005	0.0103	
AGP97	*pmk-1*	94/100	16.95	+/- 0.69	0.001	0.001	0.001	0.001
AGP202	*glp-1;pmk-1*	94/100	40.09	+/- 1.96	0.0001	0.001	0.001	0.001
AGP203	*tcer-1;glp-1;pmk-1*	83/100	37.26	+/- 1.73	0.001	0.001	0.001	0.001
AGP213	*tcer-1;pmk-1*	86/100	33.76	+/- 1.41	0.001	0.001	0.001	0.001

1B: Effect of *daf-16* mutation on PA14 resistance of different strains

Strain	Genotype	Trial 1			Bonferroni P values			
		n = obs/ total	Mean	SE ^	P (*vs* N2)	P (*vs glp-1*)	P (*vs tcer-1*)	P (*vs tcer-1;glp-1*)
N2	Wildtype	45/100	78.67	+/-4.22				
CF1038	*daf-16*	58/100	63.37	+/-1.63	0.001			
CF2166	*tcer-1*	45/100	91.77	+/-4.26	0.0803			
AGP214	*daf-16;tcer-1*	45/100	67.59	+/-3.11	0.0184		0.0001	
Trial 2					Bonferroni P values			
N2	Wildtype	61/80	46.86	+/-1.2				
CF1903	*glp-1*	75/81	80.15	+/-3.08	0.001			
CF2154	*tcer-1;glp-1*	50/81	85.49	+/-4.62	0.001	0.6996		
CF1880	*daf-16;glp-1*	66/80	58.1	+/-2.81	0.0013	0.0001		0.0001
Trial 3					Bonferroni P values			
N2	Wildtype	59/75	50.84	+/-1.35		0.0001		0.001
CF1903	*glp-1*	56/76	62.9	+/-2.55	0.0001			0.0765
CF2154	*tcer-1;glp-1*	38/76	72.08	+/-3.10	0.001	0.0765		
CF1880	*daf-16;glp-1*	32/75	50.73	+/-2.22	1	0.006		0.0001
Trial 4					Bonferroni P values			
N2	Wildtype	45/57	30.83	+/-1.87				
CF1903	*glp-1*	79/99	45.67	+/-2.3	0.0001			
CF2154	*tcer-1;glp-1*	62/100	54.54	+/-3.93	0.0001	0.3899		
CF1880	*daf-16;glp-1*	85/104	40.39	+/-2.36	0.005	0.2676		0.0045
AGP215	*daf-16;tcer-1;glp-1*	56/77	33.98	+/-1.97	1	0.0003		0.0001

[#] Data from Table 1A (Trial 1) and (Trial2) represented in Fig. 1a and Fig. 5e respectively.

Amrit et al., Supplementary Table 2. Survival of wildtype and *tcer-1* mutants exposed to *P. aeruginosa* PA01 and *S. aureus* NCTC8325

Plate Type	Pathogen	Strain	Genotype	n = obs/ total	Mean	SE ^	p (*vs* N2)
**2A. Survival of wildtype and *tcer-1* mutants exposed to PA01 on SK plates and NGM plates**							
				**Trial 1#**			
SK	PA01	N2	Wildtype	118 / 142	126.43	+/- 2.38	
SK	PA01	CF2166	*tcer-1*	143 / 173	139.98	+/- 2.65	**0.001**
**Trial 2**							
SK	PA01	N2	Wildtype	105 / 127	110.80	+/- 3.1	
SK	PA01	CF2166	*tcer-1*	111 / 133	110.10	+/- 3.14	**0.931**
**Trial 3**							
SK	PA01	N2	Wildtype	112 / 124	121.27	+/- 2.89	
SK	PA01	CF2166	*tcer-1*	83 / 129	131.64	+/- 3.75	**0.03**
**Trial 4**							
NGM	PA01	N2	Wildtype	83 / 99	155.56	+/- 4.43	
NGM	PA01	CF2166	*tcer-1*	50 / 111	151.76	+/- 6.12	**0.8**
**Trial 5**							
NGM	PA01	N2	Wildtype	124 / 147	136.19	+/- 2.33	
NGM	PA01	CF2166	*tcer-1*	110 / 149	143.96	+/- 3.92	**0.013**
**Trial 6**							
NGM	PA01	N2	Wildtype	118 / 138	122.73	+/- 2.66	
NGM	PA01	CF2166	*tcer-1*	116 / 154	125.94	+/- 2.92	**0.4**
**2B. Survival of wildtype and tcer-1 mutants exposed to *S. aureus* (NCTC8325) on BHI plates**							
**Trial 1#**							
BHI	*S. aureus* (NCTC8325)	N2	Wildtype	97 / 111	94.67	+/- 1.44	
BHI	*S. aureus* (NCTC8325)	CF2166	*tcer-1*	145 / 169	109.27	+/- 1.5	**0.001**
**Trial 2**							
BHI	*S. aureus* (NCTC8325)	N2	Wildtype	110 / 120	105.14	+/- 2.27	
BHI	*S. aureus* (NCTC8325)	CF2166	*tcer-1*	98 / 116	115.05	+/- 2.69	**0.001**

Data from Tables 2A (Trial 1) and 2B (Trial 1) represented in Figs. 1b and 1c respectively.

**Amrit et al., Supplementary Table 3.** Effect of *tcer-1* mutation on resistance against oxidative and ER stress						
**3A. Survival of wild type and mutant strains upon exposure to tBOOH**						
**Strain**	**Genotype**	**Trial 1#**			**Bonferroni P values**	
		**n = obs/ total**	**Mean**	**SE ^**	**p (*vs* N2)**	**p (*vs glp-1*)**
N2	Wildtype	57/98	17.32	+/- 1.05		
CF2166	*tcer-1*	33/100	25.65	+/- 1.65	0.0001	
CF1903	*glp-1*	79/100	27.88	+/- 1.46	0.0001	
CF2154	*tcer-1;glp-1*	29/100	56.31	+/- 5.06	0.0001	0.0001
		**Trial 2**			**Bonferroni P values**	
N2	Wildtype	28/90	15.19	+/- 1.3		
CF2166	*tcer-1*	33/100	25.78	+/- 3.32	0.0248	
CF1903	*glp-1*	51/100	50.59	+/- 3.28	0.001	
CF2154	*tcer-1;glp-1*	38/100	30.84	+/- 3.59	0.0009	0.0001
		**Trial 3**			**Bonferroni P values**	
N2	Wildtype	44/80	17.81	+/- 1.37		
CF2166	*tcer-1*	38/80	23.37	+/- 1.3	0.1637	
CF1903	*glp-1*	69/80	33.51	+/- 2.24	0.0008	
CF2154	*tcer-1;glp-1*	56/80	49.83	+/- 4.81	0.0097	0.0092
**3B. Survival of wild type and mutant strains upon exposure to Tunicamycin**						
		**Trial 1**			**Bonferroni P values**	
N2	Wildtype	89/100	16.58	+/- 0.65		
CF2166	*tcer-1*	91/101	16.01	+/- 0.56	1	
CF1903	*glp-1*	107/107	18.41	+/- 1.27	1	
CF2154	*tcer-1;glp-1*	79/100	21.73	+/- 1.25	0.0012	0.3749
		**Trial 2**			**Bonferroni P values**	
N2	Wildtype	91/104	18.13	+/- 0.64		
CF2166	*tcer-1*	87/104	17.27	+/- 0.67	1	
CF1903	*glp-1*	96/108	18.81	+/- 1.03	1	
CF2154	*tcer-1;glp-1*	100/101	24.91	+/- 2.96	0.0021	0.1118
		**Trial 3**			**Bonferroni P values**	
N2	Wildtype	57/79	17.66	+/- 0.73		
CF2166	*tcer-1*	100/102	16.7	+/- 0.7	1	
CF1903	*glp-1*	99/111	21.91	+/- 1.16	0.0018	
CF2154	*tcer-1;glp-1*	87/106	20.83	+/- 1.3	0.401	1

Data from Table 3A (Trial 1) represented in Fig. 1e.

Amrit et al., Supplementary Table 4. Effect of TCER-1 expression in different tissues on resistance against *P. aeruginosa* PA14

4A. Survival of strains expressing TCER-1 in different tissues of *tcer-1* mutants upon exposure to *P. aeruginosa* PA14

Strain	Genotype	Tissue of TCER-1 expression (promoter)	Trial 1[#]			Bonferroni P-value		
			n = obs/ total	Mean	SE ^	P (*vs* N2)	P (*vs glp-1*)	P (*vs tcer-1*)
N2	Wildtype	Control	87/111	60.68	+/- 0.95			
CF1903	*glp-1*	Control	98/104	79.22	+/- 2.11	0.001		
CF2154	*tcer-1;glp-1*	Control	55/78	80.3	+/- 3.3	0.001	1	
CF2166	*tcer-1*	Control	53/102	96.31	+/- 2.74	0.001	0.0001	
AGP173	*tcer-1*	*tcer-1 (Endogenous promoter)*	48/63	70.91	+/- 3.4	0.002	0.7498	0.0001
AGP194	*tcer-1*	*Muscle (myo-3)*	88/100	64	+/- 2.7	0.144	0.003	0.001
AGP120	*tcer-1*	*Hypodermis (col-12)*	72/102	71.67	+/- 3.11	0.0001	1	0.0001
AGP92	*tcer-1*	*Intestine (gly-19)*	77/99	55.33	+/- 3.08	1	0.0001	0.001
AGP170	*tcer-1*	*Neurons (rgef-1)*	85/107	62.32	+/- 1.9	1	0.0001	0.001
			Trial 2			Bonferroni P-value		
N2	Wildtype	Control	75/102	63.75	+/- 1.03			
CF1903	*glp-1*	Control	96/118	84.79	+/- 2.72	0.001		
CF2154	*tcer-1;glp-1*	Control	49/75	86.07	+/- 4.44	0.0005	1	
CF2166	*tcer-1*	Control	33/100	102.04	+/- 3.14	0.001	0.0001	
AGP194	*tcer-1*	*Muscle (myo-3)*	70/100	73.95	+/- 3.9	0.1159	0.3085	0.0001
AGP120	*tcer-1*	*Hypodermis (col-12)*	55/100	87.29	+/- 4.64	0.0001	1	0.0087
AGP92	*tcer-1*	*Intestine (gly-19)*	50/100	71.83	+/- 4.41	1	0.1522	0.0001
AGP170	*tcer-1*	*Neurons (rgef-1)*	87/100	59.16	+/- 1.71	0.3631	0.001	0.001
			Trial 3			Bonferroni P-value		
N2	Wildtype	Control	54/65	85.93	+/- 2.07			
CF2166	*tcer-1*	Control	86/106	113.03	+/- 3.28	0.001		
CF2858	*tcer-1*	*tcer-1 (Endogenous promoter)*	88/101	100.88	+/- 2.6	0.0001		0.0098
			Trial 4			Bonferroni P-value		
N2	Wildtype	Control	74/86	84.68	+/- 1.88			
CF2166	*tcer-1*	Control	64/91	115.73	+/- 3.98	0.001		
CF2858	*tcer-1*	*tcer-1 (Endogenous promoter)*	78/93	101.12	+/- 4.08	0.0047		0.0394
			Trial 5			Bonferroni P-value		
N2	Wildtype	Control	66/81	79.56	+/- 1.52			0.001
CF2166	*tcer-1*	Control	46/75	113.11	+/- 2.96	0.001		
AGP170	*tcer-1*	*Neurons (rgef-1)*	100/110	78.65	+/- 1.14	1		0.001
			Trial 6			Bonferroni P-value		
N2	Wildtype	Control	59/73	61.92	+/- 2.4			0.001
CF2166	*tcer-1*	Control	65/77	85.37	+/- 2.7	0.001		
AGP170	*tcer-1*	*Neurons (rgef-1)*	101/112	53.68	+/- 1.14	0.0012		0.001

4B. Survival of strains expressing TCER-1 in different tissues of *tcer-1;glp-1* mutants upon exposure to *P. aeruginosa* PA14

Strain	Genotype	Expression	Trial 1			Bonferroni P-value		
			n = obs/ total	Mean	SE ^	P (*vs* N2)	P (*vs glp-1*)	P (*vs tcer-1;glp-1*)
N2	Wildtype	Control	52/60	46.6	+/- 1.23			
CF1903	*glp-1*	Control	47/60	68.48	+/- 2.39	0.001		
CF2154	*tcer-1;glp-1*	Control	43/60	63.84	+/- 4.86	0.0001	0.6025	
AGP172	*tcer-1;glp-1*	*tcer-1 (Endogenous promoter)*	36/60	82.26	+/- 6.29	0.0001	0.4028	0.3869
AGP79	*tcer-1;glp-1*	*Muscle (myo-3)*	40/50	33.23	+/- 2.26	0.0001	0.001	0.001
AGP122	*tcer-1;glp-1*	*Hypodermis (col-12)*	17/29	63.02	+/- 9.87	1	1	1
AGP91	*tcer-1;glp-1*	*Intestine (gly-19)*	47/75	36.27	+/- 4.7	0.0001	0.001	0.0001
AGP171	*tcer-1;glp-1*	*Neurons (rgef-1)*	39/61	56.5	+/- 5.28	1	0.2669	0.6632
			Trial 2[#]			Bonferroni P-value		
N2	Wildtype	Control	63/82	47.61	+/- 1.29			
CF1903	*glp-1*	Control	73/82	59.52	+/- 1.91	0.0001		
CF2154	*tcer-1;glp-1*	Control	50/80	67.98	+/- 4.46	0.0003	0.3984	
AGP172	*tcer-1;glp-1*	*tcer-1 (Endogenous promoter)*	09/`10	26.3	+/- 6.79	0.0004	0.0001	0.0001
AGP79	*tcer-1;glp-1*	*Muscle (myo-3)*	66/80	25.03	+/- 1.64	0.001	0.001	0.001
AGP122	*tcer-1;glp-1*	*Hypodermis (col-12)*	39/60	47.15	+/- 3.29	1	0.0594	0.0068
AGP91	*tcer-1;glp-1*	*Intestine (gly-19)*	64/80	22.45	+/- 1.23	0.001	0.001	0.001
AGP171	*tcer-1;glp-1*	*Neurons (rgef-1)*	53/80	54.08	+/- 3.61	0.7272	1	0.0992
			Trial 3[#]			Bonferroni P-value		
N2	Wildtype	Control	87/111	60.68	+/- 0.95			
CF1903	*glp-1*	Control	98/104	79.22	+/- 2.11	0.001		
CF2154	*tcer-1;glp-1*	Control	55/78	80.3	+/- 3.3	0.001	1	
AGP172	*tcer-1;glp-1*	*tcer-1 (Endogenous promoter)*	58/66	44	+/- 3.32	0.0002	0.0001	0.001
			Trial 4[#]			Bonferroni P-value		
N2	Wildtype	Control	83/91	75.3	+/- 2.94			
CF1903	*glp-1*	Control	91/92	102.34	+/- 2.86	0.0001		
CF2154	*tcer-1;glp-1*	Control	59/85	126.59	+/- 6.16	0.001	0.0001	
AGP171	*tcer-1;glp-1*	*Neurons (rgef-1)*	82/102	84.31	+/- 5.42	0.1357	0.9185	0.0001
			Trial 5			Bonferroni P-value		
N2	Wildtype	Control	68/86	71.77	-2.68			
CF1903	*glp-1*	Control	88/99	98.35	-2.99	0.001		
CF2154	*tcer-1;glp-1*	Control	50/80	129.12	-6.11	0.001	0.0001	
AGP171	*tcer-1;glp-1*	*Neurons (rgef-1)*	65/78	90.72	-6.65	0.012	1	0.0008

4C. Survival of strains expressing TCER-1 in different tissues in wildtype animals upon exposure to *P. aeruginosa* PA14

Strain	Genotype	Tissue of TCER-1 expression (promoter)	Trial 1			Bonferroni P-value	
			n = obs/ total	Mean	SE ^	P (*vs* N2)	P (*vs tcer-1*)
N2	Wildtype	Control	66/75	94.25	+/- 2.31		0.0039
CF2166	*tcer-1*	Control	38/50	110.04	+/- 4.07	0.0039	
AGP75	Wildtype	*tcer-1 (Endogenous promoter)*	24/25	83.03	+/- 2.53	0.0156	0.001
AGP76	Wildtype	*Muscle (myo-3)*	41/42	76.92	+/- 3.35	0.0013	0.001
AGP77	Wildtype	*Intestine (gly-19)*	60/80	82.38	+/- 2.33	0.008	0.001
AGP132	Wildtype	*Neurons (rgef-1)*	49/60	75.14	+/- 2.01	0.001	0.001
AGP108	Wildtype	*Hypodermis (col-12)*	61/76	88.84	+/- 2.39	0.7878	0.0001
			Trial 2			Bonferroni P-value	
N2	Wildtype	Control	80/95	83.7	+/- 1.94		0.001
CF2166	*tcer-1*	Control	34/60	115.18	+/- 5.53	0.001	
AGP75	Wildtype	*tcer-1 (Endogenous promoter)*	17/23	81.65	+/- 5.15	1	0.0011
AGP76	Wildtype	*Muscle (myo-3)*	58/67	78.55	+/- 2.35	1	0.001
AGP77	Wildtype	*Intestine (gly-19)*	94/111	83.79	+/- 1.76	1	0.001
AGP132	Wildtype	*Neurons (rgef-1)*	73/83	77.32	+/- 2.4	0.303	0.001
AGP108	Wildtype	*Hypodermis (col-12)*	72/80	85.88	+/- 1.98	1	0.001
			Trial 3			Bonferroni P-value	
N2	Wildtype	Control	66/81	79.56	+/- 1.52		0.001
CF2166	*tcer-1*	Control	46/75	113.11	+/- 2.96	0.001	
AGP75	Wildtype	*tcer-1 (Endogenous promoter)*	51/65	77.01	+/- 2.21	1	0.001
			Trial 4			Bonferroni P-value	
N2	Wildtype	Control	59/73	61.92	+/- 2.4		0.001
CF2166	*tcer-1*	Control	65/77	85.37	+/- 2.7	0.001	
AGP75	Wildtype	*tcer-1 (Endogenous promoter)*	68/80	55.82	+/- 2.24	0.1456	0.001

[#] Data from Table 4A (Trial 1) represented in Fig. 2 (a-e).

[#] Data from Table 4B (Trial 3), (Trial 2) and (Trial 4) represented in Fig. 2(f), Fig. 2(g,l and j) and Fig. 2(h) respectively.

Amrit et al., Supplementary Table 5. Survival of wild type worms and *tcer-1* mutants exposed to *P. aeruginosa* PA14 at different ages

Strain	Genotype	Stage of PA14 Exposure	Trial 1			
			n = obs/ total	Mean (hrs)	SE ^	P (*vs* N2)
N2	Wildtype	L4	84 / 105	85.96	+/- 1.78	
CF2166	*tcer-1*	L4	27 / 90	108.65	+/- 5.69	**0.0001**
N2	Wildtype	Day 2	92 / 100	44.18	+/- 1.97	
CF2166	*tcer-1*	Day 2	64 / 113	65.52	+/- 2.15	**0.001**
N2	Wildtype	Day 4	89 / 101	18.22	+/- 1.84	
CF2166	*tcer-1*	Day 4	101 / 102	17.3	+/- 1.38	**0.8361**
N2	Wildtype	Day 6	84 / 102	41.02	+/- 2.47	
CF2166	*tcer-1*	Day 6	97 / 100	20.42	+/- 0.98	**0.001**
N2	Wildtype	Day 9	91 / 100	25.16	+/- 1.9	
CF2166	*tcer-1*	Day 9	92 / 101	10.5	+/- 0.98	**0.001**
**Trial 2**						
N2	Wildtype	L4	87 / 100	47.63	+/- 4.14	
CF2166	*tcer-1*	L4	53 / 100	84.01	+/- 3.74	**0.0001**
N2	Wildtype	Day 2	77 / 104	46.5	+/- 1.44	
CF2166	*tcer-1*	Day 2	97 / 123	74.5	+/- 2.45	**0.001**
N2	Wildtype	Day 4	85 / 108	28	+/- 0.83	
CF2166	*tcer-1*	Day 4	108 / 125	36.66	+/- 1.48	**0.0001**
N2	Wildtype	Day 6	98 / 105	21.95	+/- 1.19	
CF2166	*tcer-1*	Day 6	87 / 105	15.84	+/- 0.76	**0.0001**
N2	Wildtype	Day 9	93 / 100	13.81	+/- 1.42	
CF2166	*tcer-1*	Day 9	95 / 100	13.01	+/- 0.9	**0.8798**
**Trial 3**						
N2	Wildtype	L4	110 / 120	73.38	+/- 1.62	
CF2166	*tcer-1*	L4	91 / 145	100.18	+/- 2.54	**0.0001**
N2	Wildtype	Day 2	77 / 81	32.35	+/- 1.55	
CF2166	*tcer-1*	Day 2	108 / 113	59.26	+/- 2.29	**0.001**
N2	Wildtype	Day 4	160 / 170	30.59	+/- 1.24	
CF2166	*tcer-1*	Day 4	162 / 183	41.06	+/- 1.57	**0.001**
N2	Wildtype	Day 6	126 / 158	36.98	+/- 1.51	
CF2166	*tcer-1*	Day 6	158 / 179	26.04	+/- 1.25	**0.0001**
N2	Wildtype	Day 9	98 / 98	16.95	+/- 1.32	
CF2166	*tcer-1*	Day 9	146 / 146	16.99	+/- 0.94	**0.8193**
**Trial 4#**						
N2	Wildtype	L4	36 / 100	71.69	+/- 2.39	
CF2166	*tcer-1*	L4	10 / 100	79.97	+/- 3.96	**0.051**
N2	Wildtype	Day 2	59 / 100	40.89	+/- 1.53	
CF2166	*tcer-1*	Day 2	30 / 80	51.36	+/- 1.91	**0.0001**
N2	Wildtype	Day 4	89 / 120	31.41	+/- 1.1	
CF2166	*tcer-1*	Day 4	66 / 76	38.86	+/- 1.56	**0.0001**
N2	Wildtype	Day 6	76 / 102	24.82	+/- 1.32	
CF2166	*tcer-1*	Day 6	99 / 106	26.17	+/- 1.09	**0.3852**
N2	Wildtype	Day 9	84 / 86	20.91	+/- 1.11	
CF2166	*tcer-1*	Day 9	65 / 66	19.64	+/- 1.21	**0.4315**
**Trial 5**						
N2	Wildtype	L4	40 / 117	72.5	+/- 2.21	
CF2166	*tcer-1*	L4	20 / 100	77.54	+/- 1.91	**0.0449**
N2	Wildtype	Day 2	66 / 141	42.79	+/- 1.05	
CF2166	*tcer-1*	Day 2	25 / 145	56.84	+/- 1.91	**0.001**
N2	Wildtype	Day 4	81 / 113	22.41	+/- 1.08	
CF2166	*tcer-1*	Day 4	88 / 118	34.08	+/- 1.52	**0.001**
N2	Wildtype	Day 6	85 / 98	16.95	+/- 1.01	
CF2166	*tcer-1*	Day 6	89 / 95	15.68	+/- 0.39	**0.1052**
N2	Wildtype	Day 9	39 / 50	18.72	+/- 1.69	
CF2166	*tcer-1*	Day 9	26 / 30	17.4	+/- 1.63	**0.4938**
**Trial 6**						
N2	Wildtype	L4	48 / 122	52.58	+/- 1.08	
CF2166	*tcer-1*	L4	12 / 124	77.92	+/- 3.75	**0.001**
N2	Wildtype	Day 2	70 / 123	30.03	+/- 0.85	
CF2166	*tcer-1*	Day 2	60 / 160	43.18	+/- 1.66	**0.001**
N2	Wildtype	Day 4	114 / 144	29.97	+/- 0.9	
CF2166	*tcer-1*	Day 4	130 / 150	31.92	+/- 0.71	**0.0505**
N2	Wildtype	Day 6	136 / 139	12.08	+/- 0.35	
CF2166	*tcer-1*	Day 6	138 / 140	11.85	+/- 0.24	**0.7772**
N2	Wildtype	Day 9	108 / 112	6.67	+/- 0.57	
CF2166	*tcer-1*	Day 9	98 / 103	9.56	+/- 0.81	**0.002**
**Trial 7**						
N2	Wildtype	L4	91 / 108	42.08	+/- 3.84	
CF2166	*tcer-1*	L4	68 / 108	46.7	+/- 4.06	**0.5215**
N2	Wildtype	Day 2	73 / 118	50.5	+/- 1.97	
CF2166	*tcer-1*	Day 2	65 / 112	59.65	+/- 1.74	**0.003**
N2	Wildtype	Day 4	54 / 75	38.91	+/- 1.93	
CF2166	*tcer-1*	Day 4	78 / 101	31.22	+/- 1.64	**0.0023**
N2	Wildtype	Day 6	68 / 71	19	+/- 1.51	
CF2166	*tcer-1*	Day 6	46 / 51	16.97	+/- 2.00	**0.9215**
N2	Wildtype	Day 9	39 / 57	16.24	+/- 2.78	
CF2166	*tcer-1*	Day 9	55 / 58	11.74	+/- 1.66	**0.1904**

[#] Data from Table 5 (Trial 4) represented in Fig. 3a.

Amrit et al., Supplementary Table 6. Impact of TCER-1-repressed genes on *P. aeruginosa* PA14 resistance

6A. Effect of mutations in TCER-1-repressed genes on PA14 resistance induced by *tcer-1* RNAi

Strain	Genotype	RNAi	Trial 1#			Bonferroni P-value		
			n = obs/ total	Mean	SE ^	P (*vs* N2 on Ctrl)	P (*vs* N2 on *tcer-1* RNAi)	P (*vs* mutant on Ctrl)
N2	Wildtype	*Ctrl*	47/65	77.67	+/- 3.01			
N2	*Wildtype*	*tcer-1*	85/101	104.62	+/- 2.68	0.001		
RB2478	*irg-5(ok3418)]*	*Ctrl*	74/90	53.07	+/- 0.71	0.001	0.001	
RB2478	*irg-5(ok3418)]*	*tcer-1*	78/90	56.69	+/- 1.21	0.001	0.001	0.164
			Trial 2#			Bonferroni P-value		
N2	Wildtype	*Ctrl*	67/100	87.63	+/- 2.23			
N2	Wildtype	*tcer-1*	80/105	99.27	+/- 2.05	0.0006		
RB1994	*dod-24(ok2629)*	*Ctrl*	71/90	66.18	+/- 1.42	0.0001	0.0001	
RB1994	*dod-24(ok2629)*	*tcer-1*	88/105	74.20	+/- 1.49	0.0001	0.0001	0.0015
RB2356	*dod-3(ok3202)*	*Ctrl*	74/100	79.95	+/- 1.58	0.0027	0.001	
RB2356	*dod-3(ok3202)*	*tcer-1*	83/100	78.89	+/- 1.45	0.0001	0.001	1
			Trial 3			Bonferroni P-value		
N2	Wildtype	*Ctrl*	68/100	57.47	+/- 3.04			
N2	Wildtype	*tcer-1*	53/105	108.46	+/- 3.91	0.001		
VC2496	*ilys-3(ok3222)*	*Ctrl*	56/103	54.53	+/- 2.07	0.929	0.001	
VC2496	*ilys-3(ok3222)*	*tcer-1*	65/101	55.51	+/- 1.67	0.568	0.001	1
RB1994	*dod-24(ok2629)*	*Ctrl*	36/40	35.15	+/- 3.37	0.0001	0.001	
RB1994	*dod-24(ok2629)*	*tcer-1*	62/77	62.42	+/- 2.33	1	0.0001	0.0001
RB2356	*dod-3(ok3202)*	*Ctrl*	47/70	72.57	+/- 3.5	0.0082	0.001	
RB2356	*dod-3(ok3202)*	*tcer-1*	55/95	62.64	+/- 3.87	0.5273	0.001	0.4335
			Trial 4			Bonferroni P-value		
N2	Wildtype	*Ctrl*	75 / 89	70.93	+/- 1.8			
N2	Wildtype	*tcer-1*	46 / 58	75.99	+/- 1.57	0.2074		
RB2478	*irg-5(ok3418)]*	*Ctrl*	76 / 90	45.96	+/- 2.29	0.001	0.001	
RB2478	*irg-5(ok3418)]*	*tcer-1*	50 / 58	45.68	+/- 2.3	0.001	0.001	1
			Trial 5#			Bonferroni P-value		
N2	Wildtype	*Ctrl*	50/85	72.12	+/- 2.3			
N2	Wildtype	*tcer-1*	59/81	81.33	+/- 2.45	0.0002		
VC2496	*ilys-3(ok3222)*	*Ctrl*	66/103	52.15	+/- 1.94	0.0001	0.0001	
VC2496	*ilys-3(ok3222)*	*tcer-1*	66/100	55.21	+/- 2.03	0.0001	0.0001	1

6B. Survival of *tcer-1;ilys-3* and *tcer-1;dod-3* double mutants upon exposure to PA14

Strain	Genotype	Trial 1#			Bonferroni P-value	
		n = obs/ total	Mean	SE ^	P (*vs* N2)	P (*vs* *tcer-1*)
N2	Wildtype	91 / 119	88.05	+/- 2.08		
CF2166	*tcer-1*	86 / 119	111.81	+/- 2.35	0.001	
VC2496	*ilys-3*	106 / 112	54.29	+/- 1.3	0.001	0.0001
AGP256	*tcer-1;ilys-3*	84 / 89	60.74	+/- 1.54	0.001	0.0001
		Trial 2			Bonferroni P-value	
N2	Wildtype	129 / 150	81.61	+/- 1.68		
CF2166	*tcer-1*	112 / 132	99.18	+/- 2.54	0.0001	
VC2496	*ilys-3*	103 / 113	52.06	+/- 1.81	0.001	0.001
AGP256	*tcer-1;ilys-3*	81 / 93	60.58	+/- 2.05	0.001	0.001

6C. Survival of *dod-3;tcer-1* double mutants upon exposure to *P. aeruginosa* PA14

Strain	Genotype	Trial 1			Corrected P value		
		n = obs/ total	Mean	SE ^	P (*vs* N2)	P (*vs* *tcer-1*)	P (*vs dod-3*)
N2	Wildtype	129 / 156	78.34	1.77			
CF2166	*tcer-1*	70 / 107	103.14	2.16	0.0001		
AGP258	*dod-3*	114 / 147	82.36	1.92	0.4726	0.0001	
AGP257	*tcer-1;dod-3*	97 / 118	90.33	2.01	0.0001	0.0001	0.0137
		Trial 2#			Corrected P value		
N2	Wildtype	139 / 151	60.06	0.86			
CF2166	*tcer-1*	93 / 126	87.90	2.04	0.0001		
AGP258	*dod-3*	120 / 131	70.87	0.75	0.0001	0.0001	
AGP257	*tcer-1;dod-3*	113 / 138	79.06	1.36	0.0001	0.0005	0.0001

Data from Table 6A (Trial 2), (Trial 1) and (Trial 5) represented in Fig. 7(a,c), Fig. 7(b) and Fig. 7(d) respectively.

Data from Tables 6B (Trial 1) and 6C (Trial 2) represented in Figs. 7e and 7f respectively.

**Supplementary Table 7:** Transgenic strains used in this study			
**Strain**	**Genotype**	**Transgene**	**Tissue of TCER-1 expression (promoter)**
AGP75	*N2*	*glmEx15 [Ptcer-1::TCER-1::GFP + Pmyo-2::mCherry]*	endogenous (*tcer-1*)
AGP77	*N2*	*glmEx17 [Pgly-19::TCER-1::GFP + Pmyo-2::mCh]*	intestine
AGP132	*N2*	*glmEx25 [Prgef-1::TCER-1::GFP + Pmyo-2::mCh]*	neurons
AGP76	*N2*	*glmEx16 [Pmyo-3::TCER-1::GFP + Pmyo-2::mCh]*	muscles
AGP108	*N2*	*glmEx18 [Pcol12::tcer-1::GFP and Pmyo-2::mCh]*	hypodermis
AGP173	*tcer-1*	*glmEx15 [Ptcer-1::TCER1::GFP; Pmyo-2::mCherry] injected in CF2166*	endogenous (*tcer-1*)
AGP92	*tcer-1*	*glmEx17 [Pgly-19::TCER-1::GFP + Pmyo-2::mCh] injected in CF2166*	intestine
AGP170	*tcer-1*	*glmEx25 [Prgef-1::TCER-1::GFP + Pmyo-2::mCh] injected in CF2166*	neurons
AGP194	*tcer-1*	*glmEx16 [Pmyo-3::TCER-1::GFP + Pmyo-2::mCh] injected in CF2166*	muscles
AGP120	*tcer-1*	*glmEx18 [Pcol12::tcer-1::GFP and Pmyo-2::mCh] injected in CF2166*	hypodermis
AGP80	*glp-1*	*glmEx15 [Ptcer-1::TCER-1::GFP + Pmyo-2::mCherry] injected in CF1903*	endogenous (*tcer-1*)
AGP78	*glp-1*	*glmEx17 [Pgly-19::TCER-1::GFP + Pmyo-2::mCh] injected in CF1903*	intestine
AGP133	*glp-1*	*glmEx25 [Prgef-1::TCER-1::GFP + Pmyo-2::mCh] injected in CF1903*	neurons
AGP194	*glp-1*	*glmEx16 [Pmyo-3::TCER-1::GFP + Pmyo-2::mCh] injected in CF1903*	muscles
AGP121	*glp-1*	*glmEx18 [Pcol12::tcer-1::GFP and Pmyo-2::mCh] injected in CF1903*	hypodermis
AGP172	*tcer-1;glp-1*	*glmEx15 [Ptcer-1::TCER1::GFP; Pmyo-2::mCherry] injected in CF2154*	endogenous (*tcer-1*)
AGP91	*tcer-1;glp-1*	*glmEx17 [Pgly-19::TCER-1::GFP + Pmyo-2::mCh] injected in CF2154*	intestine
AGP171	*tcer-1;glp-1*	*glmEx25 [Prgef-1::TCER-1::GFP + Pmyo-2::mCh] injected in CF2154*	neurons
AGP79	*tcer-1;glp-1*	*glmEx16 [Pmyo-3::TCER-1::GFP + Pmyo-2::mCh] injected in CF2154*	muscles
AGP122	*tcer-1;glp-1*	*glmEx18 [Pcol12::tcer-1::GFP and Pmyo-2::mCh] injected in CF2154*	hypodermis

**Amrit et al., Supplementary Table 8**: Primers used in this study for Q-PCR assays			
**Gene**	**Cosmid**	**Primer Sequence**	
		**Forward**	**Reverse**
pmk-1	B0218.3	5' TTGATGTATGGTCAGTTGGG 3'	5' GATCGATGTGATCAGATCCAG 3'
dod-3	C24B9.9	5' GTGCATATTGTGGAGCTGC 3'	5' ATAGTTGGTCGGACGTTGG 3'
	F55G11.4	5' GGATCCGTGTATTTGGCTG 3'	5' GTTCACAGTTCCATTATAGATCGAG 3'
irg-5	F35E12.5	5' TCTTACTCTCCTGAACTTGTACC 3'	5' TCGTACTTCTTCACCGCAG 3'
	C14C6.5	5' AAGACCTGCGGATACTGTG 3'	5' ACTTGTCTCTGCAGATTCCT 3'
	C08E8.3	5' TACCTCTGGAGCAACTACG 3'	5' GATGATTTGTTCACTCCAACAG 3'
fbxa-59	T12B5.8	5' TCTCGTGGATCTACTGAGC 3'	5' GACACCGAAACGTAATCCA 3'
	C50F7.5	5' GCTCCTTCTGATCTTGGTC 3'	5' CCTTTGTCTTATGATGGTGATGG 3'
ilys-3	C45G7.3	5' GTGGATATTATCAGATCAAACTCCC 3'	5' CGGCTTCAGTTGTTTCTCC 3'
dod-24	C32H11.12	5' ATTGAATTGCTCCAGAACGA 3'	5' CATTTCTGTTGTCCGTCCC 3'
dod-19	ZK6.10	5' AGAGCAGACAACAACTTCC 3'	5' GGTATTAACGTTTCCTAGTCCT 3'
lys-10	F17E9.11	5' GATATTGAACGTCTTGCATGG 3'	5' TTTCTTCAATGAAAGCACGG 3'
	C49C8.5	5' AACTTCAAGGTTGCTACAAAGG 3'	5' CGAGTAATCAAGACCTATTCCG 3'
spp-18	F27C8.4	5' TGAACACGTCAAGAATCATGC 3'	5' GTATCTTCAATGACGGCATGAG 3'
ilys-2	C45G7.2	5' AGAATCAACTGAAGTTGCCTG 3'	5' CGGTTGTAGTAGTTCTCAACAC 3'
	F20E11.17	5' AGAGTAGTGATCGTTCCGG 3'	5' CTATTGGATCCTTGCATGCC 3'
tcer-1 *	ZK1127.9 *	5' GATCATATTGAAAACTTGGGCCGC 3' *	5' TTATTGCTTTCTGCGATCCCGCTC 3' *
tcer-1	ZK1127.9	5' GAGGTCCTGGAGCATATGG 3'	5' ATCCCGAAAGTCTCTTCAGAC 3'
hsp-6	C37H5.8	5' GAAGATACGAAGACCCAGAGGTTC 3'	5' CAACCTGAGATGGGGAATACACT 3'
atfs-1	ZC376.7	5' CCGATCGAAGACGAATGTTG 3'	5' GAACTCCATTTTTCTTCCC 3'
hif-1	F38A6.3	5' CTCTTTACGACTTGCCTGAC 3'	5' GTGGGCTTCTGTCAATGTG 3'

* Ghazi et al. 2009 [35]
