# Supplementary material for: The longevity-promoting factor, TCER-1, widely represses stress resistance and innate immunity
Source: Nat Commun. 2019 Jul 17;10:3042. doi: 10.1038/s41467-019-10759-z (PMC6637209; doi:10.1038/s41467-019-10759-z)
Supplement: Supplementary file 3 — Description of Additional Supplementary Files [file 41467_2019_10759_MOESM3_ESM.pdf]

Description of Additional Supplementary Files

File name: Supplementary Data 1

Description: The impact of TCER-1 expression in individual tissues on the lifespan of wild-type animals as well as *tcer-1*, *glp-1* and *tcer-1;glp-1* mutants. TCER-1 was driven under control of (1A) *tcer-1* endogenous promoter (1B) intestine-specific promotor (1C) Neuron-specific promotor (1D) muscle-specific promotor and (1E) hypodermis-specific promotor.
