# Supplementary material for: The longevity-promoting factor, TCER-1, widely represses stress resistance and innate immunity
Source: Nat Commun. 2019 Jul 17;10:3042. doi: 10.1038/s41467-019-10759-z (PMC6637209; doi:10.1038/s41467-019-10759-z)
Supplement: Supplementary file 2 — Peer Review File [file 41467_2019_10759_MOESM2_ESM.pdf]

Reviewers' comments:

Reviewer #1 (Remarks to the Author):

Amrit et al. report the characterization of TCER-1, a conserved transcription elongation and splicing factor, which has been previously shown by the authors to be required for lifespan extension in germline-deficient animals. Here, the authors focus in particular on the role of TCER-1 in modulating innate immunity, in particular through inhibition of PMK-1 p38 MAPK, which regulates C. elegans innate immunity. The authors also show that tcer-1 mutants confer resistant to abiotic stressors. Somewhat surprisingly, the authors find that TCER-1 activity in any somatic tissue can suppress the pathogen resistance of tcer-1 mutants. The authors note that pathogen infection results in diminished progeny production, but that TCER-1 overexpression, while conferring enhanced susceptibility to pathogen killing, actually blunts the diminished fertility during infection, suggesting an intriguing interplay between immunity and fertility.

Overall, TCER-1 appears to be an interesting factor that decouples which appears to decouple lifespan and stress resistance. Generally, these two phenotypes are strongly correlated, and thus the observation that tcer-1 mutants are stress and pathogen-resistant is surprising given the prior studies showing that tcer-1 mutants have diminished lifespan in germline-deficient animals. The study will be of interest to investigators in the field of aging and those who are interested in the integrative physiology of aging, immunity, and reproduction.

I have a couple of technical comments:

1) There seems the possibility that there could be a specific genetic interaction between glp-1 and tcer-1. Given the generalizations made by the authors, it would seem prudent to examine if tcer-1 loss behaves the same in at least one other germline-deficient condition/mutant.
2) The results of tissue-specific rescue are surprising, but these rescue experiments involve the interpretation of reduced survival. Might tcer-1 overexpression in tissues be toxic to the animals, without necessarily implying a tissue-specific mutant-rescuing activity?

Reviewer #2 (Remarks to the Author):

I thought this was a fascinating study. The correlation between longevity and stress-resistance is quite a striking one among long-lived mutants and among different species in nature. This correlation has led to the obvious hypothesis that increased resistance to stress (including the slings and arrows of day-to-day living) is the cause of longevity. There have been a number of studies that have broken this correlation, as the authors mention in their paper. For example, FOXO is required for IIS (chico) mutant flies to live long, but not for their increased resistance to oxidative stress. Here, however, we have an even more stunning uncoupling of stress resistance and longevity. In this case, the wild-type function of a gene can increase lifespan while making animals more sensitive to stress. I've never seen this combination before. Moreover, it's not just a single stress (pathogen resistance) but multiple types of stress. These findings really suggests that mechanisms that promote at least these types of stress resistances do not underlie longevity. (In fact, I urge the authors to make this point even more clearly—for example, top of page 20, abstract, intro--of course with the caveat that there may be a type of stress resistance that they have not tested.). Another very interesting finding is the link to reproduction—namely, that TCER-1 only confers stress resistance during the normal reproductive period. (Wonder what would happen in females?) Their interpretation that animals "choose" to devote resources to reproduction at the expense of self-protection is very interesting. It would also help select against genetically "unfit" animals; that is animals who may be more likely to "die during childbirth".
Anyway, it's a very interesting and important study. Maybe even a landmark study.

I have a few suggestions for improving the paper:

1. Leave non-autonomy out of the title. It's not the main point of the paper and it's a bit confusing. In fact, I was wondering whether the TCER-1 gene doesn't really act non-autonomously

at all. Maybe overexpressing in any one tissue makes that tissue susceptible to infection, so that specific tissue dies and takes with it the rest of the animal. Can the authors rule out that possibility?
2. Introduction: the authors may want to say that in yeast, worms and plants, selections for stress resistance have yielded long-lived mutants, but that only a fraction of the stress-resistant mutants are long lived. This incomplete correlation is another argument that stress-resistance alone is not sufficient to extend lifespan. Instead (and they should say this), another process that is induced in long-lived mutants coordinately with increased stress resistance in so many long-lived mutants (though, clearly not tcer-1 mutants) must be causing the longevity.
I would change this sentence: “However, this is typically hierarchical with one tissue (often neurons) spearheading the release of transcellular signals to coordinate the organism- wide response (reviewed in 63, 64).” to say “…(in some cases, neurons)” since there are many cases in which other tissues can exert life extending effects (as with FOXO in flies and worms, muscle-specific gene expression in flies , HSF-1 (Morimoto paper), loss of germ cells, etc. The neural effect may just be better publicized. In fact, the effect of FOXO in neurons seems pretty small….
3. Here: “we noted increased accumulation of TCER-1::GFP in peri-nuclear ‘speckles’ in germline nuclei of infected adults as compared to wild-type worms though this did not achieve statistical significance (Fig. S8b)”, can you power the experiment whether enough to see whether this is really significant? If not, then better not to say it.
4. Finally, and this is a more substantial issue: TCER-1 provides a nice opportunity to ask what aspect of TCER-1 function DOES confer life extension. An obvious candidate would be increased levels of macroautophagy. Has this been examined? Could the authors have a look at macroautophagy, and perhaps knock down some gene required for macroautophagy and see whether that prevents life extension? That information would make this an even nicer paper, but it’s a bit off topic so I would consider this an optional request (but one that I encourage).

5. Minor point: there are a number of grammatical errors, and recurring errors with worm nomenclature.

Reviewer #3 (Remarks to the Author):

The authors describe the role of TCER-1, a transcription elongation and splicing factor, in stress resistance. TCER-1 was previously shown to increase lifespan; here, they show that disruption of tcer-1 leads to increased stress resistance. This is an intriguing result as it demonstrates an uncoupling of longevity and stress resistance-two phenotypes that are typically associated with each other. However, there are two major issues that I have with the manuscript and a multitude of minor issues.

Major issues:
The data suggesting that tcer-1 have an overall increased resistance to stress is compelling. However, the bulk of the data refers to survival to P. aeruginosa (PA14) exposure. For this, the authors claim that the increased resistance to PA14 is not due to reduced feeding. However, this is based on two pieces of evidence, neither of which seem to suggest that there is less PA14 in tcer-1 mutants. It is not clear to me why the authors view these data indicate that the enhanced resistance is not due to less feeding. The results of the CFU assay could be due to either increased resistance of the mutants or decreased ingestion of the bacteria. The authors need to test the number of pharyngeal pumps per minute in WT and tcer-1 in both OP50 and PA14.

This is a data-rich manuscript with numerous survival assays. Given the inherent trial to trial variability in survival experiments, I appreciate the presentation of individual trial results in the supplemental tables. However, I have several issues with the analyses/presentation of the data: First, Figure 3A appears to be an amalgamation of different experiments. Based on Table S6, the authors cherry pick data from different trials for the different developmental stages presented in Fig 3A. The selection of trials gives the appearance of significant differences ($p<0.05$) up to 6 day adults and then switching to $p>0.05$. However, Table S6 indicates that none of the trials were so clear cut. Figure 3B then pools the data from the various trials. This would be reasonable if there weren’t significant differences among the various trials for given treatments. However both WT

and tcer-1 vary substantially across trials and, therefore, pooling the trials may not be appropriate.
Second, some of the control data in various trials presented in Table S5 are surprisingly identical. For example, the WT, glp-1, and tcer-1;glp-1 data for Trial 3 in S5A is identical to Trial 6 in S5B. Were these actually separate trials?
Third, the survival data were analyzed using the log-rank Mantel Cox method. My understanding is that this test is not necessarily robust against data with differing slopes. For example, several of the rescue constructs have survival curves that cross over the WT curve.
Finally, it is not clear if any of the survival tests were subject to a correction for multiple treatment comparisons.

Minor issues:
Ln 46: Should be Introduction??
Ln 53: Evidence doesn't require an 's'.
Ln 56: It's not clear to me why it is important to understand the pathways that uncouple stress and longevity. Wouldn't you want both?
Ln 87 and elsewhere: There are several instances of less than formal scientific nomenclature. For example, using "worms" instead of C. elegans. I would encourage the authors to revisit these.
Ln 112: Gram-positive should be hyphenated. There are many examples of "mis"hypenation.

Ln 161: "knockdown" is typically associated with RNAi, but this experiment used the mutant, correct?
All figures: It would be helpful if statistical significance and/or some indication of variability were included within the graphs.
Figure 1f: I recommend that the data be presented in their raw format (i.e. not relative to untreated). The data should be analyzed (e.g. logistic regression) to estimate the LD50 with confidence intervals for WT and tcer-1.
Ln 201 and Fig. 2a: Based on your analysis, the endogenous rescue line is still significantly more resistant than WT. I, therefore, wouldn't claim that the resistance was abolished. Also, it appears that the endogenous rescue line was only tested once in an experiment with the tcer-1 mutant alone (Table S4A trial 1). This should be repeated.
Ln 217: You state that endogenous tcer-1 rescued lifespan to glp-1 levels. However, according to Table S5, the double tcer-1;glp-1 mutant was only included in one of the three trials (trial 3) and in that trial there was still a statistical difference between the rescue and glp-1 single mutant.
Ln 244: According to Table S4B the p-value was not <0.01 for either trial with the neuronal rescue.
Figure 3C: The result of tcer-1 mutant on egg laying should be shown.
Figures 3d-e: It's not clear why this is shown as % rescue rather than number of egg laid.
Ln 318: Should be "eggs"
Lns 366-369: If the data are not statistically significant at your predetermined level of alpha, then how can we make conclusions based on the trend?
Ln 368: Should be "compared"
Ln 408: Missing a "the"
Ln 473: You state that dod-3, irg-5, dod-24, and ilys-3 have no lifespan extension. In fact, the first three are less resistant to PA14 than N2.
Ln 538: Change homology to either identity or similarity.
Ln 538: Spell out genus name on first usage.
Lns 610-616: It isn't clear why the swimming analysis isn't presented until the discussion. This should be in the results. Also, I don't understand how a reduced swimming ability can be reminiscent of pathogen resistance.
Lns 656-660: It isn't clear why the tcer-1 expression data is left until the discussion. This is important and should be included in the results. Also, I don't really see the speckles mentioned in figure S8B. Either the figures need to be improved remarkably, or quantification needs to be included.
Ln 70: Transgenic misspelled.
Ln 720: Should be "and"
Ln 725: I've never heard of a C. elegans thorax.
Ln 831: Typo.
Ln 844: Why was the pharynx alone used to quantify GFP?

Ln 850: Dissecting is misspelled.
Ln 866: Should be overlapping.
Figure S2a: I don’t understand how the fold change was calculated and it isn’t clear in the methods how this was calculated.
Ln 927: Associated is misspelled.

We are thankful to the reviewers for their insightful comments and valuable suggestions that have greatly improved the manuscript. Following is a brief summary of the main experiments undertaken based on their suggestions, the new data included in the revised manuscript and the conclusions derived from them (italics). The reviewers' comments are listed in the subsequent section (in blue) along with our detailed response to each.

1. **Examined survival on PA14 of strains overexpressing TCER-1 in four individual tissues of wild-type animals** (new Table S4c).
These experiments, along with our previous data, showed that the suppression of tcer-1 mutants' PA14 resistance by TCER-1 expression in different tissues is not simply a byproduct of transgene toxicity.

2. **Compared pharyngeal pumping rates of wild type, *tcer-1* mutants and animals overexpressing TCER-1, on both *E. coli* OP50 and *P. aeruginosa* PA14** (new Fig. S1a).
This experiment showed that the enhanced pathogen resistance of tcer-1 mutants (and higher susceptibility of TCER-1 overexpressors) is not due to differences in rate of food uptake.

3. **Examined the effect of *tcer-1* RNAi on longevity of germline-less *mes-1(bn7)* mutants.**
This experiment provided evidence that TCER-1 promotes the longevity of germline-less animals and not just the glp-1 mutant.

4. **Documented and quantified the effect of age, and PA14 exposure, on TCER-1 levels** (new Figs. 4 and S8).
These experiments showed that TCER-1 expression in both germ cells and intestinal cells is highest during young adulthood and declines with age. PA14 exposure also causes a significant reduction in TCER-1 levels.

5. **Examined additional healthspan measures in *tcer-1* mutants, including (a) thrashing efficiency, and (b) paralysis induction in a *C. elegans* model of Aβ proteotoxicity** (new Figs. 8 and S9).
These experiments provide evidence that, in addition to repressing stress resistance, TCER-1 also represses some physiological measures of healthspan.

6. **Reanalyzed survival data by applying Bonferroni/multiplicity correction to all experiments where more than two strains/conditions were tested.**
Our original results and conclusions were confirmed upon reanalysis.

7. **Generated *tcer-1;dod-3* double mutant and tested its survival on PA14** (new Fig. 7f).
This experiment verified the data obtained by RNAi inactivation of dod-3, and further confirmed that TCER-1-repressed genes contribute to immunoresistance in C. elegans.

8. **Performed additional biological replicates for survival of strains expressing TCER-1 driven by the endogenous and neuronal promoters**.

RESPONSE TO REVIEWERS

Reviewer #1 (Remarks to the Author):

Amrit et al. report the characterization of TCER-1, a conserved transcription elongation and splicing factor, which has been previously shown by the authors to be required for lifespan extension in germline-deficient animals. Here, the authors focus in particular on the role of TCER-1 in modulating innate immunity, in particular through inhibition of PMK-1 p38 MAPK, which regulates *C. elegans* innate immunity. The authors also show that *tcer-1* mutants confer resistant to abiotic stressors. Somewhat surprisingly, the authors find that TCER-1 activity in any somatic tissue can suppress the pathogen resistance of *tcer-1* mutants. The authors note that pathogen infection results in diminished progeny production, but that TCER-1 overexpression, while conferring enhanced susceptibility to pathogen killing, actually blunts the diminished fertility during infection, suggesting an intriguing interplay between immunity and fertility.
Overall, TCER-1 appears to be an interesting factor that decouples which appears to decouple lifespan and stress resistance. Generally, these two phenotypes are strongly correlated, and thus the observation that *tcer-1* mutants are stress and pathogen-resistant is surprising given the prior studies showing that *tcer-1* mutants have diminished lifespan in germline-deficient animals. The study will be of interest to investigators in the field of aging and those who are interested in the integrative physiology of aging, immunity, and reproduction.
We are thankful for the encouraging remarks on this study, and for the valuable suggestions below.

I have a couple of technical comments:

1) There seems the possibility that there could be a specific genetic interaction between *glp-1* and *tcer-1*. Given the generalizations made by the authors, it would seem prudent to examine if *tcer-1* loss behaves the same in at least one other germline-deficient condition/mutant.

We have tested the effect of *tcer-1* RNAi inactivation on another germline-less, long-lived model, the *mes-1(bn7)* mutant (used in [1,2,3] amongst others). As seen below, *tcer-1* RNAi significantly suppressed the extended lifespan of sterile *mes-1* mutants, and its effect was comparable to the effect on the *glp-1* model.

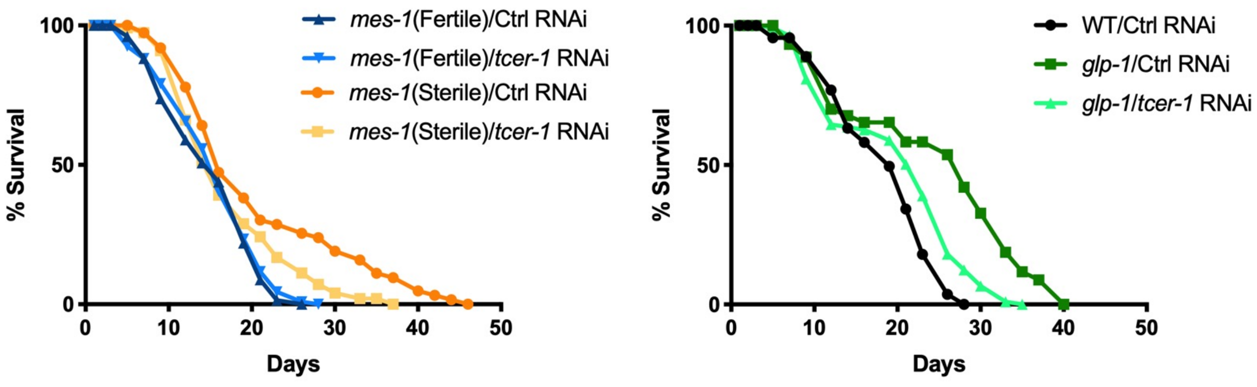

Fig. 1: *tcer-1* RNAi suppresses the lifespan extension in two models of germline-less longevity.
Left Panel: In the *mes-1(bn7)* strain grown at 25^0C, a fraction of progeny is sterile and long-lived, whereas, the rest are fertile and serve as internal controls. Fertile *mes-1* on empty vector RNAi control (Ctrl, dark blue: m = 15.6 ± 0.6, n = 76/106); Fertile *mes-1* on *tcer-1* RNAi (light blue: m = 15.4 ± 0.5, n = 112/123, P vs fertile Ctrl 1); Sterile *mes-1* on empty vector control (orange: m = 20.77 ± 1.2, n = 66/88, P vs fertile Ctrl 0.0009); Sterile *mes-1* on *tcer-1* RNAi (yellow: m = 17.3 ± 0.6, n = 107/119, P vs Sterile Ctrl 0.02).
Right Panel: Wild type N2 strain on Ctrl (WT, black: m = 18.2 ± 0.7, n = 58/80); *glp-1(e2144ts)* on Ctrl (dark green: m = 24.2 ± 1.6, n = 42/45, P vs WT 0.0001); *glp-1(e2144ts)* on *tcer-1* RNAi (light green: m = 19.9 ± 0.7, n = 107/110, P vs. *glp-1* Ctrl 0.004, P vs WT 0.05). P in all cases subjected to Bonferroni correction.

Additionally, in our previous report, demonstrating the role of TCER-1 in promoting the longevity of germline-less *C. elegans*, we showed that **(a)** TCER-1 expression is elevated upon germline elimination obtained by laser ablation of germ-cell precursors, Z2, Z3, as well as by using the *glp-1* model (see Figs. 3A-C in *Ghazi et al., PLoS Gen. 2009*[4]), and, **(b)** TCER-1 overexpression increases the lifespan of wild-type animals, but not of animals subjected to Z2, Z3 ablation (see Fig. S2 in *Ghazi et al., PLoS Gen. 2009*[4]). Reports by others have also confirmed a role for TCER-1 in germline-less longevity (eg., *Boulias and Horvitz, Cell Metabolism, 2012*[32]).

We believe that together these data strongly support a *bona fide* role for TCER-1 in germline-less longevity. Since this article addresses TCER-1's role in stress resistance, we reasoned that these data are not directly relevant to the study, and hence have not included it in the manuscript.

2) The results of tissue-specific rescue are surprising, but these rescue experiments involve the interpretation of reduced survival. Might *tcer-1* overexpression in tissues be toxic to the animals, without necessarily implying a tissue-specific mutant-rescuing activity?

This is a valid (and interesting) possibility. However, our previous data (points 'b' and 'c' below) and additional experiments recently conducted ('a') suggest that this is not the case.

a) We tested the PA14 sensitivity of transgenic strains overexpressing TCER-1 in individual tissues in a wild-type background (included as Table S4C). There was no consistent lifespan alteration. TCER-1 overexpression effects varied from a 2% increase in survival to 20% reduction (in the trial where the reduction was seen, the control wild-type animals survived extraordinarily long rather than the transgenics living shorter). By comparison, these transgenes suppressed *tcer-1* mutants' survival on PA14 by an average ~30-40%. (Table S4A).

b) Transgenes overexpressing TCER-1 in individual somatic tissues of wild-type *C. elegans* do not cause lifespan shortening (or visible sickness) when grown on normal *E. coli* OP50 food (Table S5A-E). For each of three trials conducted for intestinal, neuronal, hypodermal and muscle (2 trials) TCER-1 overexpression (11 experiments total), we observed one instance of ~6% lifespan reduction in the transgenic strain (intestinal expression; WT = 20.4 + 0.8, Tg = 19.2 + 0.6, P = 0.03). By comparison, these transgenic strains showed statistically significant lifespan extension in 4 instances (once for each tissue) and no statistical difference from wild type in 6 experiments. (Trials #1-3 in Table S5).

c) Similarly, when these transgenes were expressed in *tcer-1;glp-1* mutants, on OP50, the transgene carrying animals did not exhibit shorter lifespans than the *tcer-1;glp-1* mutant (or wild-type controls) in any of the 19 experiments conducted (4-6 trials per tissue) (Table S5). Indeed, in 19/20 experiments, they rescued the longevity of *tcer-1;glp-1* mutants and/or non-transgenic control siblings significantly.

We believe these data strongly suggest that the transgene-driven TCER-1 phenotypes are not simply a consequence of toxicity- either on OP50 or on PA14 bacteria. We thank the reviewer for raising this issue, and have discussed it in the Results (Pg. 10, Para 1 and Pg. 12, Para 2) and Discussion (Pg. 20, lines 10-16) sections.

Reviewer #2 (Remarks to the Author):

I thought this was a fascinating study. The correlation between longevity and stress-resistance is quite a striking one among long-lived mutants and among different species in nature. This correlation has led to the obvious hypothesis that increased resistance to stress (including the slings and arrows of day-to-day living) is the cause of longevity. There have been a number of studies that have broken this correlation, as the authors mention in their paper. For example, FOXO is required for IIS (chico) mutant flies to live long, but not for their increased resistance to oxidative stress. Here, however, we have an even more stunning uncoupling of stress resistance and longevity. In this case, the wild-type function of a gene can increase lifespan while making animals more sensitive to stress. I've never seen this combination before. Moreover, it's not just a single stress (pathogen resistance) but multiple types of stress. These findings really suggests that mechanisms that promote at least these types of stress resistances do not underlie longevity. (In fact, I urge the authors to make this point even more clearly–for example, top of page 20, abstract, intro--of course with the caveat that there may be a type of stress resistance that they have not tested). Another very interesting finding is the link to reproduction–namely, that TCER-1 only confers stress resistance during the normal reproductive period. (Wonder what would happen in females?) Their interpretation that animals "choose" to devote resources to reproduction at the expense of self-protection is very interesting. It would also help select against genetically "unfit" animals; that is animals who may be more likely to "die during childbirth".
Anyway, **it's a very interesting and important study. Maybe even a landmark study.**

We are thankful to the referee for highlighting the novelty and importance of this study and for the valuable ideas. Based on the suggestion above, we have increased emphasis on the novelty of *tcer-1* mutants' widespread stress resistance, including in the Introduction (Pg. 3; Para 1, lines 7-11) sections, as well as the Discussion (where we articulate the possibility of an as-yet untested stress paradigm that may be relevant; Pg. 21; Para 1).

I have a few suggestions for improving the paper:
1. Leave non-autonomy out of the title. It's not the main point of the paper and it's a bit confusing. In fact, I was wondering whether the TCER-1 gene doesn't really act non-autonomously at all. Maybe overexpressing in any one tissue makes that tissue susceptible to infection, so that specific tissue dies and takes with it the rest of the animal. Can the authors rule out that possibility?

We have modified the title accordingly. The new title is *"The longevity-promoting factor, TCER-1, widely represses stress resistance and innate immunity"*

The possibility raised by the reviewer, that TCER-1 overexpression causes individual tissues' susceptibility and death, is related to point #2 raised by Reviewer 1 and has been discussed in detail above. Overall, the data enumerated above significantly diminish the possibility that TCER-1-expression phenotypes are due to toxicity/tissue death.

An additional, relevant consideration is the extent of exposure of individual tissues to the pathogen. The intestine comes first, and directly, in contact with the pathogen (there is no data to indicate that tissues such as muscles or neurons encounter PA14), is most widely exposed and for the longest duration. If TCER-1 expression was producing death of individual tissues in the presence of PA14, then one would expect that strains with intestinal TCER-1 expression would show the strongest phenotypes/shortest survival. This is not the case. In *tcer-1* mutants, PA14 resistance is suppressed to similar levels by TCER-1 expression in the intestine (~30 to ~40%), neurons (~30 to ~40%), muscles (~28 to ~34%) and hypodermis (~15 to ~25%) (Table S4A). We believe that the preponderance of evidence suggests that TCER-1 acts in a cell non-autonomous manner to influence

immunity and longevity, though the possibility of toxicity cannot be completely overruled (it has been included in the Discussion, Pg. 20, lines 10-16).

2. Introduction: the authors may want to say that in yeast, worms and plants, selections for stress resistance have yielded long-lived mutants, but that only a fraction of the stress-resistant mutants are long lived. This incomplete correlation is another argument that stress-resistance alone is not sufficient to extend lifespan. Instead (and they should say this), another process that is induced in long-lived mutants coordinately with increased stress resistance in so many long-lived mutants (though, clearly not *tcer-1* mutants) must be causing the longevity.

We have modified the Introduction section to reflect these points. We have also added references that include studies from other species such as yeasts[5] and plants[6, 7] on the uncoupling of stress resistance and longevity (Pg. 3, Para 1).

I would change this sentence: "However, this is typically hierarchical with one tissue (often neurons) spearheading the release of transcellular signals to coordinate the organism- wide response (reviewed in 63, 64)." to say "…(in some cases, neurons)" since there are many cases in which other tissues can exert life extending effects (as with FOXO in flies and worms, muscle-specific gene expression in flies , HSF-1 (Morimoto paper), loss of germ cells, etc. The neural effect may just be better publicized. In fact, the effect of FOXO in neurons seems pretty small….

The sentence has been modified as follows (Pg.19, Para 2 and Pg. 20, Para 1, lines 1-7).

> Cell non-autonomous mechanisms that govern longevity and stress resistance have been demonstrated recently in many contexts. In *C. elegans*, protein-folding imbalance in muscles induces transcellular chaperone signaling that evokes stress responses in intestine and neurons. XBP-1 and retrograde Wnt signaling act in neurons to coordinate the organismal UPR^{mt} and UPR^{ER} responses, whereas, intestinal DAF-16 expression is sufficient to confer longevity in germline-less mutants. Similarly, dFOXO activity in *Drosophila* fat body regulates brain insulin signaling as well as lifespan[8], whereas, Activin disruption in muscles impacts systemic insulin metabolism[9]. In mice, Xbp1s activity in Pomc neurons is sufficient to improve hepatic glucose metabolism and protect against diet-induced obesity[10].

3. Here: "we noted increased accumulation of TCER-1::GFP in peri-nuclear 'speckles' in germline nuclei of infected adults as compared to wild-type worms though this did not achieve statistical significance (Fig. S8b)", can you power the experiment whether enough to see whether this is really significant? If not, then better not to say it.

We recognize the weakness of this claim. To demonstrate that the TCER-1::GFP structures are indeed 'speckles', we need to show co-localization with known speckle-localized proteins. Lacking this, we have eliminated the reference to speckles from the article. We were, however, able to confirm that TCER-1::GFP levels decrease significantly with age, and also upon pathogen infection. These data (and their quantification in intestinal and germ-cell nuclei) is now included as two new figures in the manuscript (Fig. 4 and Fig. S8).

4. Finally, and this is a more substantial issue: TCER-1 provides a nice opportunity to ask what aspect of TCER-1 function DOES confer life extension. An obvious candidate would be increased levels of macroautophagy. Has this been examined? Could the authors have a look at macroautophagy, and perhaps knock down some gene required for macroautophagy and see whether that prevents life extension? That information would make this an even nicer paper, but it's a bit off topic so I would consider this an optional request (but one that I encourage).

We agree that TCER-1's lifespan-promoting function is a highly interesting and significant topic. In our previous studies, we have found that TCER-1 (along with the transcription factors, DAF-16 and NHR-49) promotes longevity upon germline elimination by coordinately up-regulating genes involved in multiple lipid biosynthetic and degradative pathways (*Amrit et al., PLoS Gen, 2016; Ratnappan et al., PLoS Gen, 2014*[11, 12]). Other studies have reported the enhancement of autophagy in germline-less animals (dependent upon PHA-4 and HLH-30, but independent of DAF-16) and autophagy-gene inactivation suppresses germline-less longevity[2, 13]. Our genomic approaches, have not revealed any (macro)autophagy genes as potential TCER-1 targets that may extend lifespan, but this is an open and exciting possibility that we wish to pursue further. However, since the 'anti-stress resistance' (and not 'pro-longevity') functions of TCER-1 are the focus of this study, we believe it would have been off-topic here so we have not explored this avenue for the current article. We greatly appreciate the reviewer drawing our attention to this point.

5. Minor point: there are a number of grammatical errors, and recurring errors with worm nomenclature.

We apologize for the errors and have corrected the ones we found upon several careful perusals.

Reviewer #3 (Remarks to the Author):

The authors describe the role of TCER-1, a transcription elongation and splicing factor, in stress resistance. TCER-1 was previously shown to increase lifespan; here, they show that disruption of *tcer-1* leads to increased stress resistance. This is an intriguing result as it demonstrates an uncoupling of longevity and stress resistance-two phenotypes that are typically associated with each other. However, there are two major issues that I have with the manuscript and a multitude of minor issues.

Major issues:

1. The data suggesting that *tcer-1* have an overall increased resistance to stress is compelling. However, the bulk of the data refers to survival to P. aeruginosa (PA14) exposure. For this, the authors claim that the increased resistance to PA14 is not due to reduced feeding. However, this is based on two pieces of evidence, neither of which seem to suggest that there is less PA14 in *tcer-1* mutants. It is not clear to me why the authors view these data indicate that the enhanced resistance is not due to less feeding. The results of the CFU assay could be due to either increased resistance of the mutants or decreased ingestion of the bacteria. The authors need to test the number of pharyngeal pumps per minute in WT and *tcer-1* in both OP50 and PA14.

We thank the reviewer for the suggestion. We have measured the pharyngeal pumping rates on OP50 and PA14, of wild-type animals, *tcer-1* mutants and also a strain overexpressing TCER-1 under its endogenous promoter (that exhibits reduced survival on PA14). As can be seen from the results (Fig. 2), there is no statistically significant difference between the three strains' pumping rates on either OP50 or PA14. Thus, neither the increased survival of *tcer-1* mutants nor the reduced survival of TCER-1 overexpressors,

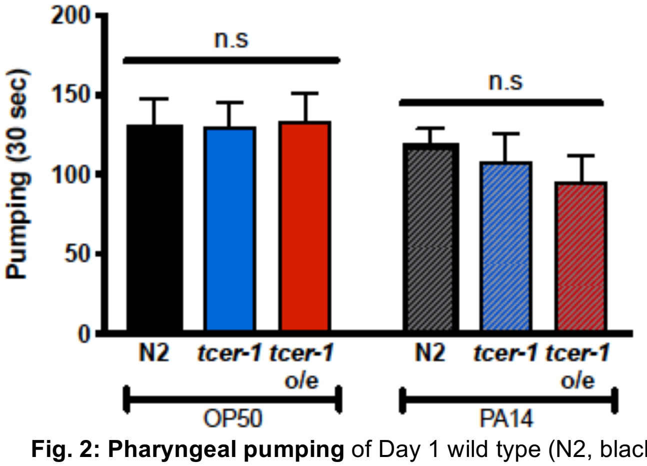

Fig. 2: Pharyngeal pumping of Day 1 wild type (N2, black) adults compared to age-matched *tcer-1* mutants (blue) and worms overexpressing TCER-1 (red) on *E. coli* OP50 (left) and *P. aeruginosa* PA14 exposure (right). No statistical difference was observed between any strains/conditions in an ANOVA test. Data combined from 3 independent biological replicates, each with 15-20 worms per strain.

compared to wild type, can be attributed to differential pathogen ingestion. This data is included in the manuscript as Fig. S2a.

We acknowledge the limitations of the CFU assay however, it is the best approximation for *P. aeruginosa* accumulation in the intestine of *C. elegans*, and is used extensively in the field to assess pathogen resistance. Five such studies are included in the accompanying bibliography[14, 15, 16, 17, 18].

Altogether, we believe these data are sufficient to reasonably conclude that the increased survival of *tcer-1* mutants is due to higher resistance against PA14.

2. This is a data-rich manuscript with numerous survival assays. Given the inherent trial to trial variability in survival experiments, I appreciate the presentation of individual trial results in the supplemental tables. However, I have several issues with the analyses/presentation of the data: First, Figure 3A appears to be an amalgamation of different experiments. Based on Table S6, the authors cherry pick data from different trials for the different developmental stages presented in Fig 3A. The selection of trials gives the appearance of significant differences (p<0.05) up to 6 day adults and then switching to p>0.05. However, Table S6 indicates that none of the trials were so clear cut. Figure 3B then pools the data from the various trials. This would be reasonable if there weren't significant differences among the various trials for given treatments. However, both WT and *tcer-1* vary substantially across trials and, therefore, pooling the trials may not be appropriate.

For each age, we compared the survival of *tcer-1* mutants and wild-type animals on PA14 in 7 independent trials. In part, this was to avoid any confounding effects that may be caused by the variability exhibited by the strains (wild-type variability is discussed below).

The survival curves in Fig. 3a are representative images. The combined results of 7 trials showed that L4, Day 2 and Day 4 mutants survive longer than wild type in a statistically significant manner in 6/7, 7/7 and 6/7 trials, respectively. Whereas, Day 6 and Day 9 mutants survived longer than wild type in 0/7 trials and 1/7 trials. Indeed, Day 6 mutants were shorter lived than wild type in a statistically significant manner in 3 trials. Hence, while the representative images in Fig. 3A are from different trials, we believe these data are convincing evidence to merit the conclusion that *tcer-1* mutants' enhanced PA14 resistance is restricted to young adulthood and does not extend to Day 6 and older animals.

Wild-Type Variability: The variability in survival exhibited by wild-type *C. elegans* on PA14 is well documented. For instance, in Tan et. al. (1999)[19] and Adonizio et. al. (2008)[20], the mean survival is <50 hrs whereas other studies by Feinbaum et. al (2012)[21], Reddy et.al (2009)[22] and Zrieq et. al (2015)[23] have reported survival of over 50h, and in certain instances going up to 90h. The mean survival of the wild type strain in our experiments is shown here in Fig. 3 and, as can be seen, is well within the reported range- a mean survival of ~ 60hr post-exposure.

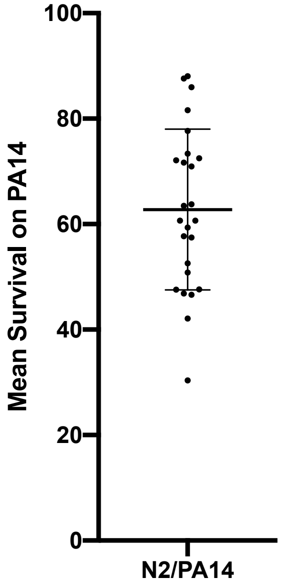

Fig. 3: Variability in survival of wild-type (N2) *C. elegans* exposed to *P. aeruginosa* PA14 (PA14). Data compiled from 25 independent trials undertaken during this study. Y axis shows mean survival in hours upon exposure at the L4 stage.

Our data have also been subjected to Grubb's test outlier analysis (https://www.graphpad.com/quickcalcs/Grubbs1.cfm) to eliminate significant outliers and enhance statistical rigor. The results hold true.

Finally, we would like to respectfully state that in the light of the high variability shown in these assays, it is especially important to

retain Figure 3b in the main figure as this panel (along with Table S6) presents a more accurate picture of the data.

Second, some of the control data in various trials presented in Table S5 are surprisingly identical. For example, the WT, *glp-1*, and *tcer-1;glp-1* data for Trial 3 in S5A is identical to Trial 6 in S5B. Were these actually separate trials?

The experiments in Table S5 examined the effect of TCER-1 expression driven by 5 different promoters (endogenous, muscle, intestine, neuron and hypodermis), each in 4 genetic backgrounds (wild type, *tcer-1*, *tcer-1;glp-1* and *glp-1*) and with each strains' non-transgenic sibling as internal controls for the transgenes. It was not possible to set up and complete all the (~50) lifespans simultaneously. So, we grouped the strains such that the lifespans of the different transgenic strains in 'fertile' genetic backgrounds (wild type, *tcer-1*) were conducted simultaneously, whereas, those in the 'sterile' genetic backgrounds (*glp-1*, *tcer-1;glp-1*) were conducted together. Wild-type control strains were tested in every trial. *This grouping is the reason for the ostensible duplication of the controls, but it was necessary so we could simultaneously compare the effect of TCER-1 overexpression in different tissues in the same genetic background.* As can be seen from Table S5, each of the transgenes' effect was indeed tested in at least 3 and up to 5 separate trials.

Third, the survival data were analyzed using the log-rank Mantel Cox method. My understanding is that this test is not necessarily robust against data with differing slopes. For example, several of the rescue constructs have survival curves that cross over the WT curve.

The log-rank Mantel Cox is the standard test used in the aging and immunity fields for analysis of survival data. Admittedly, it is not ideal when curves of two populations overlap at lifespan mid-points, but it is the best option available to the field. We did not use the alternative, the Gehan-Beslow-Wilcoxon test, because it requires that one group consistently have a higher risk than the other (this would have been an incorrect, misleading assumption on our part) and can be erroneous if censoring is high (as is seen in *C. elegans* survival assays). Due to these reasons, and to maintain uniformity with the field/allow comparisons with other studies, we believe that the Mantel Cox is the appropriate method for this study.

Finally, it is not clear if any of the survival tests were subject to a correction for multiple treatment comparisons.

We thank the reviewer for the suggestion. In all survival assays that involved comparisons of more than 2 strains, we have performed a multiple treatment/Bonferroni correction to the statistical analyses, and this information presented in Supplementary Tables S1, S3, S4, S5 and S7.

In few cases, the Bonferroni P value was different from the previous version such that the difference between controls could not be confirmed in a given trial (eg., S1B, Trial 1) or the rescue by a transgene became statistically insignificant (eg., S4B, Trial 2). Such cases have been highlighted in the supplementary tables as pink boxes/cells. As can be seen, the results of an overwhelming majority of our analyses remained the same and our major conclusions were not altered.

Minor issues:

Ln 46: Should be Introduction??

The sub-title was not included as per the authors' instructions for *Nature Communications*.

Ln 53: Evidence doesn't require an 's'.

This has been corrected (Pg. 2, line 2).

Ln 56: It's not clear to me why it is important to understand the pathways that uncouple stress and longevity. Wouldn't you want both?

Stress resistance and longevity are such closely linked traits that, historically, they have been considered interchangeable. However, there have been intermittent evidences in literature suggesting that they can be uncoupled. Our study is novel and important as it demonstrates how unequivocally these processes can be separated, and because it identifies a gene involved in this uncoupling. Understanding what distinguishes the 'length of life' from measures of 'quality of life' is especially critical because an important goal of the aging field is to proportionally increase the healthy years of life, compared to overall lifespan. It is also significant from a public-health perspective as a rapidly rising population of the elderly poses major biomedical challenges globally.

Ln 87 and elsewhere: There are several instances of less than formal scientific nomenclature. For example, using "worms" instead of *C. elegans*. I would encourage the authors to revisit these.

We have replaced 'worms' with '*C. elegans*' or 'animals' or 'strain', as applicable, throughout the manuscript. We have scrutinized the manuscript carefully to detect and replace inappropriate nomenclature.

Ln 112: Gram-positive should be hyphenated. There are many examples of "mis"hypenation.

Gram-positive has been corrected (Pg. 8, line 6) as well as other examples of 'mis' hyphenation that we detected.

Ln 161: "knockdown" is typically associated with RNAi, but this experiment used the mutant, correct?

Yes. The sentence has been altered to make this clear (Pg. 8, Para 2, last sentence).

All figures: It would be helpful if statistical significance and/or some indication of variability were included within the graphs.

All the main figures in the manuscript now include asterisks indicating the significance of statistical comparisons on the panels. The location and color of the asterisks indicate the strains/conditions being compared.

Figure 1f: I recommend that the data be presented in their raw format (i.e. not relative to untreated). The data should be analyzed (e.g. logistic regression) to estimate the LD50 with confidence intervals for WT and *tcer-1*.

We have employed the method for determining sensitivity to gamma irradiation that is standard in the field. The viability of the strain is normalized to 100% in order to compare the effects of the treatment on wild type versus strain being tested. LD50s are not typically calculated for ionizing radiation because the response is not linear (there are checkpoints that are activated at different thresholds of double-strand breaks). The doses used in our study are also standard in the field for these experiments. Some recent studies where the same methods have been used are included in the bibliography[24, 25, 26, 27] here.

Ln 201 and Fig. 2a: Based on your analysis, the endogenous rescue line is still significantly more resistant than WT. I, therefore, wouldn't claim that the resistance was abolished. Also, it appears that the endogenous rescue line was only tested once in an experiment with the *tcer-1* mutant alone (Table S4A trial 1). This should be repeated.

We have conducted two additional trials with TCER-1 driven by its endogenous promoter in *tcer-1* mutants and find that the reviewer's comment is correct. The rescue in each case is significant but not complete. We have modified the Results section appropriately to reflect this (Pg. 9, Para 1) and the data from the new trials is included in Table S4A.

Ln 217: You state that endogenous *tcer-1* rescued lifespan to glp-1 levels. However, according to Table S5, the double *tcer-1;glp-1* mutant was only included in one of the three trials (trial 3) and in that trial there was still a statistical difference between the rescue and *glp-1* single mutant.

In trials 1 and 2, the non-transgenic sibling of the rescue strains, AGP172 (*tcer-1;glp-1*; *Ptcer-1::tcer-1::GFP*;*Pmyo-2::mCherry*), were used as the controls (as these would be *tcer-1;glp-1* mutants). In both trials, these non-transgenic, controls (a) had lifespans that were statistically indistinguishable from the wild-type, and (b) lived significantly shorter than the transgenic rescue siblings. Importantly, in all three trials, there was no statistically significant difference between the mean lifespans of *glp-1* and the (transgene-carrying) rescue strain following Bonferroni correction, supporting our statement. We have also added data from an additional trial in which the *tcer-1;glp-1* control was assessed, along with the non-transgenic siblings of AGP172 (Trial 4, Table S5A). Here, the rescue strains' lifespan was in fact slightly longer than *glp-1*. The data from all the trials together provides sufficiently strong basis to conclude that the rescue by endogenous promoter-driven TCER-1 was to *glp-1* levels.

Ln 244: According to Table S4B the p-value was not <0.01 for either trial with the neuronal rescue.

We recognize the limitation of the two trials referred to here, because in both cases the control *tcer-1;glp-1* strain did not show a statistically significant increase in survival compared to *glp-1* (although it was significantly longer lived than wild-type in all cases, whereas, the rescue strain was not).
We have performed two additional trials of this experiment. In both cases, we found that the mean survival of the neuronal-rescue transgenics was lower than *tcer-1;glp-1* and *glp-1*, with high statistical significance, and close to the wild-type. This data has been added as Trials 4 and 5 to Table S4B.

Figure 3C: The result of *tcer-1* mutant on egg laying should be shown.

The effect of *tcer-1* mutation on egg laying, fertility, germline development and reproductive aging has been documented in detail in our previous publication (Fig. 7; Fig. S4; *Amrit et al., PLoS Gen. 2016*[11]).

Figures 3d-e: It's not clear why this is shown as % rescue rather than number of egg laid.

Representing the data as % rescue of egg-laying capacity was critical in this case to control for differences in overall brood sizes between strains. Otherwise, we risked an inaccurate inflation in the rescue observed.

Ln 318: Should be "eggs"

The error has been corrected.

Lns 366-369: If the data are not statistically significant at your predetermined level of alpha, then how can we make conclusions based on the trend?

We are not sure which conclusion/sentence is referred to here. We have re-examined the manuscript to ensure that any conclusions derived from the study are supported by statistics.

Ln 368: Should be "compared"

The error has been corrected.

Ln 408: Missing a "the"

The error has been corrected.

Ln 473: You state that dod-3, irg-5, dod-24, and ilys-3 have no lifespan extension. In fact, the first three are less resistant to PA14 than N2.

In this experiment, we were examining the effect of *tcer-1* RNAi inactivation on the survival of *dod-3*, *irg-5*, *dod-24* or *ilys-3* mutants on PA14, *as compared to the effect of the same treatment on wild-type animals*. *tcer-1* RNAi consistently increased survival of PA14-exposed wild type animals. But, it does not elicit increased survival in any of these mutants. The mutants indeed have shorter lifespan on PA14 compared to wild type (an expected observation as these genes are required for immunity), but the noteworthy observation is that they derive no survival benefits upon *tcer-1* RNAi inactivation suggesting that *tcer-1* mutants' enhanced PA14 survival can be attributed, in part, to the activity of these genes.

Ln 538: Change homology to either identity or similarity.

The error has been corrected (Pg. 19, line 6).

Ln 538: Spell out genus name on first usage.

The error has been corrected (Pg. 19, line 6).

Lns 610-616: It isn't clear why the swimming analysis isn't presented until the discussion. This should be in the results. Also, I don't understand how a reduced swimming ability can be reminiscent of pathogen resistance.

We have included a new section under Results (Pg. 16), and new figures (Figs. 8 and S9), showing the effect of *tcer-1* mutation on swimming and thrashing behavior. In the sentence drawing parallels between swimming ability and pathogen resistance, we referred to the fact that post-reproductive, Day 6 *tcer-1* mutants exhibited reduced pathogen resistance as well as reduced swimming ability suggesting a decline in stress resistance as well as healthspan. To avoid confusion, this sentence has been deleted.

Lns 656-660: It isn't clear why the *tcer-1* expression data is left until the discussion. This is important and should be included in the results. Also, I don't really see the speckles mentioned in figure S8B. Either the figures need to be improved remarkably, or quantification needs to be included.

This concern is similar to the one raised by Reviewer 2 (point #3 above). As mentioned above, we agree that the mention of 'speckles' needs stronger confirmatory evidence than we are currently able to provide, so this has been eliminated from the manuscript. We have quantified the data regarding change in levels of TCER-1 expression with age and upon pathogen exposure, and as suggested, this has been included in the Results section (Pg. 13, lines 3-12) and as a main figure (Fig. 4).

Ln 70: Transgenic misspelled.

The error has been corrected.

Ln 720: Should be "and"

The error has been corrected.

Ln 725: I've never heard of a *C. elegans* thorax.

We have replaced 'thorax' with 'pharynx' in the manuscript.

Ln 831: Typo.

The error has been corrected.

Ln 844: Why was the pharynx alone used to quantify GFP?

The pharyngeal grinder efficiently disrupts OP50 leaving essentially no intact bacterial cells within the intestinal lumen. However, in the case of more virulent bacterial strains, extended exposure has been shown to result in accumulation in both the pharynx and the intestine of the animal. In our studies we exposed animals for a short period (24h) before imaging for bacterial accumulation in the pharynx. This region is most suited for GFP quantification given our exposure time and the fact that it is the first site in the digestive tract that the bacteria encounter, and where their 'grinding' commences. While quantification in the intestine can also be used, a fraction of bacteria may be dead and hence not fluorescing by the time they enter the intestine and this can skew the results. Pharyngeal GFP quantification has been used as a standard in the *C. elegans* immunity studies, three of which are listed here[15, 28, 29].

Ln 850: Dissecting is misspelled.

The error has been corrected (Pg. 33, line 4).

Ln 866: Should be overlapping.

The error has been corrected (Pg. 34, line 16).

Figure S2a: I don't understand how the fold change was calculated and it isn't clear in the methods how this was calculated.

The corrected total cell fluorescence (CTCF) value for each condition/treatment we tested was calculated using the Fuji (ImageJ) software, as is often undertaken for such studies[30, 31]. The average CTCF value obtained for WT animals fed GFP-labelled OP50 was then used as the reference to which all the other conditions were compared. We have expanded the relevant Methods section to include this explanation (Pg. 32).

Ln 927: Associated is misspelled.

The error has been corrected (Pg. 52, line 4).

REFERENCES

1. Arantes-Oliveira N, Apfeld J, Dillin A, Kenyon C. Regulation of life-span by germ-line stem cells in Caenorhabditis elegans. *Science* **295**, 502-505 (2002).

2. Lapierre LR, Gelino S, Melendez A, Hansen M. Autophagy and lipid metabolism coordinately modulate life span in germline-less C. elegans. *Curr Biol* **21**, 1507-1514 (2011).

3. Libina N, Berman JR, Kenyon C. Tissue-specific activities of C. elegans DAF-16 in the regulation of lifespan. *Cell* **115**, 489-502 (2003).

4. Ghazi A, Henis-Korenblit S, Kenyon C. A transcription elongation factor that links signals from the reproductive system to lifespan extension in Caenorhabditis elegans. *PLoS genetics* **5**, e1000639 (2009).

5. Fabrizio P, Pozza F, Pletcher SD, Gendron CM, Longo VD. Regulation of longevity and stress resistance by Sch9 in yeast. *Science* **292**, 288-290 (2001).

6. Hou Y*, et al.* The Persimmon 9-lipoxygenase Gene DkLOX3 Plays Positive Roles in Both Promoting Senescence and Enhancing Tolerance to Abiotic Stress. *Front Plant Sci* **6**, 1073 (2015).

7. Kurepa J, Smalle J, Van Montagu M, Inze D. Oxidative stress tolerance and longevity in Arabidopsis: the late-flowering mutant gigantea is tolerant to paraquat. *Plant J* **14**, 759-764 (1998).

8. Hwangbo DS, Gershman B, Tu MP, Palmer M, Tatar M. Drosophila dFOXO controls lifespan and regulates insulin signalling in brain and fat body. *Nature* **429**, 562-566 (2004).

9. Bai H, Kang P, Hernandez AM, Tatar M. Activin signaling targeted by insulin/dFOXO regulates aging and muscle proteostasis in Drosophila. *PLoS genetics* **9**, e1003941 (2013).

10. Williams KW*, et al.* Xbp1s in Pomc neurons connects ER stress with energy balance and glucose homeostasis. *Cell Metab* **20**, 471-482 (2014).

11. Amrit FR*, et al.* DAF-16 and TCER-1 Facilitate Adaptation to Germline Loss by Restoring Lipid Homeostasis and Repressing Reproductive Physiology in C. elegans. *PLoS genetics* **12**, e1005788 (2016).

12. Ratnappan R*, et al.* Germline signals deploy NHR-49 to modulate fatty-acid beta-oxidation and desaturation in somatic tissues of C. elegans. *PLoS genetics* **10**, e1004829 (2014).

13. Lapierre LR*, et al.* The TFEB orthologue HLH-30 regulates autophagy and modulates longevity in Caenorhabditis elegans. *Nat Commun* **4**, 2267 (2013).

14. Yu Y, Zhi L, Wu Q, Jing L, Wang D. NPR-9 regulates the innate immune response in Caenorhabditis elegans by antagonizing the activity of AIB interneurons. *Cell Mol Immunol*, (2016).

15. Zhi L, Yu Y, Li X, Wang D, Wang D. Molecular Control of Innate Immune Response to Pseudomonas aeruginosa Infection by Intestinal let-7 in Caenorhabditis elegans. *PLoS Pathog* **13**, e1006152 (2017).

16. Zou CG, Ma YC, Dai LL, Zhang KQ. Autophagy protects C. elegans against necrosis during Pseudomonas aeruginosa infection. *Proc Natl Acad Sci U S A* **111**, 12480-12485 (2014).

17. Kawli T, Tan MW. Neuroendocrine signals modulate the innate immunity of Caenorhabditis elegans through insulin signaling. *Nat Immunol* **9**, 1415-1424 (2008).

18. Evans EA, Chen WC, Tan MW. The DAF-2 insulin-like signaling pathway independently regulates aging and immunity in C. elegans. *Aging Cell* **7**, 879-893 (2008).

19. Tan MW, Mahajan-Miklos S, Ausubel FM. Killing of Caenorhabditis elegans by Pseudomonas aeruginosa used to model mammalian bacterial pathogenesis. *Proc Natl Acad Sci U S A* **96**, 715-720 (1999).

20. Adonizio A, Leal SM, Jr., Ausubel FM, Mathee K. Attenuation of Pseudomonas aeruginosa virulence by medicinal plants in a Caenorhabditis elegans model system. *J Med Microbiol* **57**, 809-813 (2008).

21. Feinbaum RL*, et al.* Genome-wide identification of Pseudomonas aeruginosa virulence-related genes using a Caenorhabditis elegans infection model. *PLoS Pathog* **8**, e1002813 (2012).

22. Reddy KC, Andersen EC, Kruglyak L, Kim DH. A polymorphism in npr-1 is a behavioral determinant of pathogen susceptibility in C. elegans. *Science* **323**, 382-384 (2009).

23. Zrieq R*, et al.* Genome-wide Screen of Pseudomonas aeruginosa in Saccharomyces cerevisiae Identifies New Virulence Factors. *Front Cell Infect Microbiol* **5**, 81 (2015).

24. Amendola PG, Zaghet N, Ramalho JJ, Vilstrup Johansen J, Boxem M, Salcini AE. JMJD-5/KDM8 regulates H3K36me2 and is required for late steps of homologous recombination and genome integrity. *PLoS genetics* **13**, e1006632 (2017).

25. Ward JD, Barber LJ, Petalcorin MI, Yanowitz J, Boulton SJ. Replication blocking lesions present a unique substrate for homologous recombination. *EMBO J* **26**, 3384-3396 (2007).

26. McClendon TB, Sullivan MR, Bernstein KA, Yanowitz JL. Promotion of Homologous Recombination by SWS-1 in Complex with RAD-51 Paralogs in Caenorhabditis elegans. *Genetics* **203**, 133-145 (2016).

27. Gonzalez-Huici V, Wang B, Gartner A. A Role for the Nonsense-Mediated mRNA Decay Pathway in Maintaining Genome Stability in Caenorhabditis elegans. *Genetics* **206**, 1853-1864 (2017).

28. Wareham DW, Papakonstantinopoulou A, Curtis MA. The Pseudomonas aeruginosa PA14 type III secretion system is expressed but not essential to virulence in the Caenorhabditis elegans-P. aeruginosa pathogenicity model. *FEMS Microbiol Lett* **242**, 209-216 (2005).

29. Kim DH*, et al.* A conserved p38 MAP kinase pathway in Caenorhabditis elegans innate immunity. *Science* **297**, 623-626 (2002).

30. Fernandez-Cardenas LP, Villanueva-Chimal E, Salinas LS, Jose-Nunez C, Tuena de Gomez Puyou M, Navarro RE. Caenorhabditis elegans ATPase inhibitor factor 1 (IF1) MAI-2 preserves the mitochondrial membrane potential (Deltapsim) and is important to induce germ cell apoptosis. *PLoS One* **12**, e0181984 (2017).

31. Dalton HM, Curran SP. Hypodermal responses to protein synthesis inhibition induce systemic developmental arrest and AMPK-dependent survival in Caenorhabditis elegans. *PLoS genetics* **14**, e1007520 (2018).

32. Boulias K, Horvitz HR. The C. elegans microRNA mir-71 acts in neurons to promote germline-mediated longevity through regulation of DAF-16/FOXO. *Cell Metab.* **15(4)**:439-50. (2012).

Reviewers' comments:

Reviewer #1 (Remarks to the Author):

The authors have addressed my concerns satisfactorily. A couple of additional discussion points may be informative:
1. Might the age-dependent decline in PA14 resistance of tcer-1 mutants by day 6 be related to the corresponding decline PMK-1 activity reported by Youngman et al. (2011)?
2. The noted relationship between fecundity and immunity may benefit from further discussion in the context of Miyata et al. (2008), which may be of relevance to the claims in the revised manuscript.

Reviewer #2 (Remarks to the Author):

I have re-reviewed the study, and I am happy with the authors' response to my concerns. It's a very interesting, nice study.

Reviewer #3 (Remarks to the Author):

Review of Amrit et al revision.

While the authors have addressed many of my comments, several regarding data presentation and analysis were left unchanged.

Figure 3A remains unchanged from the original submission. This figure shows the results of experiments testing the tcer-1 mutant and WT for survival following transfer to P. aeruginosa. Rather than showing the results from a single trial, figure 3B uses data for time points from various trials. However, only one out of seven trials gave a result where statistical significance was found at L4, Day 2, and Day 4 with no statistical significance at Days 6 and 9. Based on the original data presented in Table S6, the results are not nearly as clean cut as that presented in Figure 3A. I view this presentation as misleading. In their rebuttal, the authors acknowledge that this is “representative” data; however, this is not mentioned at all in the figure legend. A reader who does not explore the supplementary table will be given the impression that this trend is representative of the population. I recommend either removing figure 3A, changing such that the results from a single trial are presented, or adding a clear caveat to the figure legend that the various graphs are not from the same trial and to look at Fig S6.

Figure 3B remains unchanged from the original submission. It is still not clear whether it is appropriate to pool the data from the various trials. An alternative is to develop a statistical model that includes trial as a block effect. Perhaps a more laborious approach would be to use a series of comparisons between each of the trials for WT and ensure that they are not statistically different. Then do the same for tcer-1. If there are no differences among trials then proceed with pooling the data for figure 3B.

I am alarmed by the inclusion of outlier removal in the revision and mentioned in the rebuttal. The statistical analysis section of the methods now mentions that this was done “when applicable”. It is not clear where this was applicable. In general, I only recommend removing statistical outliers when there was an experimental or data entry error that would suggest the datum is not representative of the overall population variability.

Table S5 appears unchanged from the original submission. I was confused by the author’s rebuttal:
“we grouped the strains such that the lifespans of the different transgenic strains in ‘fertile’ genetic backgrounds (wild type, tcer-1) were conducted simultaneously, whereas, those in the ‘sterile’ genetic backgrounds (glp-1, tcer-1;glp-1) were conducted together. Wild-type control strains were tested in every trial. This grouping is the reason for the ostensible duplication of the controls”
The first sentence suggests that N2 wild type was not tested in every trial; however, the second

sentence suggests that they were included. I, therefore, don't understand from this whether, for example, the 48 N2 animals mentioned in Row 24 of S5A are a different set than those mentioned in Row 56 of S5B? If the data were from different dates (and just by chance got the same result), then I think this table is fine. If, however, they are the same individuals, then presenting them separately seems disingenuous.

The authors recognize my critique of the Mantel Cox method, but suggest that it is the best option available to the field. I would argue that the best statistical test is the one that most likely detects true differences in the populations. It is possible that the Mantel-Cox method will detect those differences; however, if the assumptions for a statistical test are violated substantially then I don't see the logic in using it just because it is commonly used in the field. Were the assumptions tested? Much of the data show very clear differences without statistical analysis; however, if a formal analysis is included, it should be appropriate. Similar to my concern above about pooling data, there may be a more complex statistical models that will better for analyzing these data. An alternative is to not conduct a statistical test, but rather show the means with 95% confidence intervals at each time point.

Detailed Response to Reviewers' Comments

(Reviewers' Comments in Blue)

Reviewer #1

The authors have addressed my concerns satisfactorily. A couple of additional discussion points may be informative:

1. Might the age-dependent decline in PA14 resistance of tcer-1 mutants by day 6 be related to the corresponding decline PMK-1 activity reported by Youngman et al. (2011)?

2. The noted relationship between fecundity and immunity may benefit from further discussion in the context of Miyata et al. (2008), which may be of relevance to the claims in the revised manuscript.

We thank the reviewer for pointing out these specifics that merit attention. We have revised the Discussion to include both studies and their implications on the relationship between immunity and fertility (Pg. 22; Para 2).

Reviewer #2 (Remarks to the Author):

I have re-reviewed the study, and **I am happy with the authors' response to my concerns. It's a very interesting, nice study.**

We are thankful to the reviewer for recognizing the significance and novelty of this study.

Reviewer #3 (Remarks to the Author):

Review of Amrit et al revision.
While the authors have addressed many of my comments, several regarding data presentation and analysis were left unchanged.

Figure 3A remains unchanged from the original submission. This figure shows the results of experiments testing the tcer-1 mutant and WT for survival following transfer to P. aeruginosa. Rather than showing the results from a single trial, figure 3B uses data for time points from various trials. However, only one out of seven trials gave a result where statistical significance was found at L4, Day 2, and Day 4 with no statistical significance at Days 6 and 9. Based on the original data presented in Table S6, the results are not nearly as clean cut as that presented in Figure 3A. I view this presentation as misleading. In their rebuttal, the authors acknowledge that this is "representative" data; however, this is not mentioned at all in the figure legend. A reader who does not explore the supplementary table will be given the impression that this trend is representative of the population. **I recommend** either removing figure 3A, **changing such that the results from a single trial are presented,** or adding a clear caveat to the figure legend that the various graphs are not from the same trial and to look at Fig S6.

As per the reviewer's suggestion, Fig. 3A has been modified to present the results from a single trial (#4).

Figure 3B remains unchanged from the original submission. It is still not clear whether it is appropriate to pool the data from the various trials. An alternative is to develop a statistical model that includes trial as a block effect. Perhaps a more laborious approach would be to use a series of comparisons between each of the trials for WT and ensure that they are not statistically different. Then do the same for tcer-1. If there are no differences among trials then proceed with pooling the data for figure 3B.

As per the reviewer's suggestion, Fig. 3B has been removed.

I am alarmed by the inclusion of outlier removal in the revision and mentioned in the rebuttal. The statistical analysis section of the methods now mentions that this was done “when applicable”. It is not clear where this was applicable. In general, I only recommend removing statistical outliers when there was an experimental or data entry error that would suggest the datum is not representative of the overall population variability.

The manuscript does not include any data now on which outlier analysis was conducted.

Outlier analysis was performed previously only for the thrashing analysis shown in Fig.8A. Upon re-analysis of this data with the outliers included, we found no difference in the statistical results or the conclusion derived from the data. The raw data used for the analyses with or without outliers, and results from both, are included at the end of this file.

Table S5 appears unchanged from the original submission. I was confused by the author’s rebuttal: “we grouped the strains such that the lifespans of the different transgenic strains in ‘fertile’ genetic backgrounds (wild type, tcer-1) were conducted simultaneously, whereas, those in the ‘sterile’ genetic backgrounds (glp-1, tcer-1;glp-1) were conducted together. Wild-type control strains were tested in every trial. This grouping is the reason for the ostensible duplication of the controls”. The first sentence suggests that N2 wild type was not tested in every trial; however, the second sentence suggests that they were included. I, therefore, don’t understand from this whether, for example, the 48 N2 animals mentioned in Row 24 of S5A are a different set than those mentioned in Row 56 of S5B? If the data were from different dates (and just by chance got the same result), then I think this table is fine. If, however, they are the same individuals, then presenting them separately seems disingenuous.

N2 animals (and other relevant controls) were tested in every trial.

The reason some controls appeared on more than one tab/worksheet is because they were part of a trial in which transgenic-strains with TCER-1 expression in different tissues were tested together. For eg., the N2 data referred to above was obtained in an experiment in which the following strains were run: N2, *glp-1, tcer-1;glp-1* and *tcer-1;glp-1* mutants carrying transgenes with TCER-1 expressed under (a) endogenous promoter, (b) intestinal promoter, or (c) muscle promoter (as well as non-transgenic control siblings of each). Since Tables S5A, S5B and S5D show the effect of TCER-1 expression when driven by endogenous, intestinal and muscle promoters, respectively, the data for the controls, N2, *glp-1* and *tcer-1;glp-1,* appear in the three tables.

We chose this format for presentation because it makes a very large and complex dataset accessible and, **importantly, it allows the readers to evaluate the consistency/reliability of the effect produced by TCER-1 expression in a given tissue (in a given genetic background).**

To increase the transparency, we have now added notes to each worksheet that identify clearly if the controls in a given trial were shared with a trial on another sheet because they were conducted as a single experiment (file NEW_TableS5).

We include here an alternative version of the table (file ALTERNATIVE_Table S5) in which the data is presented as individual trials. We believe it is clear that this form of presentation will diminish the ability of a reader to critically analyze the data. We hope the reviewer will agree with this assessment and the presentation of TableS5 as tissue-centered sub-tables/worksheets.

The authors recognize my critique of the Mantel Cox method, but suggest that it is the best option available to the field. I would argue that the best statistical test is the one that most likely detects true differences in the populations. It is possible that the Mantel-Cox method will detect those differences; however, if the assumptions for a statistical test are violated substantially then I don't see the logic in using it just because it is commonly used in the field. Were the assumptions tested? Much of the data show very clear differences without statistical analysis; however, if a formal analysis is included, it should be appropriate. Similar to my concern above about pooling data, there may be a more complex statistical models that will better for analyzing these data. An alternative is to not conduct a statistical test, but rather show the means with 95% confidence intervals at each time point.

The reviewer did not suggest a statistical test of their choice that is better than the Mantel-Cox method. Experimentally, we performed an alternative analysis proposed to be suitable for cases where curves overlap[1,2], the Taron-Ware test, on the data in Fig. 2 (where the overlapping curves are seen). The results of this test were overwhelmingly similar to those of Mantel-Cox analysis, as can be seen in the table included in this file. We would also like to note that most of the curves in our study do not intersect in the middle, so if Mantel-Cox assumptions are violated it is only for a small fraction of lifespans. Hence, while there may be more complex statistical models that can be created to analyze such data, they are beyond the scope/necessity of this study.

Following the reviewer's suggestion, we plotted the graphs in Fig. 2 to show the data with 95% confidence intervals and no statistical analysis. This option is included at the end of this file (ALTERNATIVE FIG. 2). It is clear that this format diminishes the clarity of the data significantly, whereas, the existing Fig. 2 emphasizes the nuances of the lifespan effects.

For these above reasons, we believe the use of Mantel-Cox statistics is suitable for the data in this manuscript. Additionally, as the reviewer points out, the qualitative differences between strains are obvious in much of the data. Finally, we submit that Mantel-Cox statistics should be retained not only because it will allow other researchers to compare their data with ours without requiring an enormous amount of re-analysis. While we completely agree with the reviewer that a method should not be employed simply because it is most commonly used in the field, there is undeniable value to data in an article being accessible for meaningful comparisons with other studies.

[1] Choosing statistical tests for survival analysis. Etikan İ, Bukirova K, Yuvalı M. Biom Biostat Int J. 2018;7(5):477–481. DOI: 10.15406/bbij.2018.07.00249
[2] On Distribution-Free Tests for Equality of Survival Distributions. Tarone, R.E. and Ware, J. (1977). Biometrika, 64, 156-160. https://doi.org/10.1093/biomet/64.1.156

Fig. 8A: Data for Average Number of Thrashes & Statistics WITHOUT Outlier Test

	N2		CF2166	
	Average (Trials 1 to 6)	St. Dev	Average (Trials 1 to 6)	St. Dev
Day 2	89.1	8.91421	106.25	6.90442269
	96.05	7.7356	113.6	4.93537179
	93.8	7.38023	101.95	8.20782682
	89.11	5.54672	98.7	2.65766023
	92.45	22.582	105	5.66615065
	97.25	3.4622	100.3	3.74306376
Day 5	82.7	20.1549	89.75	22.1950089
	89.75	6.59246	89	11.7159361
	88.4	31.1525	86.35	26.0257407
	87.95	6.32018	97.32	5.58820814
	95.05	6.60522	93.95	12.7958669
	96.15	8.2288	78.35	31.4145039
Day 7	71.6	25.5804	47.6	38.7724807
	68.55	22.5913	46.25	44.8093916
	71.25	29.1381	73.45	33.1672177
	56.3	38.0983	42.1	40.2255483
	87.2	10.7977	65.2	34.2138784
	77.5	33.043	55.25	35.6294896
Day 9	59.5	17.0371	28	34.154448
	48.4	27.5421	31	32.855238
	25.9	32.5693	31.26	34.5797663
	43.78	35.6759	34.85	38.9718834
	56.4	30.2	44.1	38.3061078
	64.47	29.6617	37	37.8197479

	Average		P value
Day	N2	*tcer-1*	
2	93.0	104.3	0.0028
5	90.0	89.1	0.6647
7	72.1	55.0	0.0182
9	49.7	34.4	0.0125

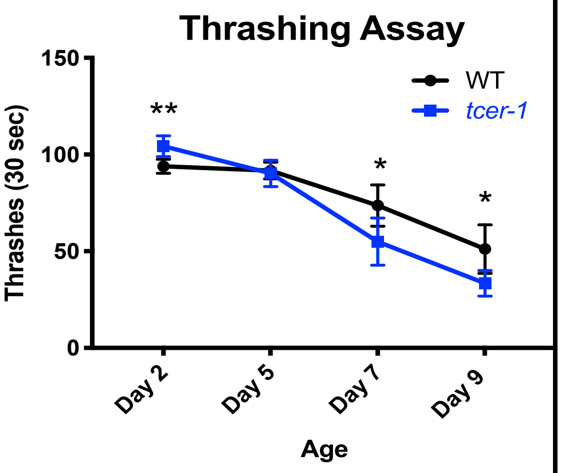

Fig. 8A: Data for Average Number of Thrashes & Statistics WITH Outlier Test

	N2		CF2166	
	Average (Trials 1 to 6)	St. Dev	Average (Trials 1 to 6)	St. Dev
Day 2	89.1	8.91421	106.25	6.90442269
	96.05	7.7356	113.6	4.93537179
	93.8	7.38023	101.95	8.20782682
	90.06	3.79585	98.7	2.65766023
	97.32	6.20083	105	5.66615065
	97.25	3.4622	100.3	3.74306376
Day 5	86.74	9.20653	89.75	22.1950089
	89.75	6.59246	89	11.7159361
	93.05	23.4802	90.74	17.1079808
	87.95	6.32018	97.32	5.58820814
	95.05	6.60522	96.37	7.02543416
	97.79	3.8381	78.35	31.4145039
Day 7	75.37	19.7715	47.6	38.7724807
	72.11	16.4887	46.25	44.8093916
	71.25	29.1381	73.45	33.1672177
	56.3	38.0983	42.1	40.2255483
	89.32	5.34429	65.2	34.2138784
	77.5	33.043	55.25	35.6294896
Day 9	62.58	10.3081	28	34.154448
	48.4	27.5421	31	32.855238
	31.26	34.5798	25.9	32.5693143
	43.78	35.6759	34.85	38.9718834
	56.4	30.2	44.1	38.3061078
	64.47	29.6617	37	37.8197479

	Average		P value
Day	N2	*tcer-1*	
2	93.9	104.3	0.0014
5	91.7	90.3	0.7985
7	73.6	55.0	0.025
9	51.1	33.5	0.0309

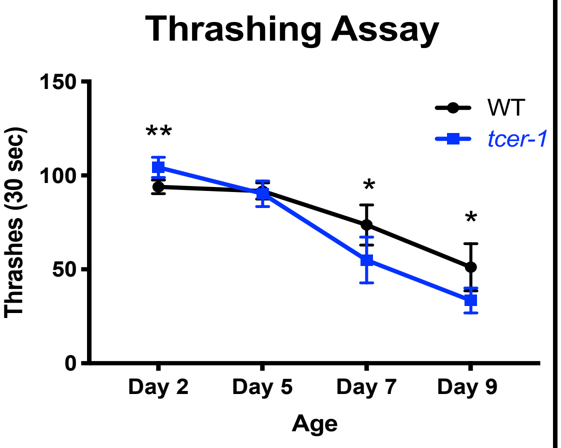

Comparison of Mantel-Cox and Tarone-Ware Statistical Tests for Lifespan Data in Fig. 2

TCER-1 TRANSGENIC STRAINS				
Fig. Panel	Log Rank Test		Tarone-Ware Test	
	Strains Compared	corrected_pvalue	Strains Compared	corrected_pvalue
2A	AGP173 vs *tcer-1*	1.80E-07	AGP173 vs *tcer-1*	4.00E-08
2B	AGP92 vs *tcer-1*	0	AGP92 vs *tcer-1*	0
2C	AGP170 vs *tcer-1*	0	AGP170 vs *tcer-1*	0
2D	AGP194 vs *tcer-1*	0	AGP194 vs *tcer-1*	0
2E	AGP120 vs *tcer-1*	5.10E-07	AGP120 vs *tcer-1*	1.60E-07
2F	AGP172 vs *tcer-1;glp-1*	0	AGP172 vs *tcer-1;glp-1*	0
2G	AGP91 vs *tcer-1;glp-1*	0	AGP91 vs *tcer-1;glp-1*	0
2H	AGP171 vs *tcer-1;glp-1*	0.0000038	AGP171 vs *tcer-1;glp-1*	0.0000012
2I	AGP79 vs *tcer-1;glp-1*	0	AGP79 vs *tcer-1;glp-1*	0
2J	AGP122 vs *tcer-1;glp-1*	0.0048	AGP122 vs *tcer-1;glp-1*	0.0075
2K	AGP172+ vs *tcer-1;glp-1*	0.0068	AGP172+ vs *tcer-1;glp-1*	0.01
2L	AGP91b+ vs *tcer-1;glp-1*	0.000007	AGP91b+ vs *tcer-1;glp-1*	0.000046
2M	AGP171 + vs *tcer-1;glp-1*	7.10E-07	AGP171 + vs *tcer-1;glp-1*	7.60E-07
2N	AGP79a + vs *tcer-1;glp-1*	4.90E-07	AGP79a + vs *tcer-1;glp-1*	2.00E-07
2O	AGP122a vs *tcer-1;glp-1*	0.000002	AGP122a vs *tcer-1;glp-1*	3.80E-07

CONTROL STRAINS				
Fig. Panel	Log Rank Test		Tarone-Ware Test	
	Strains Compared	corrected_pvalue	Strains Compared	corrected_pvalue
2A - 2F	N2 vs *tcer-1*	0	N2 vs *tcer-1*	0
2A - 2F	N2 vs *tcer-1;glp-1*	0	N2 vs *tcer-1;glp-1*	0.0000034
2A - 2F	N2 vs *glp-1*	0	N2 vs *glp-1*	0
2G, 2I and 2J	N2 vs *tcer-1;glp-1*	0.0002	N2 vs *tcer-1;glp-1*	0.0103
2G, 2I and 2J	N2 vs *glp-1*	0.0000079	N2 vs *glp-1*	0.00002
2H	N2 vs *tcer-1;glp-1*	0	N2 vs *tcer-1;glp-1*	0
2H	N2 vs *glp-1*	7.10E-08	N2 vs *glp-1*	0
2K and 2L	N2 vs *tcer-1;glp-1*	0.6915	N2 vs *tcer-1;glp-1*	0.3679
2K and 2L	N2 vs *glp-1*	0	N2 vs *glp-1*	4.90E-08
2M - 2O	N2 vs *tcer-1;glp-1*	0.1992	N2 vs *tcer-1;glp-1*	0.3222
2M - 2O	N2 vs *glp-1*	0	N2 vs *glp-1*	0

Genotypes of transgenic 'AGP' strains are detailed in Table S8 and Figure 2 Legend

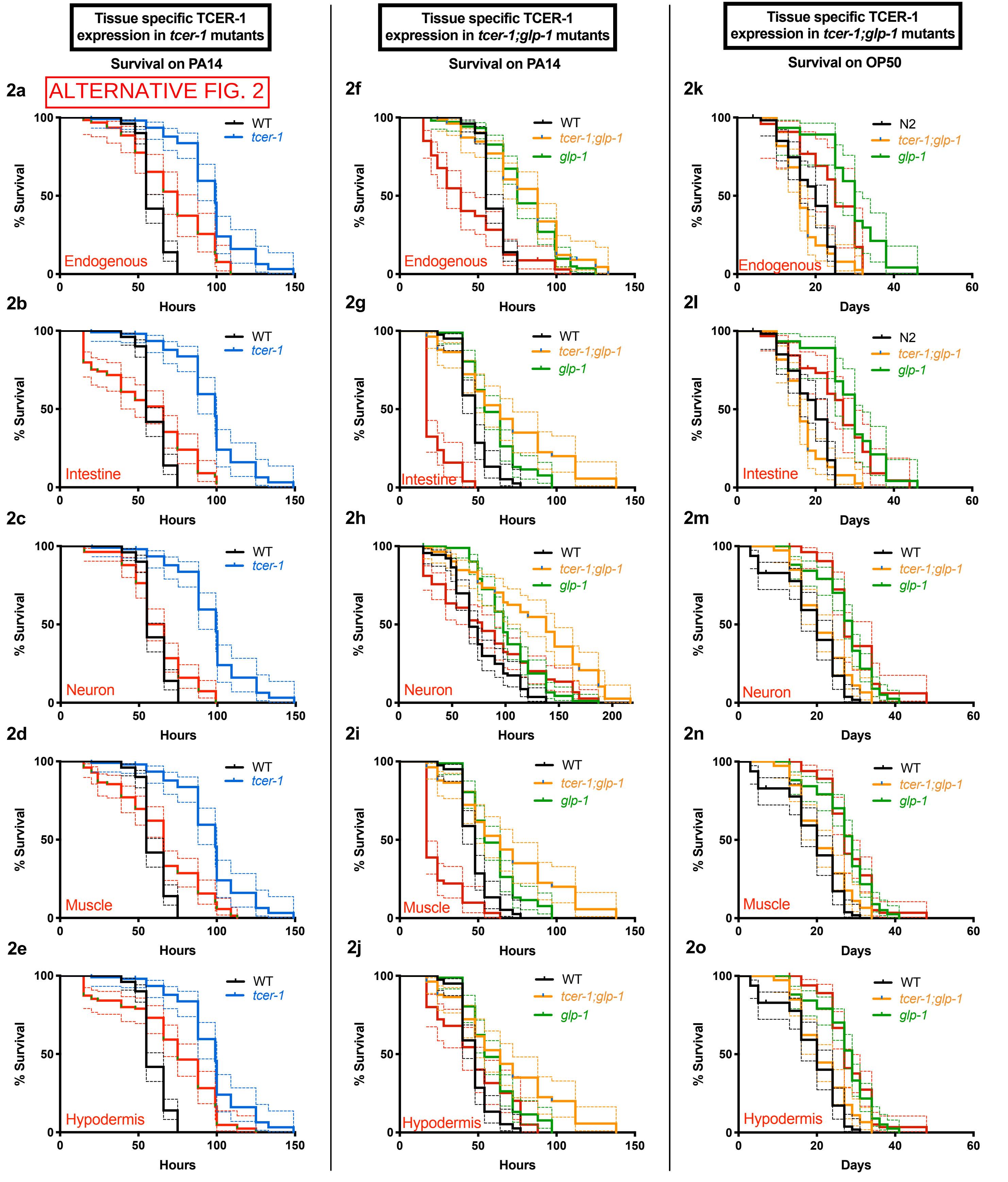

Tissue specific TCER-1 expression in tcer-1 mutants
Survival on PA14
Tissue specific TCER-1 expression in tcer-1;glp-1 mutants
Survival on PA14
Tissue specific TCER-1 expression in tcer-1;glp-1 mutants
Survival on OP50
ALTERNATIVE FIG. 2
2a
2b
2c
2d
2e
2f
2g
2h
2i
2j
2k
2l
2m
2n
2o
WT
tcer-1
tcer-1;glp-1
glp-1
N2
Endogenous
Intestine
Neuron
Muscle
Hypodermis
% Survival
Hours
Days

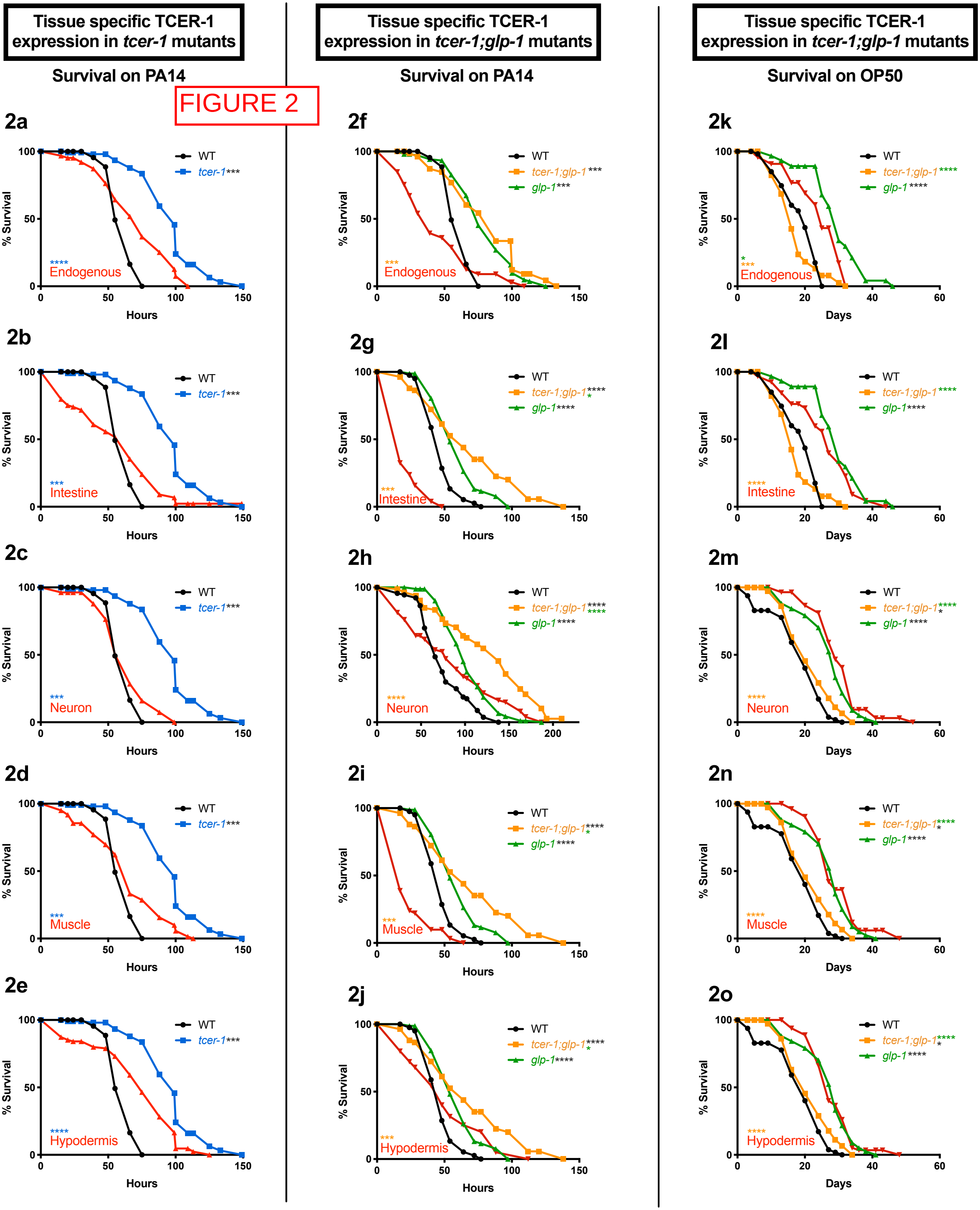

Tissue specific TCER-1 expression in tcer-1 mutants
Survival on PA14
Tissue specific TCER-1 expression in tcer-1;glp-1 mutants
Survival on PA14
Tissue specific TCER-1 expression in tcer-1;glp-1 mutants
Survival on OP50
FIGURE 2
2a
2b
2c
2d
2e
2f
2g
2h
2i
2j
2k
2l
2m
2n
2o
WT
tcer-1***
tcer-1;glp-1
glp-1
Endogenous
Intestine
Neuron
Muscle
Hypodermis
% Survival
Hours
Days

REVIEWERS' COMMENTS:

Reviewer #3 (Remarks to the Author):

The changes by the authors are satisfactory.
